# Systematic Structure–Activity Relationship Investigation on Novel 5‑Alkyl-6-benzyl-2-{[(methylthio/phenylthio)alkyl]thio}-pyrimidin-4(3*H*)‑ones Endowed with Potent Anti-HIV‑1 Activity

**DOI:** 10.1021/acsomega.6c08020

**Published:** 2026-09-16

**Authors:** Gebremedhin Solomon Hailu, Egor Degtyarenko, Emanuele Fabbrizi, Anastasia Golovina, Andrey Voronkov, Andrea Mancini, Maxim B. Nawrozkij, Vladimir Chernyshov, Laura Bavagnoli, Elena Bianchi, Roberta Astolfi, Rino Ragno, José A. Esté, Emmanuele Crespan, Francesco Fiorentino, Roman Ivanov, Antonello Mai, Dante Rotili

**Affiliations:** 1 Department of Science, Roma Tre University, 00146 Rome, Italy; 2 54744 Institute of Physiologically Active Compounds, Federal Research Center of Problems of Chemical Physics and Medicinal Chemistry, Russian Academy of Sciences, 142432 Chernogolovka, Moscow Region, Russia; 3 Department of Drug Chemistry and Technologies, 9311 Sapienza University of Rome, 00185 Rome, Italy; 4 Medicinal Biotechnology Department, 701584 Sirius University of Science and Technology, Olimpiyskiy Ave. 1, 354340 Sirius, Krasnodar Region, Russia; 5 Institute of Biophysics of the Future, Institutsky Lane, 9, Building 7, 141701 Dolgoprudny, Moscow Region, Russia; 6 Istituto di Genetica Molecolare IGM-CNR, Via Abbiategrasso 207, 27100 Pavia, Italy; 7 16760 Universitat Internacional de Catalunya, C/Josep Trueta s/n., 08195 Barcelona, Spain; 8 Department of Biochemical Sciences, 9311 Sapienza University of Rome, 00185 Rome, Italy; 9 Biostructures and Biosystems National Institute (INBB), 00165 Rome, Italy

## Abstract

Four series of 5-alkyl-6-benzyl-2-[(methylthio/phenylthio)­alkyl]­thiopyrimidin-4­(3*H*)-ones were systematically diversified at C2, C5, and C6 and evaluated as anti-HIV-1 agents. In MT-4 cells, these compounds displayed low-to-moderate cytotoxicity and subnanomolar-to-submicromolar inhibition of WT HIV-1, with potency governed by a balanced SAR. *Ortho*-dihalogenated C6-benzyl groups, particularly 2,6-F_2_ and 2,6-Cl_2_, were generally favored. In the 2,6-dihalobenzyl series, optimal activity required C5 methyl combined with *para*-substituted phenylthiomethyl groups at C2, whereas 3,5-Me_2_-benzyl analogues benefited from C5 isopropyl paired with methylthioethyl (**24l**) or unsubstituted phenylthiomethyl (**24m**) C2 side chains. The *p*-methoxyphenyl­thiomethyl thiothymine derivatives **21e** (2,6-F_2_) and **23e** (2,6-Cl_2_) emerged as lead inhibitors, showing high selectivity, potent activity against WT and clinically relevant HIV-1 variants, superior efficacy compared with nevirapine and efavirenz, dapivirine-like potency, and improved resistance tolerance relative to phenethyl-*S*-DABO bioisosteres. Together, biological and QSAR and docking data contribute to defining optimization strategies aimed at achieving durable anti-HIV-1 activity within the investigated chemotype and support further development of next-generation NNRTIs with improved efficacy against clinically relevant HIV-1 strains.

## Graphical abstract

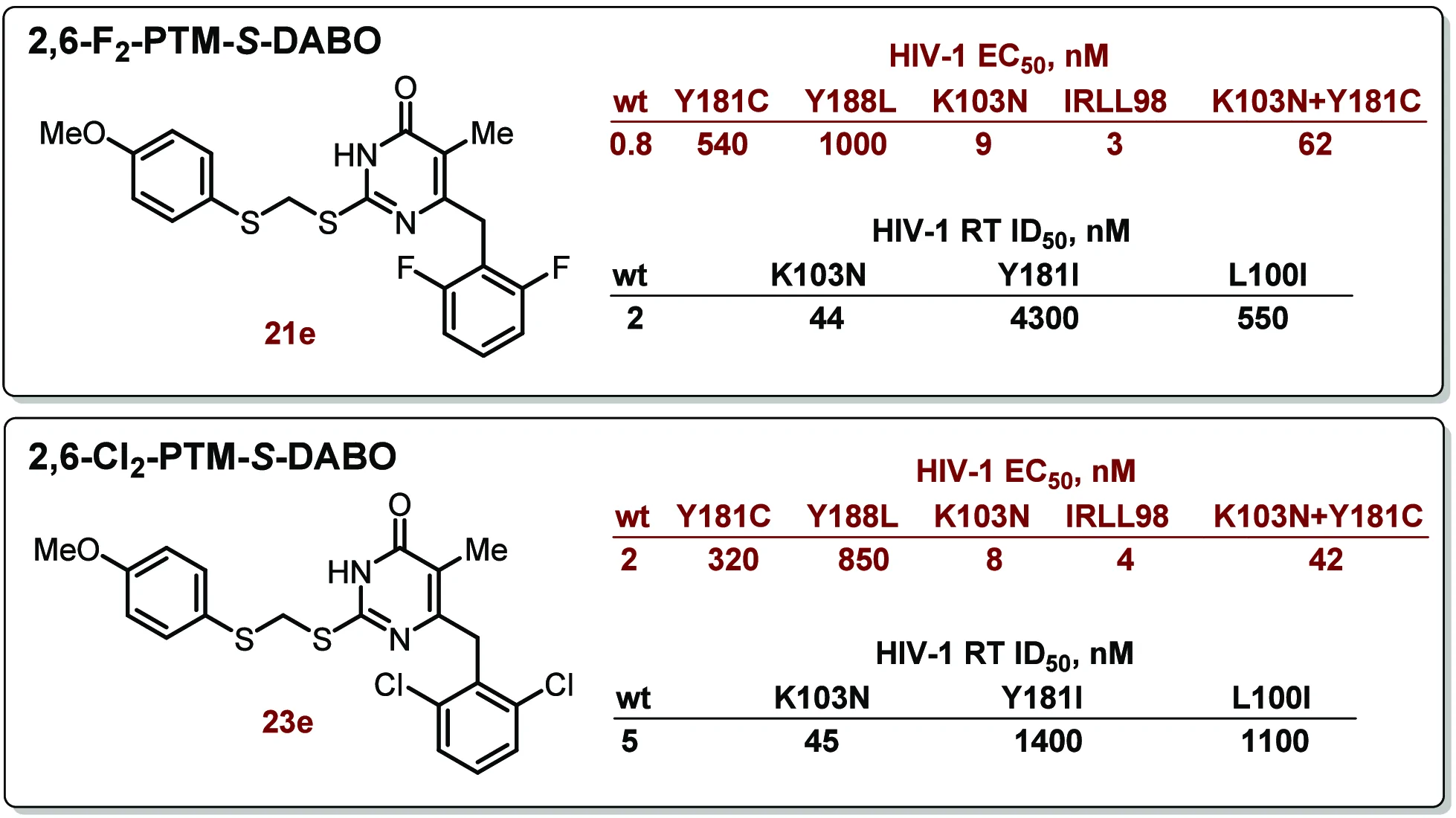

## Introduction

The 3,4-dihydro-2-alkoxy-6-benzyl-4-oxopyrimidines (DABOs),^1,2^ originally described by our group as HIV-1 NNRTIs structurally related to 1-[(2-hydroxyethoxy)­methyl]-6-(phenylthio)­thymine (HEPT), have long served as a platform for antiviral optimisation.^3^ Extensive SAR and molecular modeling studies identified the C2, C5, and C6 positions of the pyrimidin-4­(3*H*)-one scaffold as the main determinants for anti-HIV-1 activity.^4–7^

At the C2 position, appropriately sized lipophilic substituents are required for optimal anti-HIV-1 activity.^4,6,7^ In particular, alkoxy (DABOs, **1**),^2,8^ alkylthio (*S*-DABOs, **2**),^7,9–18^ alkylamino (*NH*-DABOs, **3**),^19,20^ dialkylamino/aza­(hetero)­cycloalkyl (*N*,*N*-DABOs, **4**)^21–26^ or cycloalkyl (*C*-DABOs, **5**),^4^ side chains consistently confer the highest HIV-1 inhibitory activity.

At the C6 position, the 2,6-difluorobenzyl moiety is generally preferred, as exemplified by F_2_-*S*-DABOs (**6**),^4,11–14,18^ conformationally restricted *S*-DABOs (**7**),^12,14,21,27–30^ F_2_-*NH*-DABOs (**3**),^19,20^ F_2_-*N*,*N*-DABOs (**4**),^21–25^ and DAPY-DABO hybrids (**8**).^23,31^ However, this trend is context-dependent: among conformationally restricted (cyclo)­alkyl-*S-*DABOs certain 6-[(2-Cl-6-F-phenyl)­ethyl]-2-(cycloalkylthio)-5-methylpyrimidin-4­(3*H*)-ones (**9**) are more potent than the corresponding 2,6-F_2_-phenyl-substituted (**10**) counterparts (Figure 1),^32^ while among conformationally flexible phenylalkyl-*S*-DABOs there are some 2,6-Cl_2_ derivatives (**11**) that are more potent against both WT and mutant HIV-1 strains than their 2,6-F_2_ counterparts (**12**) (Figure 2).^13^

**Figure 1:**
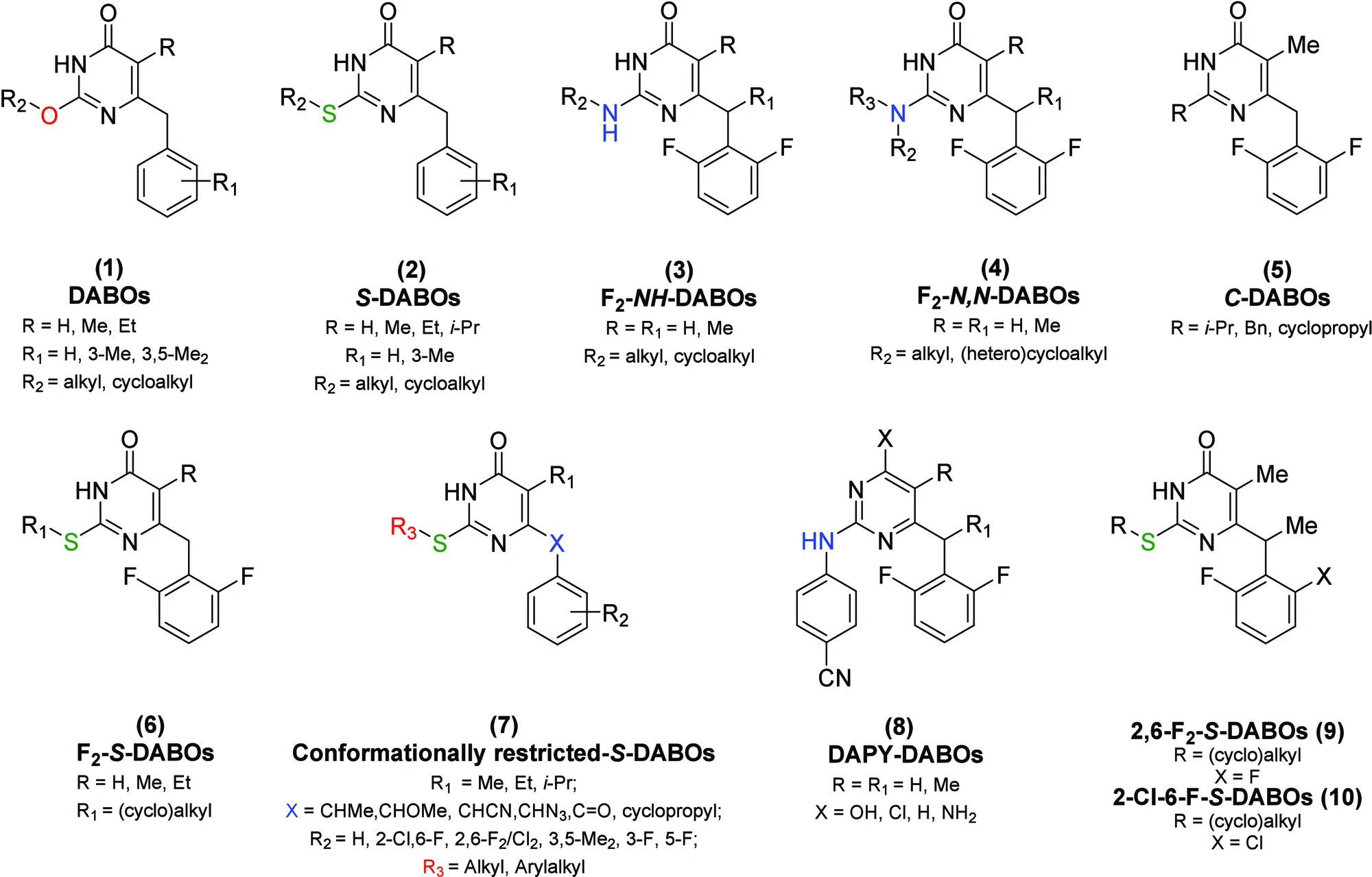
Most relevant DABO family compound series (**1**–**10**) disclosed over the years.

**Figure 2:**
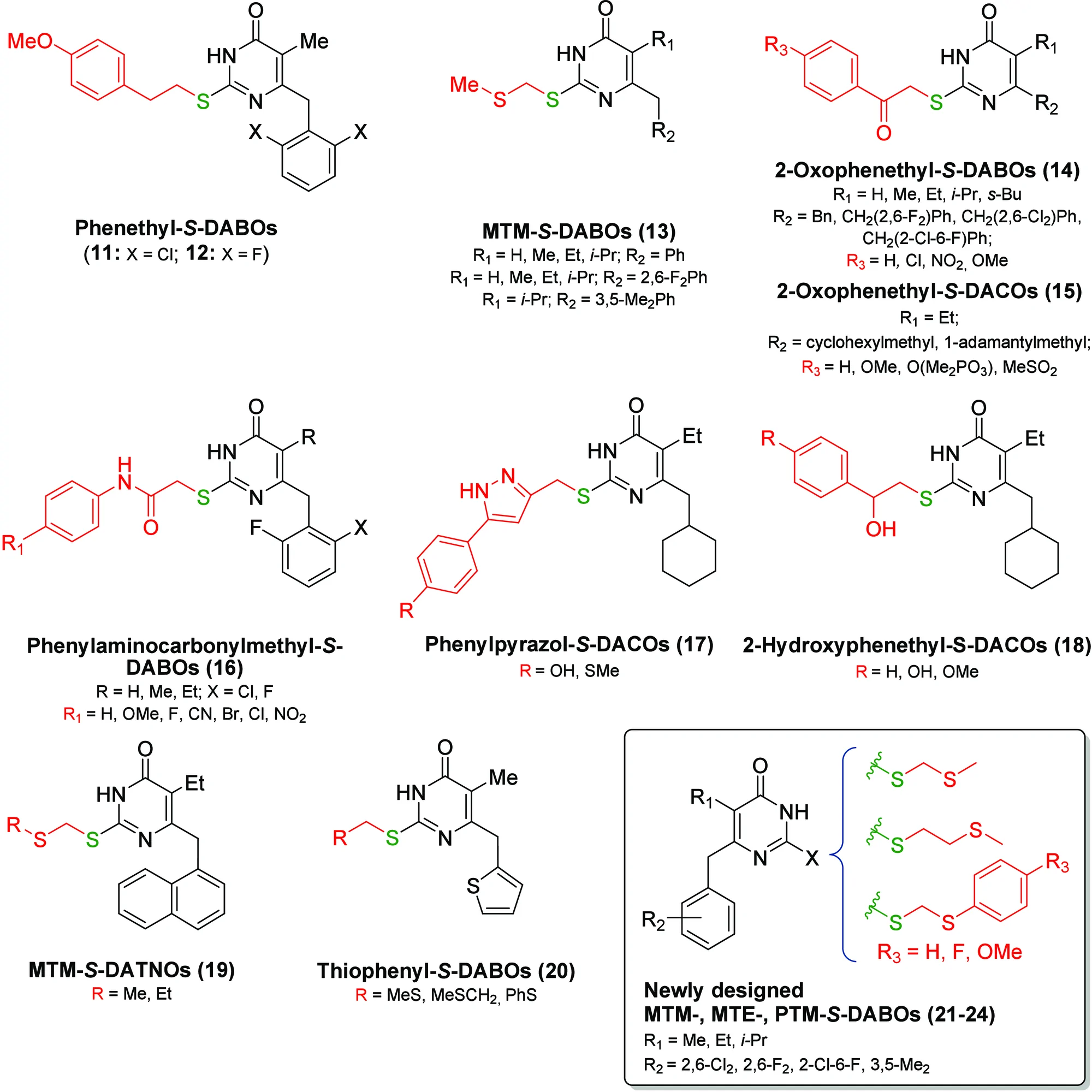
Background and rational design of the MTM-, MTE-, and PTM-*S*-DABOs (**21**–**24**) object of the present study.

Optimization at the C5 position confirmed that a methyl group is generally optimal in most DABO series, whereas pyrimidin-4­(3*H*)-one, 5-ethylpyrimidin-4­(3*H*)-one, and 5-isopropylpyrimidin-4­(3*H*)-one analogues typically exhibit reduced potency compared to the corresponding thymine congeners (Figure 1).^4,6,7,19,21–25^

SAR studies on C2 have generated several series of *S*-DABO analogues with potent anti-HIV-1 activity.^7,11,13,14,17,30,32–34^ Among them, 2-methylthiomethyl-*S*-DABOs (MTM-*S*-DABOs, **13**, Figure 2),^10,11,33,34^ bearing a methylthiomethyl (MTM) group on the exocyclic sulfur atom of the pyrimidine ring, and 2-oxophenethyl-*S*-DABOs (**14**, Figure 2)^17^ displayed a hybrid SAR profile combining features of HEPTs and (cyclo)­alkyl-*S*-DABOs.^7,10,33,35^ Like HEPTs, their potency often increased upon introduction of bulky lower alkyl substituents at C5 (*i*-Pr > Et > Me > H),^10,11,17,33^ but, at least for MTM-*S*-DABOs, declined with allyl,^36^ benzyl^37^ or phenyl^37^ groups at this position. Like (cyclo)­alkyl-*S*-DABOs, they still retained modest activity with a C5 hydrogen,^10,33^ and were sensitive to the C6 benzyl substitution,^10,11,17^ performing best in terms of potency and selectivity in the presence of a 2,6-dihalo substitution. Indeed, MTM-*S*-DABOs were inactive with C6 benzhydryl^38^ or aryl^39^ moieties, while the 2,6-F_2_ substitution on the C6 benzyl group^11^ and, in some cases, the 2-Cl-6-F one,^34^ markedly increased potency. Among 2-oxophenethyl-*S*-DABOs (**14**),^17,40–42^ the C5 ethyl/*iso*-propyl derivatives bearing a 2,6-dichlorobenzyl/2-chloro-6-fluorobenzyl moiety at C6 gave the highest broad-spectrum anti-HIV-1 activity and the lowest fold-resistance ratio.^17^ The 2-chloro-6-fluoro substitution was likewise optimal in the related phenylaminocarbonylmethyl-*S*-DABOs (**16**).^43,44^ The highly potent 2-phenylethyl-*S*-DABOs (**11**, **12**) of Botta and co-workers^13,14^ inspired numerous bioisosteric/regioisomeric analogues, including 2-oxophenethyl-*S*-DABOs (**14**),^17,40–42^ 2-oxophenethyl-*S*-DACOs (**15**),^45–48^ phenylaminocarbonylmethyl-*S*-DABOs (**16**),^43,44^ phenylpyrazol-*S*-DACOs (**17**),^49^ and 2-hydroxyphenethyl-*S*-DACOs (**18**),^50^ many of which inhibited HIV-1 replication at low nanomolar concentrations with low cytotoxicity (Figure 2), confirming that modulation of the C2 sulfur-linked side chain strongly affects antiviral activity and selectivity.

Previous work on dihydro-alkyl-thio-naphthylmethyl-oxopyrimidine (DATNO) thiouracil derivatives (**19**), showed that homologation from *S*-[(methylthio)­methyl] to *S*-[(ethylthio)­methyl] enhanced anti-HIV-1 activity,^35^ while 6-(thiophen-2-ylmethyl)-2-thiothymines (**20**)^16^ showed comparable potency for *S*-[(methylthio)­methyl] and *S*-[(phenylthio)­methyl] groups, with twofold higher activity for *S*-[(methylthio)­ethyl] analogues. However, these compounds remained less active than NNRTIs such as nevirapine (NVP), likely because replacing the C6 benzyl group with thiophen-2-ylmethyl reduces the activity as observed with (cyclo)­alkyl-*S*-DABOs.^5,7,51^

To the best of our knowledge, comprehensive SAR analyses on MTM-*S*-DABO analogues addressing the combined influence of C2-substituted thioether side chains, C5 alkyl substitution, and C6 arylmethyl patterns have not been reported yet. Moreover, biological evaluation against clinically relevant HIV-1 mutant strains and mutated RT remains scarce, representing a significant gap in the current understanding of this privileged *S*-DABO series.

We therefore carried out a systematic SAR study, against both WT and mutant HIV-1 strains and RTs, of MTM-*S*-DABOs (**21a**,**f**,**k**, **22a**,**f**,**k**, **23a**,**f**,**k**, **24a**,**f**,**k**) and new related derivatives (**21b**–**e**,**g**–**j**,**l**–**o**, **22b**–**e**,**g**–**j**,**l**–**o**, **23b**–**e**,**g**–**j**,**l**–**o**, **24b**–**e**,**g**–**j**,**l**–**o**, Figure 2), with modifications at several positions of the pyrimidinone core. At C2, alongside the parent MTM group, we introduced the superior homologue (methylthio)­ethyl substituent (MTE-*S*-DABOs) as well as (un)­substituted (phenylthio)­methyl (PTM-*S*-DABOs) side chains on the exocyclic sulfur atom to probe steric, conformational, and electronic effects. The PTM chains were designed as bioisosteres of both the 2-oxophenethyl^17^ and the 2-phenylethyl^13,14^ side chains and were decorated with *p*-F and *p*-OMe groups (Figure 2), that are known to confer high anti-HIV-1 potency across these *S*-DABOs.^7,13,17^ At the C6, we retained the highly effective benzyl moiety and included the most favorable substitution patterns, namely 2,6-F_2_, 2-Cl-6-F, and 2,6-Cl_2_.^7,12,13,17,18,32^ The 3,5-Me_2_-benzyl substitution was also comprised for comparative purposes, given its established contribution to antiviral activity both in HEPT derivatives^52^ and in selected 2-oxophenethyl-*S*-DABOs.^40^ Finally, C5 was varied with Me, Et, and *i*-Pr to modulate the stereoelectronic effects within the non-nucleoside binding site (NNBS) (Figure 2).

### Chemistry

Final compounds **21a**,^11^ **b**–**e**,**f**,^11^ **g**–**j**,**k**,^11^ **l**–**o**, **22a**–**o**, **23a**–**o**, and **24a**–**j**,**k**,^10^ **l**–**o** were prepared as outlined in Scheme 1. All 5-alkyl-6-(arylmethyl)-2-thiouracils intermediates **25a**,^18^ **b**,^11^ **c**,^11^ **d**–**h**,^53^ **i**,^17^ **j**,^54^ **k**,^51^ **l** ^51^ were prepared according to previously reported procedures. Subsequent *S*-alkylation of intermediates **25a**–**l** with the proper alkyl chlorides afforded the final compounds **21-24** via two alternative routes, depending on the nature of the chloride used.^55^ For the chloromethyl methyl sulfide, the appropriate 5-alkyl-6-(arylmethyl)-2-thiouracils (**25a**–**l**) were reacted with a two-fold molar excess of the alkylating agent in dry DMF at room temperature providing the final compounds **21a**,^11^ **f**,^11^ **k**,^11^ **22a**,**f**,**k**, **23a**,**f**,**k**, and **24a**,**f**,**k**.^10^ In contrast, chloroethyl methyl sulfide and (un)­substituted chloromethyl phenyl sulfides were reacted with the thiouracils **25a**–**l** in an ethanolic potassium hydroxide solution, first at room temperature and then under reflux, providing the final compounds **21b**–**e**,**g**–**j**,**l**–**o**, **22b**–**e**,**g**–**j**,**l**–**o**, **23b**–**e**,**g**–**j**,**l**–**o** and **24b**–**e**,**g**–**j**,**l**–**o**. All alkylating agents were commercially available except for the chloromethyl *p*-fluorophenyl sulfide^56^ and the chloromethyl *p*-methoxyphenyl sulfide,^57^ which were prepared according to the published Böhme reaction procedures. Chemical and physical characterization data of the newly-synthesized final compounds **21b**–**e**,**g**–**j**,**l**–**o**, **22a**–**o**, **23a**–**o**, and **24a**–**j**,**l**–**o**, together with the corresponding elemental analyses, are provided in Tables S1 and S2, respectively (Supporting Information Part 1).

**Scheme 1:**
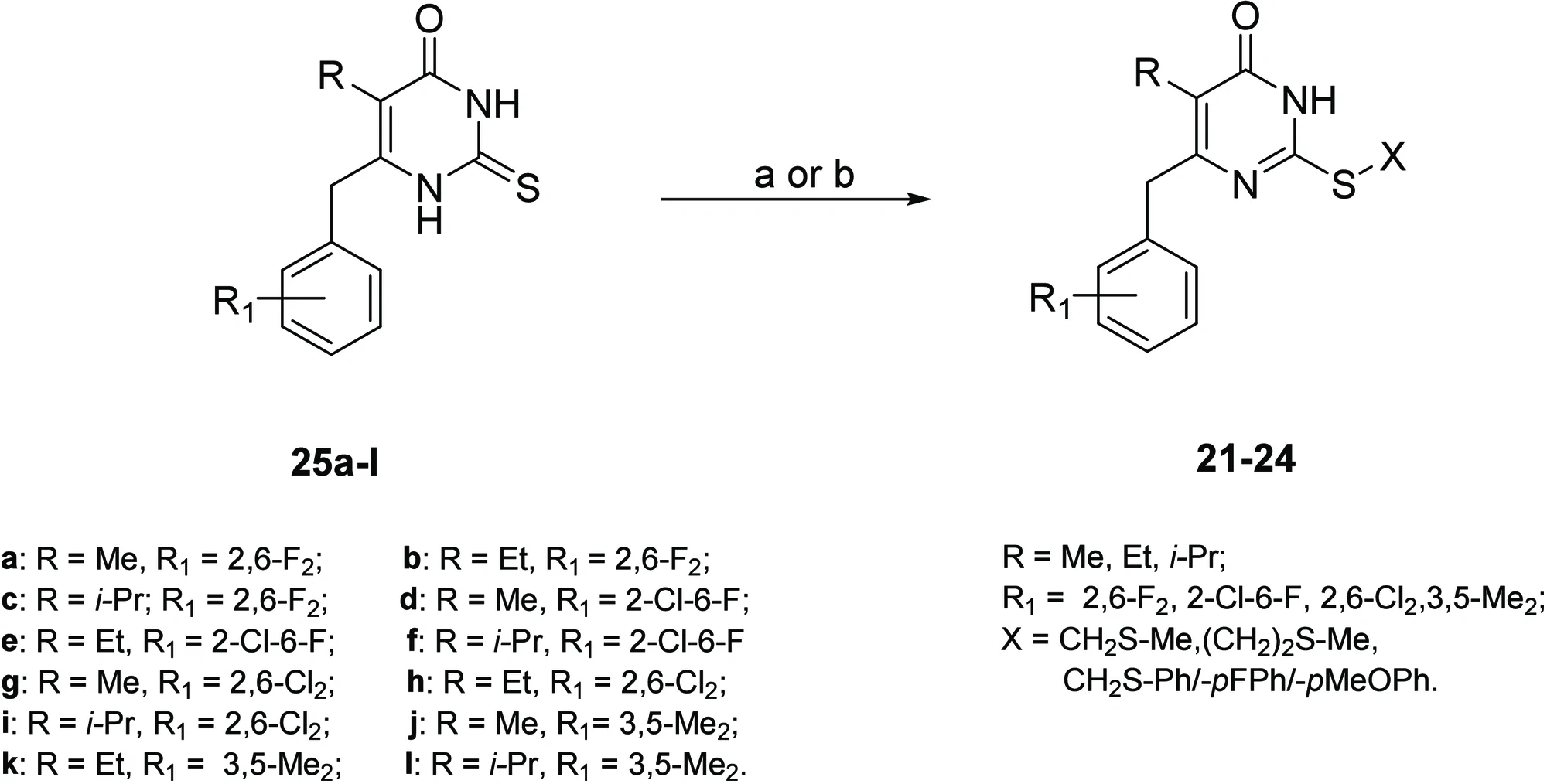
Synthesis of Compounds **21**–**24** ^a^

a Reagents and conditions: (a) chloromethyl methyl sulfide, dry DMF, rt; (b) potassium hydroxide, 95% ethanol, chloromethyl ethyl sulfide or properly (un)­substituted chloromethyl phenyl sulfide, rt then reflux.

## Results and Discussion

### Cytotoxicity and Wild Type HIV-1 Inhibiting Activity

The four series of 5-substituted 6-benzyl-2-phenyl/methylthioalkyl-*S*-DABO derivatives **21a**–**o** (2,6-F_2_), **22a**–**o** (2-Cl-6-F), **23a**–**o** (2,6-Cl_2_), and **24a**–**o** (3,5-Me_2_) were tested to evaluate their cytotoxicity (CC_50_) and capability to inhibit the WT HIV-1-induced cytopathic effect in lymphoid MT-4 cells (EC_50_; HIV-1 strain, NL4-3). Nevirapine (NVP), efavirenz (EFV), and the “second generation” NNRTI dapivirine were added as reference drugs. Since the new PTM-*S*-DABOs (**21c**–**e**,**h**–**j**,**m**–**o**, **22c**–**e**,**h**–**j**,**m**–**o**, **23c**–**e**,**h**–**j**,**m**–**o**, and **24c**–**e**,**h**–**j**,**m**–**o**) can be considered bioisosteric analogues of the 2-(*p*-methoxyphenyl)­ethyl-*S*-DABOs **11** ^13^ and **12**,^14^ previously reported data under the same experimental conditions for those compounds were also included for comparison (Table 1).

**Table 1:**
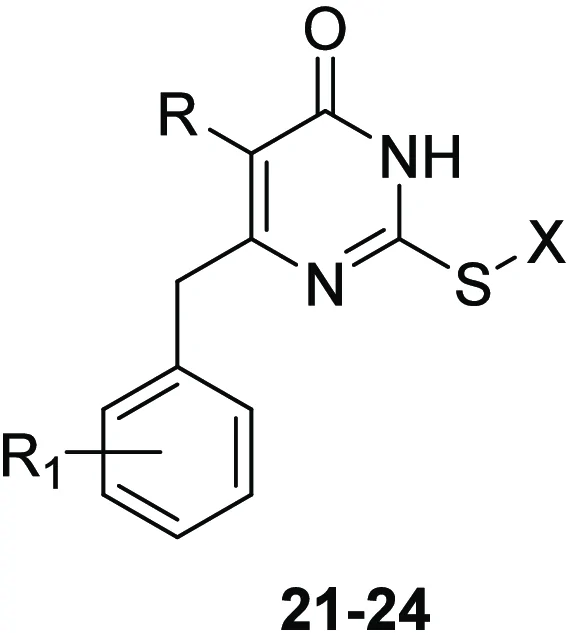
Cytotoxicity and Anti-HIV-1 Activity (WT HIV-1 Strain NL4-3) of *S*-DABOs **21**–**24** ^a^

Compd	R	R_1_	X	EC_50_,^b^ μM	CC_50_,^c^ μM	Selectivity Index^d^
**21a**	Me	2,6-F_2_	CH_2_SMe	0.006 ±0.0002	37±2	6183
**21b**	Me	2,6-F_2_	(CH_2_)_2_SMe	0.0029±0.0001	67±2	23161
**21c**	Me	2,6-F_2_	CH_2_SPh	0.004±0.0002	>64±3^e^	>16000
**21d**	Me	2,6-F_2_	CH_2_S(4-FPh)	0.002±0.0001	>61±2	>61200
**21e**	Me	2,6-F_2_	CH_2_S(4-MeOPh)	0.0008±0.00005	>59±3	>74250
**21f**	Et	2,6-F_2_	CH_2_SMe	0.002±0.00007	38±1	19250
**21g**	Et	2,6-F_2_	(CH_2_)_2_SMe	0.006±0.0003	>70±4	>70100
**21h**	Et	2,6-F_2_	CH_2_SPh	0.005±0.0003	>62±3	>12360
**21i**	Et	2,6-F_2_	CH_2_S(4-FPh)	0.009±0.0005	>59±2	>6578
**21j**	Et	2,6-F_2_	CH_2_S(4-MeOPh)	0.003±0.0001	>57±2	>19167
**21k**	*i*-Pr	2,6-F_2_	CH_2_SMe	0.0009±0.00004	54±1	60111
**21l**	*i*-Pr	2,6-F_2_	(CH_2_)_2_SMe	0.007±0.0003	**>**67±3	**>**9643
**21m**	*i*-Pr	2,6-F_2_	CH_2_SPh	0.012±0.0006	>59±1	>4975
**21n**	*i*-Pr	2,6-F_2_	CH_2_S(4-FPh)	0.025±0.0010	>57±1	>2292
**21o**	*i*-Pr	2,6-F_2_	CH_2_S(4-MeOPh)	0.033±0.001	>55±1	>1688
**22a**	Me	2-Cl-6-F	CH_2_SMe	0.029±0.002	>72±3	>2500
**22b**	Me	2-Cl-6-F	(CH_2_)_2_SMe	0.018±0.0007	>70±3	>3872
**22c**	Me	2-Cl-6-F	CH_2_SPh	0.020±0.001	>61±2	>3070
**22d**	Me	2-Cl-6-F	CH_2_S(4-FPh)	0.009±0.0003	>59±2	>6533
**22e**	Me	2-Cl-6-F	CH_2_S(4-MeOPh)	0.004±0.0002	>57±2	>19067
**22f**	Et	2-Cl-6-F	CH_2_SMe	0.020±0.001	>70±4	>3485
**22g**	Et	2-Cl-6-F	(CH_2_)_2_SMe	0.019±0.001	>67±3	>3526
**22h**	Et	2-Cl-6-F	CH_2_SPh	0.406±0.02	>59±4	>146
**22i**	Et	2-Cl-6-F	CH_2_S(4-FPh)	0.729±0.03	>57±3	>78
**22j**	Et	2-Cl-6-F	CH_2_S(4-MeOPh)	0.510±0.02	>55±2	>109
**22k**	*i*-Pr	2-Cl-6-F	CH_2_SMe	0.010±0.0004	>47±2	>4700
**22l**	*i*-Pr	2-Cl-6-F	(CH_2_)_2_SMe	0.594±0.02	>64±3	>109
**22m**	*i*-Pr	2-Cl-6-F	CH_2_SPh	0.874±0.04	>57±3	>66
**22n**	*i*-Pr	2-Cl-6-F	CH_2_S(4-FPh)	1.01±0.05	>55±2	>55
**22o**	*i*-Pr	2-Cl-6-F	CH_2_S(4-MeOPh)	0.743±0.03	>54±2	>72
**23a**	Me	2,6-Cl_2_	CH_2_SMe	0.111±0.005	>69±4	>623
**23b**	Me	2,6-Cl_2_	(CH_2_)_2_SMe	0.079±0.003	>66±3	>843
**23c**	Me	2,6-Cl_2_	CH_2_SPh	0.015±0.0006	>59±3	>3933
**23d**	Me	2,6-Cl_2_	CH_2_S(4-FPh)	0.005±0.0002	>57±3	>11320
**23e**	Me	2,6-Cl_2_	CH_2_S(4-MeOPh)	0.002±0.0001	>55±2	>27550
**23f**	Et	2,6-Cl_2_	CH_2_SMe	0.190±0.011	>67±2	>392
**23g**	Et	2,6-Cl_2_	(CH_2_)_2_SMe	0.565±0.022	>64±3	>114
**23h**	Et	2,6-Cl_2_	CH_2_SPh	0.594±0.025	30±2	51
**23i**	Et	2,6-Cl_2_	CH_2_S(4-FPh)	0.329±0.029	>55±2	167
**23j**	Et	2,6-Cl_2_	CH_2_S(4-MeOPh)	0.207±0.012	>53±3	>258
**23k**	*i*-Pr	2,6-Cl_2_	CH_2_SMe	0.283±0.007	>64±3	>227
**23l**	*i*-Pr	2,6-Cl_2_	(CH_2_)_2_SMe	0.445±0.02	>62±3	>139
**23m**	*i*-Pr	2,6-Cl_2_	CH_2_SPh	0.452±0.02	>55±3	>126
**23n**	*i*-Pr	2,6-Cl_2_	CH_2_S(4-FPh)	0.831±0.04	>53±2	>64
**23o**	*i*-Pr	2,6-Cl_2_	CH_2_S(4-MeOPh)	0.561±0.03	>52±2	>93
**24a**	Me	3,5-Me_2_	CH_2_SMe	1.84±0.11	>78±4	>42
**24b**	Me	3,5-Me_2_	(CH_2_)_2_SMe	0.209±0.01	>75±4	>357
**24c**	Me	3,5-Me_2_	CH_2_SPh	0.736±0.03	>65±2	>89
**24d**	Me	3,5-Me_2_	CH_2_S(4-FPh)	3.2±0.20	>62±3	>20
**24e**	Me	3,5-Me_2_	CH_2_S(4-MeOPh)	4.1±0.20	>61±3	>15
**24f**	Et	3,5-Me_2_	CH_2_SMe	0.778±0.03	>75±3	>96
**24g**	Et	3,5-Me_2_	(CH_2_)_2_SMe	0.180±0.01	>72±3	>179
**24h**	Et	3,5-Me_2_	CH_2_SPh	0.380±0.02	>63±2	>166
**24i**	Et	3,5-Me_2_	CH_2_S(4-FPh)	0.705±0.03	>59±3	>83
**24j**	Et	3,5-Me_2_	CH_2_S(4-MeOPh)	0.720±0.03	>60±2	>83
**24k**	i-Pr	3,5-Me_2_	CH_2_SMe	0.115±0.006	>72±3	>623
**24l**	*i*-Pr	3,5-Me_2_	(CH_2_)_2_SMe	0.027±0.001	>69±4	>2554
**24m**	*i*-Pr	3,5-Me_2_	CH_2_SPh	0.049±0.002	>61±2	>1242
**24n**	*i*-Pr	3,5-Me_2_	CH_2_S(4-FPh)	0.056±0.002	27±1	474
**24o**	*i*-Pr	3,5-Me_2_	CH_2_S(4-MeOPh)	0.082±0.003	33±1	396
**11** ^13^	Me	2,6-Cl_2_	CH_2_CH_2_(4-MeOPh)	<0.00014±0.000007	>57±3	>407142
**12** ^14^	Me	2,6-F_2_	CH_2_CH_2_(4-MeOPh)	0.0049±0.0003	>62±3	>12694
NVP				0.12±0.006	>7	>58
EFV				0.007±0.0003	>3	>429
Dapivirine				0.0006±0.00003	3	5000

a Values are means ± SD determined from at least two independent experiments, each one done in triplicate.

b Effective concentration 50, concentration needed to inhibit by 50% the HIV-1-induced cytopathic effect, evaluated with the MTT method in MT-4 cells (HIV-1 strain: NL4-3).

c Cytotoxic concentration 50, concentration needed to induce 50% death of noninfected cells, evaluated with the MTT method in MT-4 cells.

d Selectivity index (SI), CC_50_/EC_50_.

e Higher concentrations could not be tested due to precipitation of compounds in the culture medium.

All tested compounds **21**-**24** exhibited low-to-mild cytotoxicity, with CC_50_ values ranging from 27 μM to >78 μM and selectivity indexes (SI = CC_50_/EC_50_) between >15 and >74250 (Table 1). Most compounds showed good-to-excellent activity against WT HIV-1 with EC_50_ values ranging from sub-nanomolar to sub-micromolar in MT-4 infected cells, except for 3,5-Me_2_-benzyl compounds **24a**,**d**,**e** which displayed single-digit micromolar values.

Evaluation of compounds **21**–**24** against WT HIV-1 NL4-3 strain revealed a highly substitution-dependent antiviral profile, in which the nature of the C6 benzyl moiety, the C5 alkyl substituent, and the C2 thioether side chain sensibly influenced anti-HIV-1 potency and selectivity. Indeed, overall data showed that for any given substitution pattern at both C2 and C5 positions the highest anti-HIV-1 activity was displayed by the compounds of the C6 2,6-F_2_-benzyl series **21a**–**o**, while the rank order of potency across the remaining series varied depending on the profile of substitution at both C2 and C5 positions. The higher activity of the 2,6-F_2_-benzyl series **21a**–**o** is consistent with the results of the QSAR analysis, which highlighted a favorable contribution of van der Waals interactions associated with this substitution pattern (see 2D-QSAR section). More specifically, the higher potency of C6-2,6-dihalobenzyl analogues compared with their 3,5-dimethylbenzyl analogues is consistent with optimized steric and electronic complementarity within the NNBS, wherein the electron-deficient benzyl ring is expected to engage in favorable π-stacking with the electron-rich aromatic side chain lining the pocket.^17,18,33^ Within the 2,6-dihalobenzyl subset, the superior activity of the 2,6-F_2_ derivatives over the corresponding 2,6-Cl_2_ and 2-Cl-6-F congeners further agrees with the QSAR findings and the smaller van der Waals volume of fluorine, which may allow tighter packing against lipophilic pocket boundaries.^11,32^

The influence of the C5 substituent was likewise highly context-dependent and closely coupled to the stereoelectronic properties of the C6 benzyl fragment and the C2 thioether substituent. In the 6-(2,6-dihalobenzyl)-2-thiouracil series **21** and **22**, all MTE and PTM analogues followed the classical SAR trend of (cyclo)­alkyl-*S*-DABO, with activity increasing in the sequence *i-*Pr < Et < Me, while the MTM analogues (**21a**,**f**,**k** and **22a**,**f**,**k**, respectively) showed the already known opposite “HEPT-like” order of potency (*i*-Pr > Et > Me) confirming the favorable effect of bulky C5 substituents in this context (Table 1). In the 2,6-Cl_2_-benzyl series **23** the C5 position SAR trend was *i-*Pr < Et < Me with the MTM, MTE, and PTM derivatives, with the only partial exception of the C5 ethyl derivatives **23g** and **23h** that resulted less potent than the corresponding C5 isopropyl counterparts **23l** and **23m**, respectively (Table 1). In contrast, the 3,5-Me_2_-benzyl series (**24a**–**o**) consistently exhibited a “HEPT-like” trend, irrespective of the substitution at the C2 position, in which potency progressively increased with the size of the C5 substituents from 2-thiothymines (**24a**–**e**) derivatives to the corresponding 5-isopropyl-2-thiouracils (**24k**–**o**) (Table 1).

In this context, the activity trend reflects a balance between steric volume and binding orientation. C5-Me is optimal in the 2,6-dihalobenzyl/PTM congeners, in which the combined steric bulk of the C6 aryl and extended C2 thioether approaches the cavity limit, whereas larger alkyls incur localized crowding.^7,17,32^ When C2 is occupied by the smaller MTM group, or when the C6 aryl ring is a 3,5-Me_2_-benzyl motif, the C5-adjacent subpocket is underfilled, and i-Pr restores van der Waals contact without prohibitive penalty,^7,27,29,32^ consistent with the observed “HEPT-like” behavior.^7,10^

The exocyclic C2 thioether substituent influenced the antiviral potency in a context-dependent manner. For any given pattern of C5/C6 substitution, the extension by one methylene unit of the C2 side chain of MTM derivatives (**21a**,**f**,**k**, **22a**,**f**,**k**, **23a**,**f**,**k**, and **24a**,**f**,**k**) to afford the closest superior homologous MTE *S*-DABO counterparts (**21b**,**g**,**l**, **22 b**,**g**,**l**, **23 b**,**g**,**l**, and **24b**,**g**,**l**), resulted in a slight increase of activity, with the only exceptions of the 5-ethyl/isopropyl 6-(2,6-dihalobenzyl)-2-thiouracil derivatives (**21l**, **22l**, and **23g**,**l**), and in a markedly different SAR profile. In contrast, the substitution of the MTM side chain at C2 with phenylthiomethyl groups provided PTM-*S*-DABO derivatives that, if substituted with a MeO group or F atom at *para* position of the phenyl ring, displayed the highest potency and selectivity in the 5-methyl 6-(2,6-dihalobenzyl)-2-thiouracil series **21c**,**d**,**e**, **22c**,**d**,**e**, and **23c**,**d**,**e**. Indeed, among the 2-thiothymine analogues of the 2,6-F_2_- and 2-Cl-6-F-benzyl series (**21a**–**e** and **22a**–**e**) activity decreased in the order *p*MeOPhSCH_2_ > *p*FPhSCH_2_ > MTE > PhSCH_2_ > MTM, while among the 2-thiothymines of the 2,6-Cl_2_-benzyl series (**23a**–**e**) the order was *p*MeOPhSCH_2_ > *p*FPhSCH_2_ > PhSCH_2_ > MTE > MTM, and considering the only PTM moiety substitution profile the trend of potency was always *p*MeOPhSCH_2_ > *p*FPhSCH_2_ > PhSCH_2_. In contrast, within the 6-(3,5-Me_2_-benzyl)-2-thiouracils series (**24**), anti-HIV-1 activity decreased in the order MTE > PhSCH_2_ > *p*FPhSCH_2_ > *p*MeOPhSCH_2_ > MTM for any given substitution at C5. Notably, within the PTM subset, the potency trend was reversed compared with the 2,6-dihalobenzyl series, following the order PhSCH_2_ > *p*-FPhSCH_2_ > *p*-MeOPhSCH_2_.

In this regard, the modest potency gain from MTM to MTE reflects improved side-chain accommodation within the lipophilic NNBS tunnel, whereas the larger gain by PTM is consistent with additional aromatic interactions. In the 2,6-dihalobenzyl series, the *p*-MeO-PTM moiety markedly enhances potency through a synergistic combination of increased π-electron density, a critical hydrogen-bond acceptor site at the methoxy oxygen, and enhanced local Me lipophilicity,^7,11,14^ in accordance with the MFTA-QSAR analysis (see below). The *p-*fluoro analogue supports intermediate activity by retaining aromatic and hydrophobic character while lacking the MeO hydrogen-bond acceptor and resonance donation. In the 3,5-Me_2_ series, altered scaffold geometry repositions the C2 vector, penalizing bulky *para*-substituted PTMs via localized steric repulsion and shifting preference toward compact MTE or unsubstituted PTM chains.^7,14,17^

Overall, the data showed that optimal anti-HIV-1 activity requires precise balancing of steric volume and electronic properties across all three substitution vectors. Except for the previously reported MTM-*S*-DABOs **21a**,^11^ **f**,^11^ **k**,^11^ the most potent among the new compounds were all *p*-MeO- and, to a lesser extent, *p*-F-substituted PTM-*S*-DABOs belonging to the 6-(2,6-dihalobenzyl)-2-thiothymine series (**21d**,**e**, **22d**,**e**, and **23d**,**e**), with the greatest potency displayed by the 2,6-F_2_-benzyl and, to a lesser extent, 2,6-Cl_2_-benzyl *p*-MeO PTM-*S*-DABO derivatives **21e** and **23e**, respectively. Compared with the reference drugs, **21e** and **23e** exhibited subnanomolar to low single-digit nanomolar activity against WT HIV-1 (EC_50_ = 0.8–2 nM), outperforming both NVP and EFV in terms of potency, cytotoxicity, and selectivity, and showing activity substantially comparable to that of dapivirine. Interestingly, the 2,6-F_2_-benzyl derivative **21e** was more than 6-fold more potent than its bioisosteric counterpart **12**,^14^ whereas the 2,6-Cl_2_-benzyl analogue **23e** was more than 10-fold less active than the corresponding bioisostere **11** ^13^ (Table 1).

The 3,5-Me_2_-benzyl series (**24**) was generally the least potent and selective. However, **24l** and **24m**, which bear an isopropyl group at the C5 position and an MTE or PTM side chain at C2, respectively, partially restored anti-HIV-1 activity, with potency in the low double-digit nanomolar range. This activity was superior to that of NVP, but lower than those of both EFV and dapivirine (Table 1).

### Inhibitory Activity against Clinically Relevant HIV-1 Mutant Strains

To evaluate their resistance profile, MTM-, MTE-, and PTM-*S*-DABOs **21**–**24** were then tested against a panel of clinically relevant HIV-1 mutant strains (Y181C, Y188L, and K103N). Selected compounds (**21d**,**e**, **22d**,**e**, **23d**,**e**, and **24l**,**m**), exhibiting a favorable activity against WT HIV-1, were also assayed for their efficacy against the HIV-1 clinical isolate IRLL98 and the double-mutant K103N+Y181C. Finally, the performance of **21**–**24** was compared to that of NVP, EFV, dapivirine, and the reference phenethyl *S*-DABOs **11** ^13^ and **12** ^14^ (Table 2).

**Table 2:**
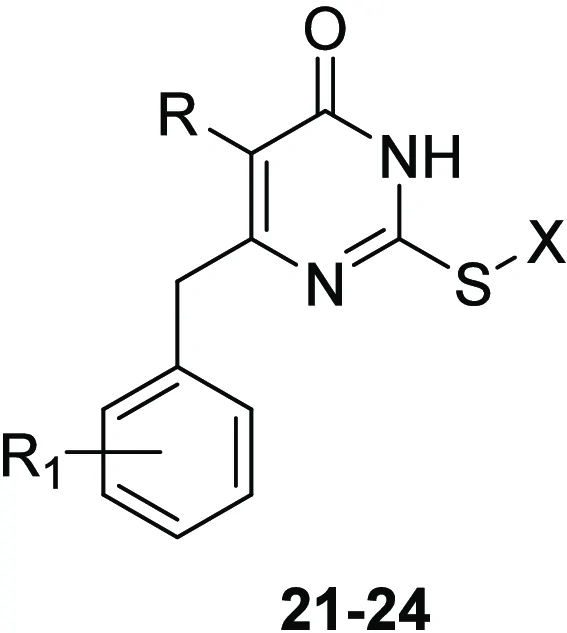
Anti-HIV-1 Activity of 21-24 against Clinically Relevant HIV-1 Mutant Strains^a^

				EC_50_,^b^ μM (*fold resistance*)^c^				
Compd	R	R_1_	X	IRLL98^d^	Y181C	Y188L	K103N	K103N+Y181C
**21a**	Me	2,6-F_2_	CH_2_SMe	ND^e^	8±0.4(*1283*)	>37±2^f^	6.5±0.3 (*1083*)	ND
**21b**	Me	2,6-F_2_	(CH_2_)_2_SMe	ND	2±0.1 (*827*)	9.5±0.4 (*3276*)	0.42±0.02 (*145*)	ND
**21c**	Me	2,6-F_2_	CH_2_SPh	ND	1±0.05 (*275*)	3±0.1 (*775*)	0.1±0.005 (*26*)	ND
**21d**	Me	2,6-F_2_	CH_2_S(4-FPh)	0.008±0.0004 (*4*)	0.75±0.03 (*375*)	2±0.1 (*1050*)	0.05±0.002 (*27*)	0.15±0.006 (*75*)
**21e**	Me	2,6-F_2_	CH_2_S(4-MeOPh)	0.003±0.0002 (*4*)	0.54±0.02 (*675*)	1±0.05 (*1375*)	0.009±0.0004 (*11*)	0.062±0.003 (*77*)
**21f**	Et	2,6-F_2_	CH_2_SMe	ND	2.5±0.1(*1250*)	>38±3	2.2 ±0.1(*1100*)	ND
**21g**	Et	2,6-F_2_	(CH_2_)_2_SMe	ND	3±0.1 (*517*)	10±0.6 (*1717*)	0.9±0.04 (*150*)	ND
**21h**	Et	2,6-F_2_	CH_2_SPh	ND	>62±2	>62±3	>62±3	ND
**21i**	Et	2,6-F_2_	CH_2_S(4-FPh)	ND	2±0.1 (*2322*)	>59±3	15±1 (*1667*)	ND
**21j**	Et	2,6-F_2_	CH_2_S(4-MeOPh)	ND	1.5±0.07 (*500*)	>57±2	2.7±0.1 (*900*)	ND
**21k**	*i*-Pr	2,6-F_2_	CH_2_SMe	ND	2±0.1(*2333*)	28±2(*31444*)	2±0.1(*2111*)	ND
**21l**	*i*-Pr	2,6-F_2_	(CH_2_)_2_SMe	ND	6±0.3 (*800*)	>68±4	37±2 (*5300*)	ND
**21m**	*i*-Pr	2,6-F_2_	CH_2_SPh	ND	>60±3	>60±4	>60±3	ND
**21n**	*i*-Pr	2,6-F_2_	CH_2_S(4-FPh)	ND	2.6±0.1 (*104*)	>57±2	>57±3	ND
**21o**	*i*-Pr	2,6-F_2_	CH_2_S(4-MeOPh)	ND	4±0.2 (*124*)	>58±3	5.8 ±0.3 (*176*)	ND
**22a**	Me	2-Cl-6-F	CH_2_SMe	ND	6.5±0.3 (*224*)	>73±4	3.8±0.2 (*131*)	ND
**22b**	Me	2-Cl-6-F	(CH_2_)_2_SMe	ND	5±0.3 (*289*)	40±3 (*2222*)	2.8±0.2 (*155*)	ND
**22c**	Me	2-Cl-6-F	CH_2_SPh	ND	9.5±0.5 (*475*)	13±0.6 (*665*)	2.2±0.1 (*110*)	ND
**22d**	Me	2-Cl-6-F	CH_2_S(4-FPh)	0.027±0.0002 (*3*)	1.2±0.04 (*133*)	5±0.3 (*589*)	0.095±0.004 (*10*)	0.25±0.01 (*28*)
**22e**	Me	2-Cl-6-F	CH_2_S(4-MeOPh)	0.008±0.0004 (*2*)	0.75±0.04 (*187*)	2.2±0.1 (*550*)	0.025±0.001 (*6*)	0.075±0.003 (*19*)
**22f**	Et	2-Cl-6-F	CH_2_SMe	ND	4.4±0.2 (*220*)	>70±4	3.2±0.1 (*160*)	ND
**22g**	Et	2-Cl-6-F	(CH_2_)_2_SMe	ND	3.8±0.2 (*200*)	38±3 (*1995*)	2±0.1 (*111*)	ND
**22h**	Et	2-Cl-6-F	CH_2_SPh	ND	12±0.6 (*30*)	>59±4	>59±3	ND
**22i**	Et	2-Cl-6-F	CH_2_S(4-FPh)	ND	8±0.4 (*11*)	18±1 (*25*)	>57±3	ND
**22j**	Et	2-Cl-6-F	CH_2_S(4-MeOPh)	ND	3.0±0.2 (*6*)	15±2	>55±3	ND
**22k**	*i*-Pr	2-Cl-6-F	CH_2_SMe	ND	3±0.1 (*330*)	>47±3	2.5±0.1 (*250*)	ND
**22l**	*i*-Pr	2-Cl-6-F	(CH_2_)_2_SMe	ND	9±0.4 (*15*)	47±2 (*79*)	>65±3	ND
**22m**	*i*-Pr	2-Cl-6-F	CH_2_SPh	ND	>58±3	>58±2	>58±3	ND
**22n**	*i*-Pr	2-Cl-6-F	CH_2_S(4-FPh)	ND	14±0.3 (*14*)	19±2 (*19*)	>65±3	ND
**22o**	*i*-Pr	2-Cl-6-F	CH_2_S(4-MeOPh)	ND	4±0.2 (*6*)	2.6±0.1 (*3*)	>54±3	ND
**23a**	Me	2,6-Cl_2_	CH_2_SMe	ND	>69±4	>69±4	>69±4	ND
**23b**	Me	2,6-Cl_2_	(CH_2_)_2_SMe	ND	7.0±0.3 (*89*)	34±2 (*432*)	48±3 (*611*)	ND
**23c**	Me	2,6-Cl_2_	CH_2_SPh	ND	1.0±0.05 (*87*)	3.1±0.1 (*207*)	0.1±0.005 (*7*)	ND
**23d**	Me	2,6-Cl_2_	CH_2_S(4-FPh)	0.017±0.0009 (*3*)	0.97±0.04 (*195*)	2.4 ±0.1 (*480*)	0.065 ±0.002 (*13*)	0.21±0.01 (*42*)
**23e**	Me	2,6-Cl_2_	CH_2_S(4-MeOPh)	0.004±0.0002 (*2*)	0.32±0.01 (*160*)	0.85±0.04 (*425*)	0.008±0.0004 (*4*)	0.042±0.002 (*21*)
**23f**	Et	2,6-Cl_2_	CH_2_SMe	ND	29±2 (*151*)	>67±3	>67±4	ND
**23g**	Et	2,6-Cl_2_	(CH_2_)_2_SMe	ND	2.5±0.1 (*4*)	4.0±0.2 (*6*)	28±2 (*49*)	ND
**23h**	Et	2,6-Cl_2_	CH_2_SPh	ND	5.0±0.2 (*8*)	17±1 (*29*)	>30±2	ND
**23i**	Et	2,6-Cl_2_	CH_2_S(4-FPh)	ND	3.5±0.2 (*11*)	6.1±0.3 (*18*)	>55±2	ND
**23j**	Et	2,6-Cl_2_	CH_2_S(4-MeOPh)	ND	3.2±0.2 (*15*)	4.1±0.4 (*19*)	>53±3	ND
**23k**	*i*-Pr	2,6-Cl_2_	CH_2_SMe	ND	27±2 (*96*)	>64±3	>64±4	ND
**23l**	*i*-Pr	2,6-Cl_2_	(CH_2_)_2_SMe	ND	8.0±0.4 (*18*)	>62±3	>62±4	ND
**23m**	*i*-Pr	2,6-Cl_2_	CH_2_SPh	ND	10±0.5 (*23*)	>55±2	>55±3	ND
**23n**	*i*-Pr	2,6-Cl_2_	CH_2_S(4-FPh)	ND	4.6±0.2 (*6*)	16±0.6 (*19*)	>53±3	ND
**23o**	*i*-Pr	2,6-Cl_2_	CH_2_S(4-MeOPh)	ND	4.5±0.2 (*8*)	8±0.4 (*14*)	>52±3	ND
**24a**	Me	3,5-Me_2_	CH_2_SMe	ND	12±0.7 (*6*)	>74±4	64±3 (*35*)	ND
**24b**	Me	3,5-Me_2_	(CH_2_)_2_SMe	ND	2.3±0.1 (*12*)	57±3 (*273*)	58±2 (*278*)	ND
**24c**	Me	3,5-Me_2_	CH_2_SPh	ND	10±0.5 (*14*)	28±2 (*38*)	>65±3	ND
**24d**	Me	3,5-Me_2_	CH_2_S(4-FPh)	ND	>62±3	31±2 (*10*)	>62±4	ND
**24e**	Me	3,5-Me_2_	CH_2_S(4-MeOPh)	ND	>61±2	>61±3	>61±3	ND
**24f**	Et	3,5-Me_2_	CH_2_SMe	ND	25±2 (*51*)	>75±3	>75±4	ND
**24g**	Et	3,5-Me_2_	(CH_2_)_2_SMe	ND	3±0.1 (*17*)	>72±4	>72±4	ND
**24h**	Et	3,5-Me_2_	CH_2_SPh	ND	4±0.2 (*10*)	18±1 (*46*)	>63±3	ND
**24i**	Et	3,5-Me_2_	CH_2_S(4-FPh)	ND	26±1 (*37*)	27±1 (*37*)	>60±3	ND
**24j**	Et	3,5-Me_2_	CH_2_S(4-MeOPh)	ND	>59±3	16±1 (*22*)	>59±3	ND
**24k**	*i*-Pr	3,5-Me_2_	CH_2_SMe	ND	1±0.05 (*12*)	>72±4	7±0.4 (*63*)	ND
**24l**	*i*-Pr	3,5-Me_2_	(CH_2_)_2_SMe	0.054±0.002 (*2*)	0.52±0.02(*19*)	1±0.05(*39*)	6.2±0.3 (*230*)	1.25±0.05 (*46*)
**24m**	*i*-Pr	3,5-Me_2_	CH_2_SPh	0.095±0.005 (*2*)	0.7±0.03(*15*)	1.3±0.05 (*26*)	7.3±0.4 (*149*)	1.35±0.054 (*28*)
**24n**	*i*-Pr	3,5-Me_2_	CH_2_S(4-FPh)	ND	1±0.04 (*18*)	2.3±0.1 (*40*)	9.5±0.6 (*170*)	ND
**24o**	*i*-Pr	3,5-Me_2_	CH_2_S(4-MeOPh)	ND	2.4±0.1 (*29*)	2.6±0.2 (*32*)	9.9±0.5 (*121*)	ND
**11** ^13^	Me	2,6-Cl_2_	CH_2_CH_2_(4-MeOPh)	0.00022±0.00003 (*2*)	2.3±0.2 (*16428*)	6.9±0.4 (*49286*)	0.87±0.05 (*6214*)	0.95±0.08(*6787*)
**12** ^14^	Me	2,6-F_2_	CH_2_CH_2_(4-MeOPh)	0.035±0.002 (*7*)	6.3±0.3 (*1286*)	8.9±0.5 (*1735*)	4.1±0.2 (*837*)	1.2±0.1(*245*)
NVP				>7	>7	>7	5.6±0.3 (*47*)	>7
EFV				0.63±0.03 (*90*)	0.044±0.003 (*6*)	1.6±0.1 (*224*)	0.39±0.02 (*56*)	0.40±0.016 (*57*)
Dapivirine				0.006±0.0003 (*10*)	0.012±0.0008 (*20*)	0.33±0.02 (*550*)	0.003±0.0001 (*5*)	0.054±0.002 (*90*)

a Values are means ± SD determined from at least two independent experiments, each one done in triplicate.

b Effective concentration 50, concentration needed to inhibit by 50% the HIV-1-induced cytopathic effect, evaluated with the MTT method in MT-4 cells.

c Fold resistance: ratio of EC_50_ value against drug-resistant strain and EC_50_ of the WT NL4-3 strain (Table 1).

d Clinical isolate resistant to NRTIs (lamivudine, emtricitabine) and NNRTIs (NVP, EFV, delavirdine).

e ND, not determined.

f Higher concentration could not be tested due to precipitation of compounds in the culture medium.

As shown in Table 2, compounds **21**–**24** mostly retained the SAR patterns regarding the effects on the antiviral activity of the substitution profile at C2, C5, and C6 positions observed against WT HIV-1 (Table 1). The MTM-*S*-DABOs across all four series (**21a**,**f**,**k**, **22a**,**f**,**k**, **23a**,**f**,**k**, and **24a**,**f**,**k**) displayed weak activity in the micromolar range against all tested mutant strains, yet demonstrating WT-like SAR trends, with two notable exceptions. The 2,6-Cl_2_-benzyl MTM-*S*-DABOs (**23a**,**f**,**k**), contrary to what observed against WT HIV-1, exhibited HEPT-like behavior with the antiviral activity slightly increasing in the order Me < Et < *i*-Pr at C5 position, while remaining extremely weak against all mutants (Table 2). In the 3,5-Me_2_-benzyl series (**24a**,**f**,**k**), the preferred profile of C5 substitution versus the entire panel of mutants followed the order Et < Me < *i*-Pr, slightly differing from the trend Me < Et < *i*-Pr registered against WT HIV-1 (Table 2). Similarly, the MTE-*S*-DABOs (**21b**,**g**,**l**, **22b**,**g**,**l**, **23b**,**g**,**l**, and **24b**,**g**,**l**) mostly retained the SAR observed with WT HIV-1. The Me and *i*-Pr substitutions at C5 position of the pyrimidinone core remained the most effective against all evaluated mutant strains in the case of 2,6-F_2_-benzyl (**21b**,**g**,**l**) and 3,5-Me_2_-benzyl (**24b**,**g**,**l**) derivatives, respectively. In contrast, the 2-Cl-6-F- (**22b**,**g**,**l**) and 2,6-Cl_2_-benzyl-substituted (**23b**,**g**,**l**) series showed an atypical pattern requiring an ethyl moiety at C5 (Et > Me > *i*-Pr) for the maximal activity against all mutants (Table 2). Among the PTM-*S*-DABOs, the 6-(2,6-dihalobenzyl)-2-thiothymines (**21c**–**e**, **22c**–**e**, and **23c**–**e**) and the 6-(3,5-Me_2_-benzyl)-5-*i*-Pr-2-thiouracil **24m** showed the strongest activity against all mutants, aligning with the results observed with WT HIV-1. For any substitution at the *para* phenyl ring position the three series of 2,6-dihalobenzyl PTM-*S*-DABOs (**21c**–**e**,**h**–**j**,**m-o**, **22c**–**e**,**h**–**j**,**m-o**, and **23c**–**e**,**h**–**j**,**m**–**o**) showed the following trend of antiviral potency against all mutants Me > Et > *i*-Pr, while among the 3,5-Me_2_benzyl PTM derivatives (**24c**–**e**,**h**–**j**,**m**–**o**) the SAR trend about the C5 substitution was the opposite (*i*-Pr > Et > Me), but identical to what observed against WT HIV-1 (Table 2). Irrespective of the substitution at C5 position, among the 2,6-dihalobenzyl PTM-*S*-DABOs the trend of antiviral potency against all variants, including IRLL98 and the double mutant K103N+Y181C, was always *p*MeOPhSCH_2_ > *p*FPhSCH_2_ > PhSCH_2_, while among 3,5-Me_2_benzyl analogues (**24c**–**e**,**h**–**j**,**m**–**o**) was reverted to PhSCH_2_ > *p*FPhSCH_2_ > *p*MeOPhSCH_2_.

Among all tested derivatives, the *p*-MeO- and, to a lesser extent, *p*-F-substituted PTM-*S*-DABOs belonging to the 6-(2,6-dihalobenzyl)-2-thiothymine series (**21d**,**e**, **22d**,**e**, and **23d**,**e**), the 5-isopropyl-3,5-Me_2_-benzyl MTE derivative **24l**, and its unsubstituted PTM analogue **24m** had the highest antiviral potencies toward the entire panel of HIV-1 mutants and isolates, outperforming NVP against all variants, except K103N where **24l** and **24m** displayed comparable activity. Although always less active than EFV against Y181C (EC_50_ = 0.32–1.2 μM vs 0.044 μM), these compounds were all superior to EFV against the clinical isolate IRLL98 (EC_50_ = 0.003-0.095 μM vs 0.63 μM for EFV), comparable against Y188L (EC_50_ = 0.85-5.3 μM vs 1.6 μM for EFV) and, with the exception of **24l** and **24m**, markedly more potent against both K103N (EC_50_ = 0.008-0.095 μM vs 0.39 μM for EFV) and the double mutant K103N+Y181C (EC_50_ = 0.042-0.25 μM vs 0.40 μM for EFV). Strikingly, the two most active derivatives, the 5-methyl-6-(2,6-F_2_-benzyl) *p*-MeO PTM-*S*-DABO **21e** and its 2,6-Cl_2_-benzyl analogues **23e**, with the only exception of IRLL98 where **21e** outperformed **12**, but **23e** was worse than **11**, consistently showed stronger inhibition across all HIV-1 variants than their phenethyl-*S*-DABO counterparts **11** and **12** suggesting a superiority of the PTM-*S*-DABO over the phenethyl-*S*-DABO in enhancing the resistance tolerance. Additionally, **21e** and **23e**, despite weaker than dapivirine against Y181C and Y188L, were substantially comparable against K103N and the double mutant K103N+Y181C, and slightly more active against the clinical isolate IRLL98.

### Enzyme Inhibitory Activity on HIV-1 WT and NNRTI-Resistant Mutated RT

The MTM-, MTE-, and PTM-*S*-DABOs **21**–**24**, along with the reference compounds NVP, EFV, dapivirine, and phenylethyl-*S*-DABOs **11** ^13^ and **12**,^14^ were also evaluated against recombinant WT HIV-1 RT and against a panel of NNRTI-resistant RT variants (K103N, Y181I, and L100I). The concentration required to achieve 50% inhibition (ID_50_), and the corresponding fold-resistance values are reported in Table 3.

**Table 3:**
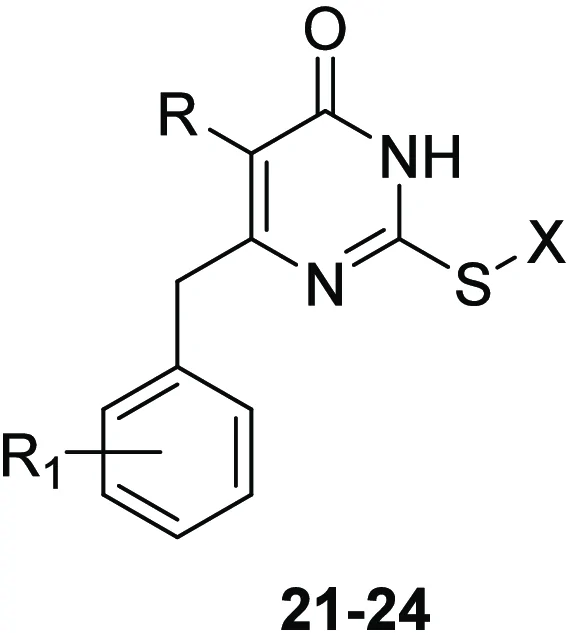
Inhibitory Activity of 21-24 against HIV-1 WT and NNRTI-Resistant rRTs^a^

				ID_50_,^b^ μM (*fold resistance*)^c^			
Compd	R	R_1_	X	WT RT	K103N	Y181I	L100I
**21a**	Me	2,6-F_2_	CH_2_SMe	0.105±0.005	>20^d^	>40	13.6±0.6 (129)
**21b**	Me	2,6-F_2_	(CH_2_)_2_SMe	0.008±0.0004	2.5±0.1 (*312*)	>40	3.3±0.1 (*412*)
**21c**	Me	2,6-F_2_	CH_2_SPh	0.021±0.001	0.45±0.02 (*21*)	9.2±0.4 (*438*)	1.5±0.08 (*71*)
**21d**	Me	2,6-F_2_	CH_2_S(4-FPh)	0.006±0.0003	0.09±0.005 (*15*)	5.1±0.3 (*850*)	0.68±0.03 (*113*)
**21e**	Me	2,6-F_2_	CH_2_S(4-MeOPh)	0.002±0.0001	0.044±0.002 (*22*)	4.3±0.2 (*2150*)	0.55±0.03 (*275*)
**21f**	Et	2,6-F_2_	CH_2_SMe	0.074±0.004	13.8±0.5 (187)	>40	5.2±0.3 (70)
**21g**	Et	2,6-F_2_	(CH_2_)_2_SMe	0.027±0.001	11.9±0.4 (*441*)	>40	2.5±0.1 (*93*)
**21h**	Et	2,6-F_2_	CH_2_SPh	0.079±0.004	16.8±0.5 (*213*)	12.8±0.5 (*162*)	2.1± 0.09 (*27*)
**21i**	Et	2,6-F_2_	CH_2_S(4-FPh)	0.081±0.005	7.5±0.3 (*92*)	13.4±0.6 (*166*)	1.7±0.07 (*21*)
**21j**	Et	2,6-F_2_	CH_2_S(4-MeOPh)	0.071±0.004	12.6±0.5 (*177*)	>40	1.3±0.05 (*18*)
**21k**	*i*-Pr	2,6-F_2_	CH_2_SMe	0.043±0.002	5.3±0.3 (124)	>40	4.2±0.2 (97)
**21l**	*i*-Pr	2,6-F_2_	(CH_2_)_2_SMe	0.095±0.005	>20	>40	4.5±0.2 (*47*)
**21m**	*i*-Pr	2,6-F_2_	CH_2_SPh	0.105±0.005	>40	>40	4.1±0.2 (*39*)
**21n**	*i*-Pr	2,6-F_2_	CH_2_S(4-FPh)	0.145±0.007	>40	>40	4.2±0.2 (*29*)
**21o**	*i*-Pr	2,6-F_2_	CH_2_S(4-MeOPh)	0.185±0.009	>40	>40	8.5±0.4 (*46*)
**22a**	Me	2-Cl-6-F	CH_2_SMe	0.269±0.01	>40	>40	6.7±0.3 (*25*)
**22b**	Me	2-Cl-6-F	(CH_2_)_2_SMe	0.028±0.001	18.2±0.8 (*650*)	>40	3.3±0.1 (*118*)
**22c**	Me	2-Cl-6-F	CH_2_SPh	0.031±0.001	8.4±0.4 (*271*)	10.3±0.5 (*332*)	2.6±0.1 (*84*)
**22d**	Me	2-Cl-6-F	CH_2_S(4-FPh)	0.011±0.0004	0.635±0.03 (*58*)	6.5±0.3 (*591*)	2.4 ± 0.1(*218*)
**22e**	Me	2-Cl-6-F	CH_2_S(4-MeOPh)	0.006±0.0003	0.078±0.003 (*13*)	2.9±0.1 (*483*)	1.2±0.05 (*200*)
**22f**	Et	2-Cl-6-F	CH_2_SMe	0.145±0.006	>40	>40	>40
**22g**	Et	2-Cl-6-F	(CH_2_)_2_SMe	0.079±0.004	11.9±0.6 (*151*)	>40	2.6±0.1 (*33*)
**22h**	Et	2-Cl-6-F	CH_2_SPh	0.095±0.005	16.7±0.7 (*176*)	12.8±0.5 (*135*)	2.1±0.1 (*22*)
**22i**	Et	2-Cl-6-F	CH_2_S(4-FPh)	0.081±0.004	7.4±0.3 (*91*)	13.4±0.6 (*165*)	1.6±0.06 (*20*)
**22j**	Et	2-Cl-6-F	CH_2_S(4-MeOPh)	0.071±0.004	12.6±0.6 (*177*)	>40	1.3±0.05 (*18*)
**22k**	*i*-Pr	2-Cl-6-F	CH_2_SMe	0.075±0.004	>40	>40	>20
**22l**	*i*-Pr	2-Cl-6-F	(CH_2_)_2_SMe	0.125±0.005	>20	>40	5.5±0.2 (*44*)
**22m**	*i*-Pr	2-Cl-6-F	CH_2_SPh	0.119±0.005	>40	>40	4.2±0.2 (*35*)
**22n**	*i*-Pr	2-Cl-6-F	CH_2_S(4-FPh)	0.169±0.007	>40	>40	4.2±0.2 (*25*)
**22o**	*i*-Pr	2-Cl-6-F	CH_2_S(4-MeOPh)	0.397±0.02	>40	>40	13.5±0.7 (*34*)
**23a**	Me	2,6-Cl_2_	CH_2_SMe	0.390±0.02	>40	>40	>40
**23b**	Me	2,6-Cl_2_	(CH_2_)_2_SMe	0.093±0.005	>40	>40	1.7±0.07 (*18*)
**23c**	Me	2,6-Cl_2_	CH_2_SPh	0.042±0.002	0.965±0.05 (*23*)	7.8±0.3 (*186*)	2.3±0.1 (*55*)
**23d**	Me	2,6-Cl_2_	CH_2_S(4-FPh)	0.010±0.002	0.445±0.02 (*44*)	4.3±0.2 (*430*)	1.8±0.09 (*180*)
**23e**	Me	2,6-Cl_2_	CH_2_S(4-MeOPh)	0.005±0.0002	0.045±0.002 (*9*)	1.4±0.06 (*280*)	1.1±0.05 (*217*)
**23f**	Et	2,6-Cl_2_	CH_2_SMe	0.52±0.03	>40	>40	>40
**23g**	Et	2,6-Cl_2_	(CH_2_)_2_SMe	0.65±0.03	>40	>40	>40
**23h**	Et	2,6-Cl_2_	CH_2_SPh	0.36±0.002	>40	>40	5.7±0.2 (*16*)
**23i**	Et	2,6-Cl_2_	CH_2_S(4-FPh)	0.26±0.01	>40	>40	>40±1.9
**23j**	Et	2,6-Cl_2_	CH_2_S(4-MeOPh)	0.18±0.009	17.9±1 (*99*)	>40	3±0.1 (*17*)
**23k**	*i*-Pr	2,6-Cl_2_	CH_2_SMe	0.60±0.03	>40	>40	>40
**23l**	*i*-Pr	2,6-Cl_2_	(CH_2_)_2_SMe	0.396±0.02	>20	>40	>30
**23m**	*i*-Pr	2,6-Cl_2_	CH_2_SPh	0.419±0.02	>40	>25	>40
**23n**	*i*-Pr	2,6-Cl_2_	CH_2_S(4-FPh)	0.925±0.05	>20	>40	15.7±0.8 (*17*)
**23o**	*i*-Pr	2,6-Cl_2_	CH_2_S(4-MeOPh)	0.438±0.02	>40	>40	12.2±0.6 (*28*)
**24a**	Me	3,5-Me_2_	CH_2_SMe	0.345±0.02	>40	5.5±0.2 (*16*)	5.5±0.3 (*16*)
**24b**	Me	3,5-Me_2_	(CH_2_)_2_SMe	0.05±0.002	>20	2.3±0.1 (*47*)	2.98±0.1 (*60*)
**24c**	Me	3,5-Me_2_	CH_2_SPh	0.098±0.005	>20	1.6±0.08 (*16*)	>40
**24d**	Me	3,5-Me_2_	CH_2_S(4-FPh)	0.106±0.005	>20	8.05±0.4 (*76*)	>40
**24e**	Me	3,5-Me_2_	CH_2_S(4-MeOPh)	0.129±0.006	>20	11.9±0.6 (*92*)	7.2±0.3 (*56*)
**24f**	Et	3,5-Me_2_	CH_2_SMe	0.048±0.002	>40	3.5±0.2 (*73*)	4.1±0.2 (*85*)
**24g**	Et	3,5-Me_2_	(CH_2_)_2_SMe	0.084±0.007	9.85±0.5 (*117*)	6.6±0.3 (*79*)	2.91±0.12 (*35*)
**24h**	Et	3,5-Me_2_	CH_2_SPh	0.019±0.0009	>20	7.5±0.4 (*390*)	1.9±0.1 (*100*)
**24i**	Et	3,5-Me_2_	CH_2_S(4-FPh)	0.020±0.001	>20	7.97±0.5 (*398*)	2.39±0.1 (*119*)
**24j**	Et	3,5-Me_2_	CH_2_S(4-MeOPh)	0.026±0.001	>20	8.74±0.5 (*336*)	1.65±0.08 (*63*)
**24k**	*i*-Pr	3,5-Me_2_	CH_2_SMe	0.026±0.001	7.3±0.4 (281)	19.4±1 (746)	1.8±0.09 (69)
**24l**	*i*-Pr	3,5-Me_2_	(CH_2_)_2_SMe	0.012±0.0006	5.61±0.3 (*467*)	2.13±0.19 (*177*)	0.44±0.02 (*37*)
**24m**	*i*-Pr	3,5-Me_2_	CH_2_SPh	0.014±0.0007	6.35 ± 0.3 (*794*)	2.21±0.09(*158*)	0.98±0.05 (*122*)
**24n**	*i*-Pr	3,5-Me_2_	CH_2_S(4-FPh)	0.037±0.002	>40	>25	>40
**24o**	*i*-Pr	3,5-Me_2_	CH_2_S(4-MeOPh)	0.058±0.003	>40	>25	>40
**11** ^13^	Me	2,6-Cl_2_	CH_2_CH_2_(4-MeOPh)	0.026±0.001	3.2± 0.2 (*123*)	1.5±0.07 (*57*)	ND^e^
**12** ^14^	Me	2,6-F_2_	CH_2_CH_2_(4-MeOPh)	0.029±0.001	3.5±0.2 (*127*)	>20	ND
NVP				0.4±0.02	6.8± 0.3 (*17*)	33±2 (*82*)	8.5±0.4 (*21*)
EFV				0.03±0.001	3 ±0.2 (*100*)	0.09±0.005 (*3*)	ND
Dapivirine				0.007±0.0003	0.1±0.04 (*14*)	>1	0.09±0.005 (*13*)

a Values are means ± SD determined from at least three experiments.

b Inhibitory dose 50, compound dose required to inhibit the HIV-1 rRT activity by 50%.

c Fold resistance: ratio of ID_50_mut/ID_50_WT values.

d Higher concentration could not be tested due to precipitation of compounds in the culture medium.

e ND, not determined.

Overall, the enzymatic data for **21**–**24** strongly correlated with the cellular results, supporting HIV-1 RT as their primary target and substantially confirming the SAR profiles regarding the substitutions at positions C2, C5, and C6 observed in cellular assays.

The only small deviation was observed in the 3,5-Me_2_-benzyl derivatives bearing a *p*-F/*p*-MeO substitution at the PTM side chain (**24d**,**e**,**i**,**j**,**n**,**o**), where an Et group at C5 instead of the *i*-Pr conferred the maximum inhibitory potency against both WT and mutated rRTs. However, consistently with the cellular data, in the 3,5-Me_2_-benzyl series **24** the best inhibitory potency against WT and mutated enzymes was obtained with the 5-isopropyl-3,5-Me_2_-benzyl MTE derivative **24l** and its unsubstituted PTM analogue **24m** (Table 3). Again, in line with cellular results, in the 2,6-dihalobenzyl series **21**–**23**, the introduction of a Me group at C5 and of a MeO group or, to lesser extent, a F atom at *para* position of the PTM side chain in C2 provided the highest inhibition against all tested RTs, leading to the two most potent derivatives **21e** and **23e**. Endowed with single-digit nanomolar inhibitory potency against WT RT and (sub)­micromolar potency toward mutated RTs, **21e** and **23e** resulted sensibly more potent than NVP, EFV, and the phenethyl-*S*-DABOs **11** ^13^ and **12** ^14^ versus all tested RTs, except Y181I in case of EFV. Moreover, both were slightly more active than dapivirine against WT and K103N RTs, comparable against Y181I, and weaker versus L100I mutated enzymes (Table 3).

### 2D-QSAR Analysis

To relate the structural features of these anti-HIV-1 agents to their activity, we performed a systematic QSAR analysis based on molecular field topology analysis (MFTA). In addition to conventional SAR, QSAR provides a quantitative mapping of descriptors across the entire molecular scaffold. By projecting these descriptors onto molecular supergraphs, MFTA can reveal both universal and mutation-dependent activity “hot-spots”. Based on the EC_50_ and ID_50_ data (Tables 1–3), we calculated the pID_50_ = −Log­(ID_50_) and pEC_50_ = −Log­(EC_50_) values. These data and the corresponding structures of organic compounds were entered into an SDF file using DataWarrior.^58^ Inactive compounds were included together with active analogues, whereas IRLL98 and K103N+Y181C were excluded due to low amount of data. The SDF files were used in the MFTA program, and the molecular supergraphs (Figure 3) were constructed for each compound series and each strain.^59^

**Figure 3:**
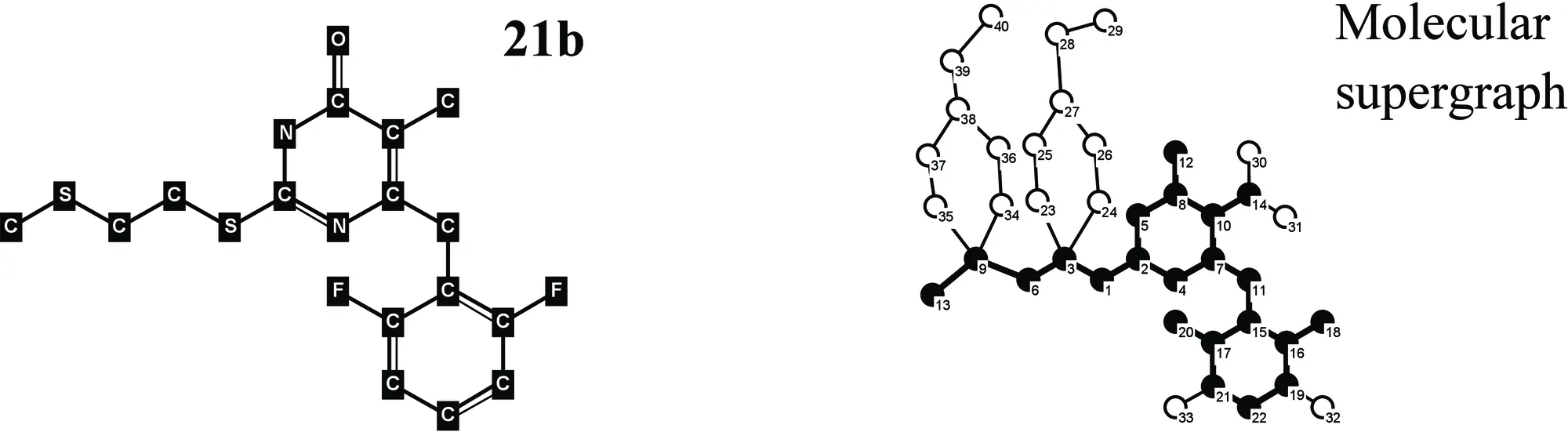
Compound **21b** and its superposition on a molecular supergraph.

For each molecular supergraph, the spatial distribution of molecular descriptors was calculated, including: (i) the effective atomic charge (Q), estimated through electronegativity equalization; (ii) the effective van der Waals radius of the first atomic environment (Re), reflecting the steric contributions of the central non-hydrogen atom and other bonded atoms; (iii) group lipophilicity (Lipo), accounting for the contributions of the central non-hydrogen atom and the hydrogen atoms bonded to it; and (iv) the hydrogen-bond donor (Hd) and acceptor (Ha) capacities of an atom.

In the resulting contour maps, red regions indicate favorable contributions of a given descriptor to the modeled property, whereas blue regions denote unfavorable descriptor localization. Color intensity reflects the magnitude of the contribution. Charge descriptors correspond exclusively to the analysis of σ-bond electronic density. Tables 4 and 5 show data obtained based on ID_50_ and EC_50_ values, respectively.

**Table 4:**
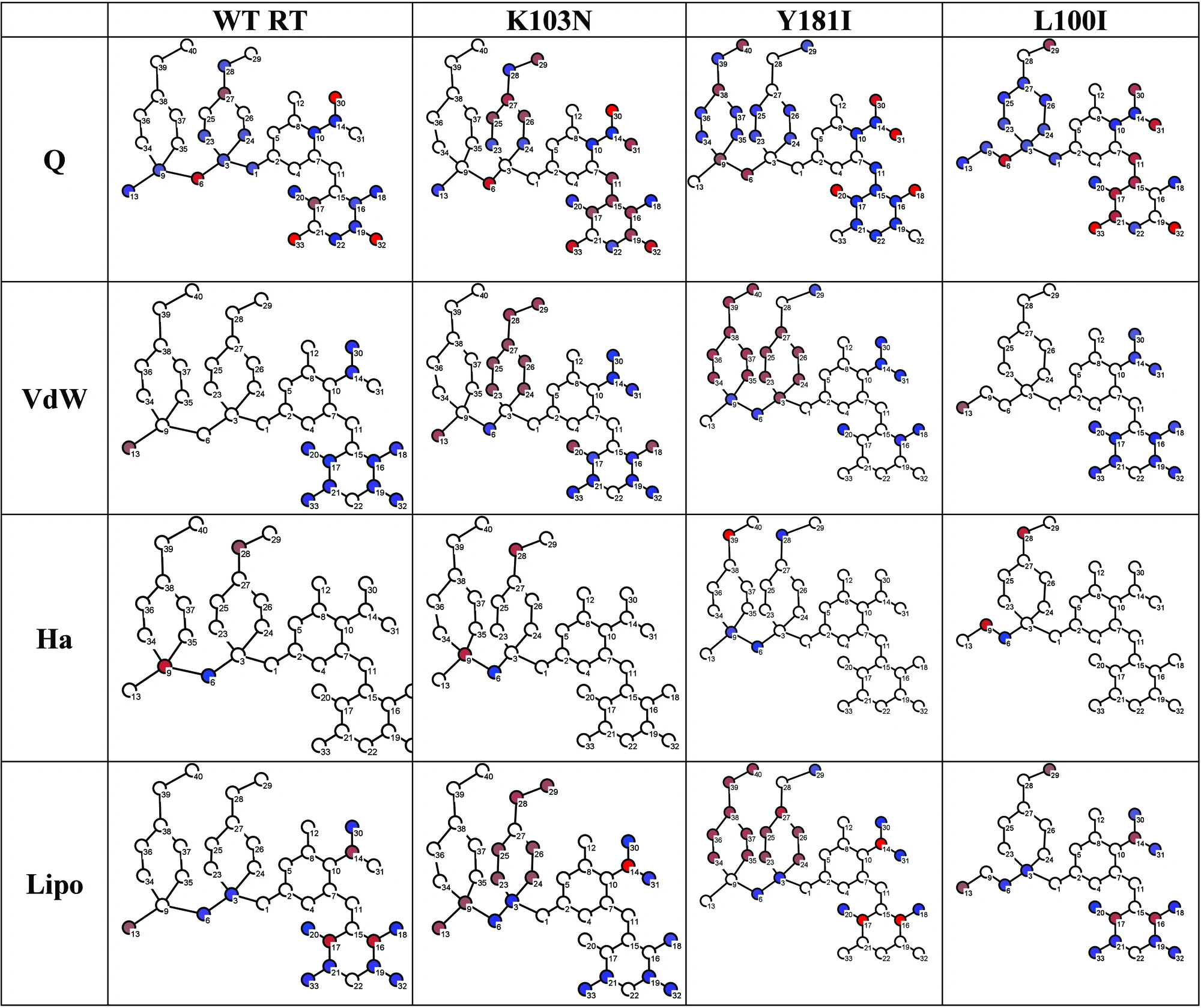
Molecular Supergraphs, Constructed Based on ID_50_ (*Fold Resistance*) Values

**Table 5:**
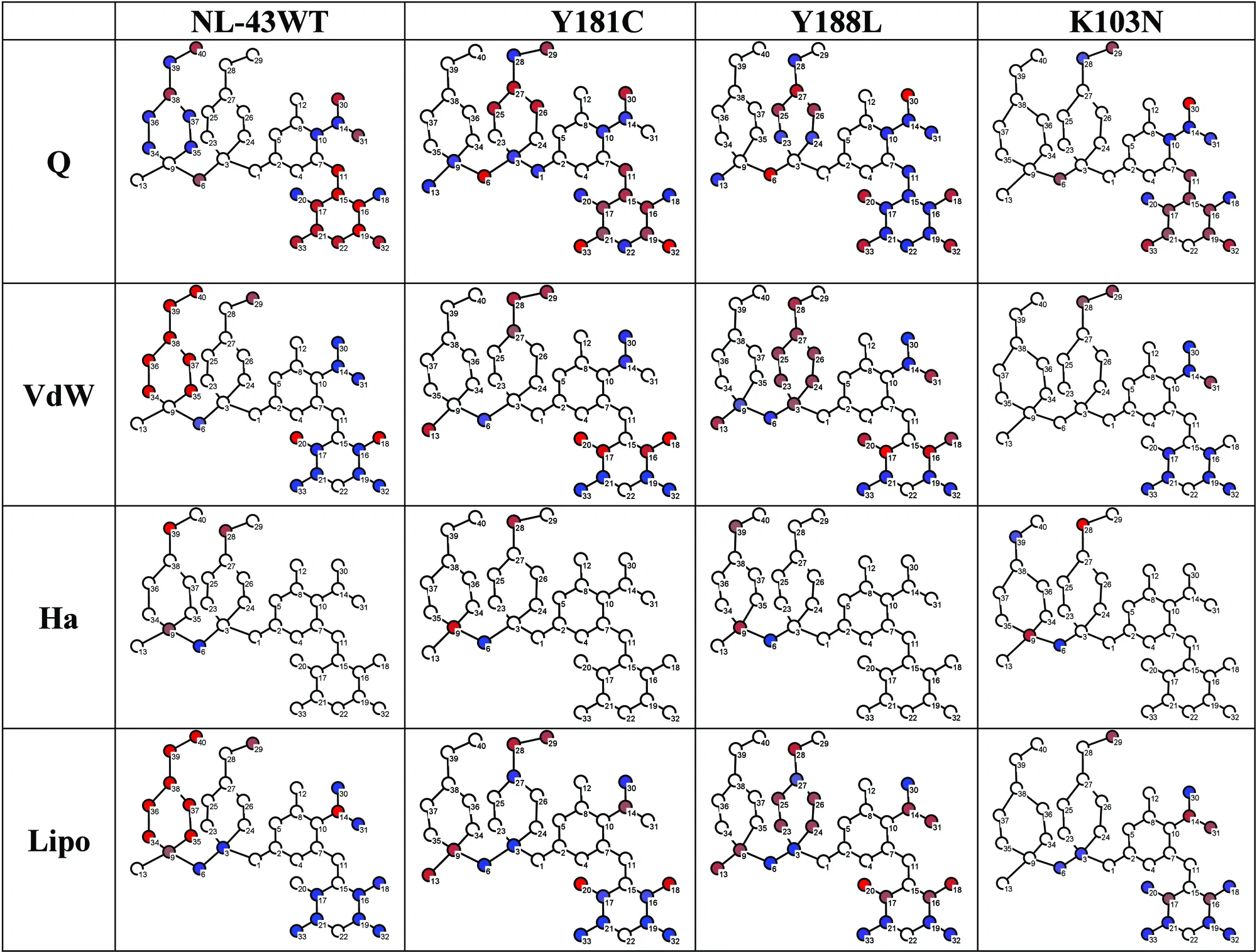
Molecule Supergraphs, Constructed Based on EC_50_ (*Fold Resistance*) Values

#### Charge Descriptor Analysis

The analysis was focused on the structural fragments positively contributing to the activity across all HIV-1 strains studied (Tables 1 and 2). The correspondence between the numbering scheme and the molecular positions is as follows: number 2 corresponds to C2 of the pyrimidinone ring, number 10 corresponds to C5, while number 7 to C6. Numbers 18 and 20 correspond to positions 2 and 6, respectively, of the benzene ring of the benzyl substituent at the C6 atom, whereas atoms 32 and 33 correspond to positions 3 and 5 of the benzene ring. In the ID_50_ models (Table 4), the most recurrent charge pattern involved positions 10-14-30: positions 10 and 14 correspond to a partially negative charge, while position 30 is preferable for a partially positive charge (electronic density donors). This corresponds to R = alkyl substituents from Tables 1 and 2. The same preferable positions can be seen for charge descriptors in EC_50_ data (Table 5). Another common position is position 6 with a partially positive charge, which is the same in the case of EC_50_. Positions 23 and 24, which are part of the benzene ring in the X substituent, exhibit preferable partial negative charges that are common across all enzyme variants in the ID_50_ data, indicating that these positions serve as favorable electronic density acceptors. EC_50_ data for charges (Table 5) shows a common pattern for the NL4-3 WT, Y181C, and K103N strains for the benzene ring in positions 21-17-15-16-19-22 (benzene ring of C6 benzyl substituent), where positive charges are preferred. This indicates that the benzene ring is a preferable electronic density donor here, which corresponds to preferable electronic density acceptors in positions 18 and 20. This corresponds to the 21-17-15-16-19-22 positions of the ring in the case of ID_50_ data (Table 4), and the picture looks somewhat different. Finally, positions 32 and 33 show a recurrent preference for increased electron density across all strains (except for Y181I), which corresponds to R_1_ = 3,5-Me_2_ and indicates a preference for electron-density donors.

#### VdW Descriptor Analysis

Positions 14-30 show preferable small VdW substituents for both ID_50_ and EC_50_, consistent with optimal activity for R = Me (Tables 1–3). Regions 33-21 and 19-33 preferentially accommodate sterically small groups across most viral strains, with the notable exception of Y181I in ID_50_ assays. This corresponds to R_1_ = 3,5-Me_2_ in Tables 1–3. Conversely, EC_50_ data indicates that the symmetric positions 20 and 18 indeed favor bulkier substituents for the NL-43WT, Y181C, and Y188L strains. This trend corresponds to R_1_ = 2,6-Cl_2_ substitution, suggesting that increased steric effects at these positions enhance antiviral potency.

#### Hydrogen Bond Analysis

Only hydrogen-bond acceptors (HBAs) were analyzed, since the variable structural regions did not contain hydrogen-bond donors. An HBA in position 28 is favorable for antiviral activity across all tested strains, except for Y181C and Y181I, as supported by both EC_50_ and ID_50_ datasets. This corresponds to the methoxy oxygen in the X = *p*-MeOPhSCH_2_ substituent at C2, highlighting the importance of a polar electron-rich site in this region for productive ligand-target interactions. Likewise, position 9 is favorable for HBAs against all strains based on EC_50_ values and for all, but the Y181I variant in ID_50_ assays. This position is associated with the sulfur atom in X = (CH_2_)_2_SMe; notably, oxygen substitution at this site seems tolerated, suggesting some flexibility in the nature of the HBA while preserving activity. Position 6 is unfavorable for HBAs for all the strains across all data.

#### Lipophilicity Analysis

Positions 33-21 and 19-32 are unfavorable for lipophilicity for all strains in both ID_50_ and EC_50_, with the sole exception of the Y181I strain in the ID_50_ dataset. Interestingly, position 14 is favorable for lipophilic substituents for all strains across all the data, consistent with the enhanced activity observed for R = Me. Position 29 also preferentially accommodates lipophilic substituents, particularly against WT NL4-3, Y181C, and K103N strains in EC_50_ data, and against K103N and L100I in ID_50_ data; this effect can be attributed to the methyl group of the *para*-methoxy substituent (X = *p*-MeOPhSCH_2_). On the other hand, position 30 is intolerant to lipophilic substitution across all datasets, indicating a sterically or polarity-constrained region.

Overall, differences between the ID_50_ and EC_50_-based descriptor distributions likely reflect the distinct experimental readouts and the additional biological factors influencing cellular antiviral activity. Nonetheless, the QSAR models identified consistent electronic, steric, hydrogen-bonding, and lipophilicity patterns that determine inhibitor activity across HIV-1 strains. Key positions (e.g., 10-14-30, 14-30, 28, 29) were shown to strongly influence potency and explain why certain substituents improve or weaken activity. The analysis revealed both common structural requirements for broad-spectrum activity and mutation-dependent preferences. The QSAR results provide descriptor-based guidelines for further optimization of the series.

Complementary 3D-QSAR, ligand- and structure-based docking, and COMBINE analyses further supported the observed SAR by showing, for the representative highly active derivative **21e**, accommodation of the 2,6-difluorobenzyl moiety within the aromatic-rich NNBS pocket, preservation of the key hydrogen bond between the pyrimidinone ring and Lys101, and extension of the C2 thioether side chain into an adjacent channel where additional van der Waals contacts contribute to ligand accommodation (see Figures S5–S8 and Table S10 in Supporting Information Part 2).

## Conclusion

Four series of MTM-, MTE-, and PTM-*S*-DABO derivatives (**21**–**24**) were evaluated against WT and drug-resistant HIV-1 variants. Cellular and enzymatic data confirmed the established *S*-DABOs SAR,^5–7,9–14,17,18,33,34,40–42^ highlighting the cooperative roles of the C6 benzyl, C5 alkyl, and C2 thioether substituents in antiviral activity (Figure 4).

**Figure 4:**
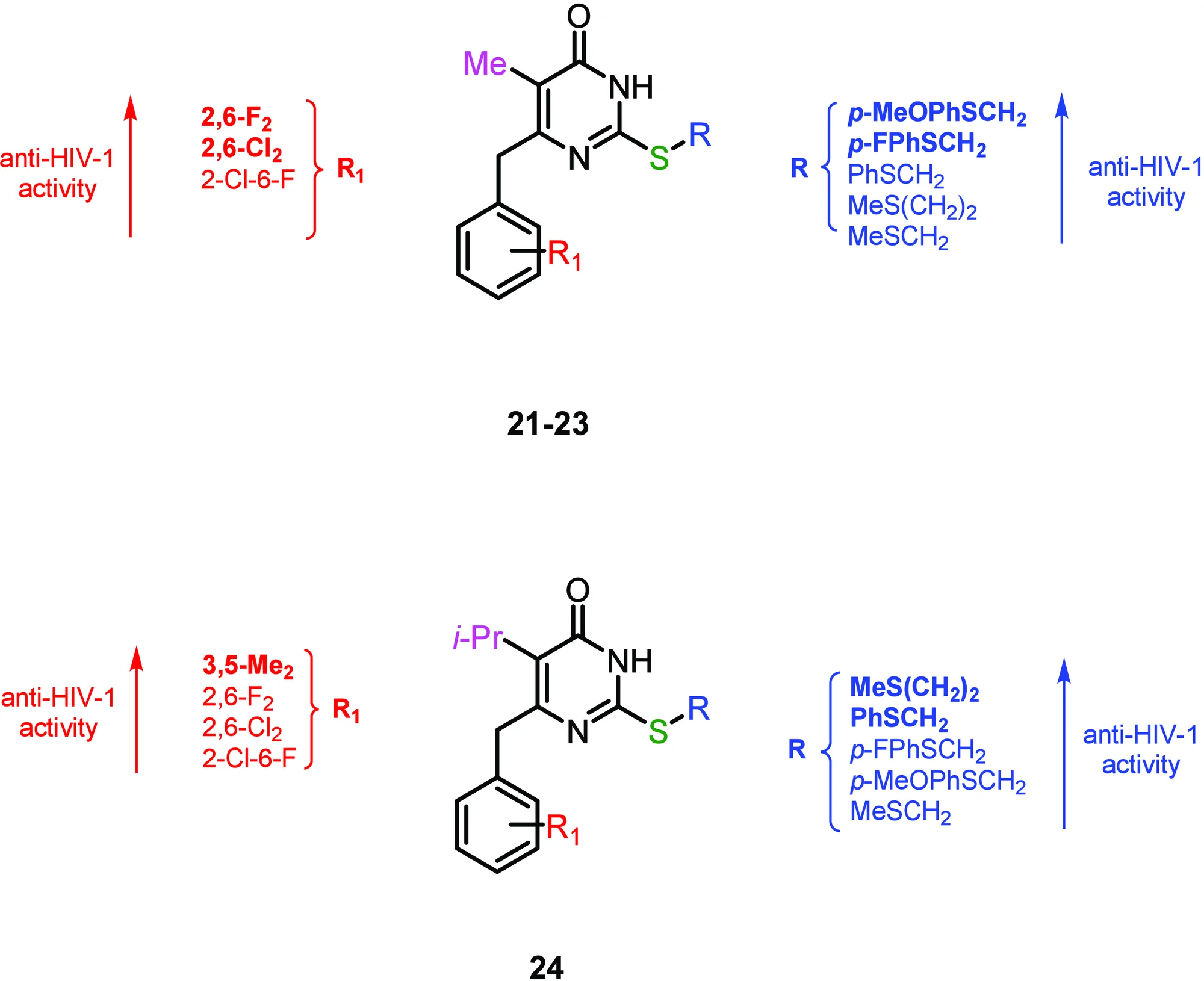
SAR graphical summary of MTM-, MTE-, and PTM-*S*-DABOs **21**–**24**.

Overall, *ortho*-dihalogenation at C6 was preferred over 3,5-Me_2_ substitution, with a general potency trend 2,6-F_2_ > 2,6-Cl_2_ > 2-Cl-6-F > 3,5-Me_2_, although specific inversions depend on C2 and C5 substitutions. In the 2,6-dihalobenzyl series, a C5 methyl group was generally optimal, whereas larger alkyl groups reduced activity against WT and HIV-1 mutants. However, selected derivatives showed an “HEPT-like” behavior, with C5 isopropyl becoming favorable, particularly in MTM-S-DABOs bearing 2,6-F_2_ or 2-Cl-6-F benzyl groups, and in 3,5-Me_2_-benzyl analogues irrespective of the C2 substituent. The C2 thioether side chains further modulated activity in a context-dependent manner. The best antiviral profile was observed for PTM-*S*-DABOs of the 2,6-dihalobenzyl thiothymine series (**21c**–**e**, **22c**–**e**, and **23c**–**e**), that always followed the activity order *p*MeOPhSCH_2_ > *p*FPhSCH_2_ > PhSCH_2_. Conversely, the 3,5-Me_2_-benzyl *S*-DABOs **24** showed the opposite trend, with PhSCH_2_ outperforming *para*-substituted PTM groups, suggesting a distinct binding mode. Among these compounds, the C5 isopropyl derivatives **24l** and **24m** were the most active (Figure 4).

The two most active derivatives, the 5-methyl-6-(2,6-F_2_-benzyl) *p*-MeO PTM-*S*-DABO **21e** and its 2,6-Cl_2_-benzyl analogue **23e**, showed subnanomolar-to-low-nanomolar activity against WT HIV-1 replication and rRT, combined with minimal cytotoxicity, excellent selectivity, and submicromolar-to-single digit nanomolar activity against drug-resistant HIV-1 variants and clinically relevant mutated rRT (Tables 1–3). Both compounds outperformed NVP and EFV across most clinically relevant strains, except Y181C, and displayed potency comparable to dapivirine against WT, K103N, IRLL98, and K103N+Y181C. They also consistently surpassed the phenethyl-*S*-DABO analogues **11** and **12** across nearly all mutant variants, supporting the phenylthiomethyl side chain as superior to the 2-phenylethyl group for improving resistance tolerance. Although the best 3,5-Me_2_-benzyl-substituted derivatives (**24l**,**m**) were less potent than **21e** and **23e** against WT, IRLL98, K103N, and K103N+Y181C strains, they retained comparable activity against Y181C and Y188L, with minimal fold resistance.

QSAR and molecular docking investigations further supported these SAR trends, linking broad-spectrum anti-HIV-1 activity and resistance tolerance to a defined combination of electronic, steric, hydrogen bonding, and lipophilic features. Electron-rich *para*-substituted phenylthiomethyl fragments, optimized C5 substituents, and properly positioned hydrogen-bond acceptors emerged as key determinants of productive RT inhibition.

In conclusion, this study identifies PTM-*S*-DABOs, particularly **21e** and **23e**, as promising next-generation NNRTI leads, defining a clear optimization map for the *S*-DABO chemotype and supporting the phenylthiomethyl thiothymine motif as a robust platform for developing NNRTIs with improved efficacy against clinically relevant HIV-1 strains.

## Experimental Section

### Chemistry

Melting points were determined on a Buchi 530 melting point apparatus and are uncorrected. ^1^H-NMR spectra were recorded at 400 MHz on a Bruker AC400 spectrometer, reporting chemical shifts in δ (ppm) units relative to the internal reference tetramethylsilane. Mass spectra were recorded on an API-TOF Mariner by Perspective Biosystems (Stratford, TX, USA), and samples were injected by a Harvard pump using a flow rate of 5-10 μL/min and infused into the electrospray system. Elemental analyses were obtained by a PE 2400 (PerkinElmer) analyzer and were used to determine the purity of the described compounds that is >95%. Analytical results are within ±0.40% of the theoretical values. All compounds were routinely checked by TLC and ^1^H-NMR. TLC was performed on aluminum-backed silica gel plates (Merck DC, Alufolien Kieselgel 60 F_254_) with spots visualized by UV light. Yields of reactions refer to purified products. All chemicals were purchased either from Aldrich Chimica, Milan (Italy), or from TCI Europe NV, Zwijndrecht (Belgium), and were of the highest purity. As a rule, samples prepared for physical and biological studies were dried in high vacuum over P_2_O_5_ for 20 h at temperatures ranging from 25 to 40 °C, depending on the sample’s melting point.

### General Procedure for the Preparation of 5-Alkyl-6-benzyl-2-{[(methylthio)­methyl]­thio}­pirimidin-4­(3*H*)-ones. Example: 6-(2,6-Dichlorobenzyl)-5-ethyl-2-{[(methylthio)­methyl]­thio}­pyrimidin-4­(3*H*)-one (**23f**, MC2569)

A solution of commercially available chloromethyl methyl sulfide (369 mg, 0.32 mL, 3.8 mmol) and 6-(2,6-dichlorobenzyl)-5-ethyl-2-thiouracil **25h** (0.6 g, 1.9 mmol) in anhydrous DMF (6 mL) was stirred overnight at room temperature. After completion the resulting mixture was quenched with cold water (60 mL), stirred for 1 h, and extracted with ethyl acetate (3×75 mL). The organic layer was washed in sequence with water (2×50 mL) and brine (3×25 mL), filtered through a TLC-grade silica gel pad, dried with sodium sulfate, and evaporated to dryness *in vacuo*. The residue was recrystallized from a mixture of acetonitrile and THF to give pure **23f** as a white solid. Yield, 53%; mp, 203-204°C. ^1^H-NMR (CDCl_3_) δ: 1.22 (t, 3H, CH_2_C*H*_3_), 2.01 (s, 3H, SC*H*_3_), 2.70 (q, 2H, C*H*_2_CH_3_), 3.94 (s, 2H, C*H*_2_Ar), 4.30 (s, 2H, SC*H*_2_S), 7.16-7.20 (m, 1H, C_4_-*H* Ar), 7.34-7.36 (m, 2H, C_3,5_-*H* Ar), 12.03 (br s, 1H, CON*H*) ppm. MS (ESI), m/z: 375 [M + H]^+^.

### General Procedure for the Preparation of 5-Alkyl-6-benzyl-2-{[2-(methylthio)­ethyl]­thio}­pirimidin-4­(3*H*)-ones. Example: 6-(3,5-Dimethylbenzyl)-5-isopropyl-2-{[2-(methylthio)­ethyl]­thio}­pyrimidin-4­(3*H*)-one (**24l**, MC2947)

6-(3,5-Dimethylbenzyl)-5-isopropyl-2-thiouracil **25l** (1.2 g, 4.2 mmol) was added to a stirred solution of 84.5% potassium hydroxide (277 mg, 4.2 mmol) in 20 mL of 95% ethanol. The resulting mixture was stirred for an additional hour at room temperature, treated with commercially available chloroethyl methyl sulfide (0.55 g, 0.49 mL, 5.0 mmol), and stirred overnight at room temperature. The next day, the mixture was further stirred at reflux under protection from atmospheric CO_2_ until the complete conversion of the starting 2-thiouracil derivative. Subsequently, the reaction mixture was chilled to room temperature, diluted with water, stirred for 1 h, and extracted with ethyl acetate (3×75 mL). The organic layer was washed successfully with water (2×50 mL) and brine (3×25 mL), filtered through a TLC-grade silica gel pad, dried over sodium sulfate, and evaporated to dryness *in vacuo*. The resulting residue was recrystallized from *n*-hexane to afford pure **24l** as a white solid. Yield, 35%, mp 106–107°C. ^1^H-NMR (CDCl_3_) δ 1.16-1.32 (d, 6H, CH­(C*H*_3_)_2_), 2.01 (s, 3H, SC*H*_3_), 2.16-2.32 (s, 6H, C_3,5_–C*H*_*3*_), 2.63-2.78 (t, 2H, SCH_2_C*H*_*2*_SCH_3_), 3.00-3.08 (m, 1H, C*H*(CH_3_)_2_), 3.16-3.25 (t, 2H, SC*H*_2_CH_*2*_SCH_3_), 3.81 (s, 2H, C*H*_2_Ar), 6.83 (s, 1H, C_4_-*H* Ar), 6.89 (s, 2H, C_2,6_-*H* Ar), 11.95 (br s, 1H, CONH). MS (ESI), m/z: 363 [M + H]^+^.

### General Procedure for the Preparation of 5-Alkyl-6-benzyl-2-{[(arylthio)­methyl]­thio}­pirimidin-4­(3*H*)-ones. Example: 6-(2-Chloro-6-fluorobenzyl)-5-methyl-2-{[(phenylthio)­methyl]­thio}­pyrimidin-4­(3*H*)-one (**22c**, MC2955)

6-(2-Chloro-6-fluorobenzyl)-2-thiothymine **25d** (1.2 g, 4.2 mmol) was added to a stirred solution of 84.5% potassium hydroxide (277 mg, 4.2 mmol) in 20 mL of 95% ethanol. The resulting mixture was stirred for an additional hour at room temperature, treated with commercially available chloromethyl phenyl sulfide (0.79 g, 0.67 mL, 5.0 mmol), and stirred overnight at room temperature. The next day, the mixture was stirred at reflux under protection from atmospheric CO_2_ until the complete conversion of the starting 2-thiothymine derivative. Subsequently, it was chilled to room temperature and diluted with water. The resulting precipitate was filtered off, washed with water and air-dried. Recrystallization of the crude product from 95% ethanol until the constant melting point furnished the target compound **22c**. Yield, 64%; mp 181-183°C; ^1^H-NMR (CDCl_3_) δ 2.09 (s, 3H, C*H*_3_), 4.00 (s, 2H, ArC*H*_2_), 4.24 (s, 2H, SC*H*_2_S), 6.89-6.95 (m, 1H, C_4_ *-H* Ar), 7.05-7.20 (m, 7H, C_3,5_ *-H* and C*H* Ar rings), 12.45 (br s, 1H, CON*H*). MS (ESI), m/z: 407 [M + H]^+^

Synthetic and analytical characterization data for the remaining final compounds of series **21**–**24**, including yields, melting points, ^1^H-NMR and ESI-MS analyses, are reported in the Supplementary Information Part 1.

### Biology. Anti-HIV-1 Activity in Lymphoid Cells

Biological activity of the compounds was tested in the lymphoid MT-4 cell line (received from the NIH AIDS Reagent Program) against the WT HIV-1 NL4-3 strain and five different HIV-1 strains, as described before.^60–62^ Briefly, MT-4 cells were infected with the appropriate HIV-1 strain (or mock-infected to determine cytotoxicity) in the presence of different drug concentrations. On day five postinfection, a tetrazolium-based colorimetric method (MTT method) was used to evaluate the number of viable cells. The IRLL98 HIV-1 strain contains the following mutations in the RT coding sequence: M41L, D67N, Y181C, M184 V, R211 K, and T215Y (conferring resistance to NRTIs) and mutations K101Q, Y181C, and G190A (conferring resistance to NNRTIs). The HIV-1 K103N, Y181C, Y188L, and the K103N+Y181C mutants were received from the Medical Research Council Centralized Facility for AIDS Reagents, Herfordshire, UK.

### RT Inhibition Assays

Chemicals:[^3^H]­dTTP (40 Ci/mmol) was purchased from Hartmann Analytic and unlabeled dNTPs from Promega. PerkinElmer was the supplier of GF/C filters. All other reagents were of analytical grade and purchased from Merck. Nucleic acid substrate: The homopolymer poly­(rA) was mixed at weight ratios in nucleotides of 10:1 to the oligomer oligo­(dT)­18in 20 mM Tris-HCl (pH 8.0) containing 20 mM KCl and 1 mM EDTA, heated at 65 °C for 5 min and then slowly cooled at room temperature. Enzymatic assay: The co-expression vectors pUC12N/p66­(His)/p51with the wild-type or the mutant forms of HIV-1 RT p66 were kindly provided by Dr. S. H. Hughes (NCI-Frederick Cancer Research and Development Center). Proteins were expressed in *Escherichia coli* and purified as described.^63^ RNA-dependent DNA polymerase activity was performed in a final volume of 15 μL containing reaction buffer (50 mM Tris-HCl pH 7.5, 1 mM DTT, 0.2 mg/mL BSA, 4% glycerol), 10% DMSO, 10 mM MgCl_2_, 0.5 μgof poly­(rA)/oligo­(dT)­10:1 (0.3 μM3′–OH ends), 10 μM [^3^H]-dTTP (1 Ci/mmol), and 2-4 nM RT. Reactions were incubated at 37 °C for 20 min. Then 10 μL aliquots were spotted on glass fiber filters GF/C that were immediately immersed in 5% ice-cold TCA. Filters were washed twice in 5% ice-cold TCA and once in ethanol for 5 min and dried, and acid-precipitable radioactivity was quantified by scintillation counting. Data obtained from monitoring the incorporation of radioactive [^3^H]­dTTP into poly­(rA)/oligo­(dT) in the presence of increasing doses of inhibitors were plotted using Graphpad Prism 5.0 software.

### Computational Investigations

See the separate Supporting Information Part 2 file.

## Supplementary Material





## Notes

Synthetic and analytical characterization of compounds **21**–**24**: yields, melting points, ^1^H NMR and ESI-MS data; physical and chemical data for the newly synthesized final compounds (Table S1); elemental analyses of the newly synthesized final compounds (Table S2) (PDF)
Computational investigations on compounds **21**–**24**: ligand-based 3D-QSAR/Py-CoMFA and structure-based molecular docking and Py-ComBinE/COMBINE approaches; computational modeling results and validation; experimental computational procedures; data and software availability; molecular structures, SMILES, pID50 values, and dataset assignment (Table S1); conformational analyses (Table S2); molecular alignment methods and scoring functions (Table S3); molecular properties used for template selection (Table S4); molecular parameters of compounds **21**–**24** (Table S5); Py-CoMFA models and alignment rules (Table S6); statistical performance of the selected 3D-QSAR model (Table S7); experimental, recalculated, cross-validated, and predicted pID50 values (Table S8); AutoDock Vina re-docking assessment (Table S9); optimized Py-ComBinE models (Table S10); experimental and predicted pID50 values for WT and mutant HIV-1 RTs (Table S11); 3D-QSAR model performance (Figure S1); steric and electrostatic activity-contribution maps (Figures S2 and S3); residue-interaction contributions from the Py-ComBinE model (Figure S4); representative binding modes of compound **21e** in WT, K103N, Y181I, and L100I HIV-1 RTs (Figures S5–S8) (PDF)

## Abbreviations

CC_50_: compound concentration toxic for 50% of cells
DABO: 3,4-dihydro-2-alkoxy-6-benzyl-4-oxo-pyrimidine
DACO: dihydro-aryl/alkylthio-cyclohexylmethyl-oxopyrimidine
DATNO: dihydro-alkylthio-naphthylmethyl-oxopyrimidine
DAPY: diarylpyrimidine
EC_50_: effective concentration 50, concentration needed to protect 50% of cells from the HIV-1 induced cytopathogenicity
EFV: efavirenz
F_2_-*N*H-DABO: 2-alkyl/arylamino-6-(2,6-difluorobenzyl)-4-(3*H*)-pyrimidinone
F_2_-*N*,*N*-DABO: 2-(*N*,*N*-disubstituted) amino-6-(2,6-difluorobenzyl)-4-(3*H*)-pyrimidinone
Ha: hydrogen-bond acceptor
HBDs: hydrogen bond donors
HBAs: hydrogen bond acceptors
Hd: hydrogen-bond donor
HEPT: 1-[(2-hydroxyethoxy)­methyl]-6-(phenylthio)­thymine
HIV-1: human immunodeficiency virus 1
ID_50_: 50% inhibiting dose
MFTA: molecular field topology analysis
MTE-*S*-DABOs: 6-(arylmethyl)-2-{[(methylthio)­ethyl]­thio}­pyrimidin-4­(3*H*)-ones
MTM-*S*-DABOs: 6-(arylmethyl)-2-{[(methylthio)­methyl]­thio}­pyrimidin-4­(3*H*)-ones
MTT: 3-(4,5-dimethylthiazol-2-yl)-2,5-diphenyltetrazolium bromide
NNBS: non-nucleoside binding site
NVP: nevirapine
PTM-*S*-DABOs: 6-(arylmethyl)-2-{[(phenylthio)­methyl]­thio}­pyrimidin-4­(3*H*)-ones
QSAR: quantitative structure–activity relationship
RT: reverse transcriptase
SI: selectivity index
