# Supplementary material for: Systematic Structure–Activity Relationship Investigation on Novel 5‑Alkyl-6-benzyl-2-{[(methylthio/phenylthio)alkyl]thio}-pyrimidin-4(3H)‑ones Endowed with Potent Anti-HIV‑1 Activity
Source: ACS Omega. 2026 Sep 16;11(38):58141–58. doi: 10.1021/acsomega.6c08020 (PMC13629370; doi:10.1021/acsomega.6c08020)
Supplement: Supplementary file 1 [file ao6c08020_si_001.pdf]

# Systematic Structure-Activity Relationship Investigation on Novel 5-Alkyl-6-Benzyl-2-{[(Methylthio/Phenylthio)Alkyl]Thio}-Pyrimidin-4(3*H*)-ones Endowed with Potent Anti-HIV-1 Activity

Gebremedhin Solomon Hailu[1,#], Egor Degtyarenko[2,#], Emanuele Fabbrizi[3,#], Anastasia Golovina[4], Andrey Voronkov[2], Andrea Mancini[3], Maxim B. Nawrozkij[2,4,*], Vladimir Chernyshov[5], Laura Bavagnoli[6], Elena Bianchi[6], Roberta Astolfi[3], Rino Ragno[3], José A. Esté[7], Emmanuele Crespan[6], Francesco Fiorentino[8,*], Roman Ivanov[4], Antonello Mai[3,*], Dante Rotili[1,9,*]

## SUPPORTING INFORMATION

**Content:**

Yields, Melting Points, $^1$H NMR, and ESI-MS Data of the New Compounds **21–24**. p. S2

**Table S1.** Physical and Chemical Data for the New Compounds **21–24**. p. S11

**Table S2.** Elemental Analyses for the Newly Synthesized Final Compounds **21-24**. p. S12

**Yields, Melting Points, $^1$H NMR, and ESI-MS Data of the New Compounds 21–24.**

*6-(2,6-Difluorobenzyl)-5-methyl-2-{[2-(methylthio)ethyl]thio}pyrimidin-4(3H)-one* ***(21b, MC3133).*** Yield, 74%, mp 176-178°C. $^1$H-NMR ($CDCl_3$) δ 2.09 (s, 3H, $SCH_3$), 2.18 (s, 3H, $CH_3$), 2.55 (t, 2H, $SCH_2CH_2SCH_3$), 3.11 (t, 2H, $SCH_2CH_2SCH_3$), 3.96 (s, 2H, $CH_2$Ar), 6.89-6.93 (m, 2H, $C_{3,5}$-*H* Ar), 7.21-7.25 (m, 1H, $C_4$-*H* Ar), 11.56 (br s, 1H, CON*H*). MS (ESI), m/z: 343 $[M + H]^+$.

*6-(2,6-Difluorobenzyl)-5-methyl-2-{[(phenylthio)methyl]thio}pyrimidin-4(3H)-one* ***(21c, MC3143).*** Yield, 89%, mp 206-208°C. $^1$H-NMR ($CDCl_3$) δ 2.18 (s, 3H, $CH_3$), 3.98 (s, 2H, $CH_2$Ar), 4.42 (s, 2H, $SCH_2$SPh), 6.89-6.93 (m, 2H, $C_{3,5}$-*H* Ar), 7.19-7.34 (m, 6H, $C_4$-*H* and C*H* Ar rings), 10.65 (br s, 1H, CON*H*). MS (ESI), m/z: 391 $[M + H]^+$.

*6-(2,6-Difluorobenzyl)-5-methyl-2-({[(4-fluorophenyl)thio]methyl}thio)pyrimidin-4(3H)-one* ***(21d, MC3157).*** Yield, 79%, mp 154-156°C. $^1$H-NMR ($CDCl_3$) δ 2.18 (s, 3H, $CH_3$), 3.97 (s, 2H, $CH_2$Ar), 4.35 (s, 2H, $SCH_2$S-), 6.90-7.02 (m, 4H, $C_{3,5}$-*H* and C*H* Ar rings), 7.24-7.38 (m, 3H, $C_4$-*H* and C*H* Ar rings), 10.59 (br s, 1H, CON*H*). MS (ESI), m/z: 409 $[M + H]^+$.

*6-(2,6-Difluorobenzyl)-2-({[(4-methoxyphenyl)thio]methyl}thio)-5-methylpyrimidin-4(3H)-one* ***(21e, MC3195).*** Yiled, 83%, mp 178-180°C. $^1$H-NMR ($CDCl_3$) δ 2.11 (s, 3H, $CH_3$), 3.75 (s, 3H, $OCH_3$), 4.10 (s, 2H, Ar$CH_2$), 4.20 (s, 2H, $SCH_2S$), 6.82-6.94 (m, 4H, $C_{3,5}$-*H* and C*H* Ar rings), 7.10-7.34 (m, 3H, $C_4$-*H* and C*H* Ar rings), 10.61 (br s, 1H, CON*H*). MS (ESI), m/z: 421 $[M + H]^+$.

*6-(2,6-Difluorobenzyl)-5-ethyl-2-{[2-(methylthio)ethyl]thio}pyrimidin-4(3H)-one* ***(21g, MC3130)***. Yield, 85%, mp 171-173°C. $^1$H-NMR ($CDCl_3$) δ 1.18 (t, 3H, $CH_2CH_3$), 2.08 (s, 3H, $SCH_3$), 2.53 (t, 2H, $SCH_2CH_2SCH_3$), 2.67 (q, 2H, $CH_2CH_3$), 3.08 (t, 2H, $SCH_2CH_2SCH_3$), 3.98 (s, 2H, $CH_2$Ar), 6.88-6.93 (m, 2H, $C_{3,5}$-*H* Ar), 7.19-7.27 (m, 1H, $C_4$-*H* Ar), 11.52 (br s, 1H, CON*H*). MS (ESI), m/z: 357 $[M + H]^+$.

*6-(2,6-Difluorobenzyl)-5-ethyl-2-{[(phenylthio)methyl]thio}pyrimidin-4(3H)-one* ***(21h, MC3144).*** Yield, 86%, mp 168-170°C. $^1$H-NMR ($CDCl_3$) δ 1.18 (t, 3H, $CH_2CH_3$), 2.67 (q, 2H, $CH_2CH_3$), 4.00 (s, 2H, $CH_2$Ar), 4.39 (s, 2H, $SCH_2$SPh), 6.89-6.93 (m, 2H, $C_{3,5}$-*H* Ar), 7.21-7.34 (m, 6H, $C_4$-*H* and C*H* Ar rings), 10.62 (br s, 1H, CON*H*). MS (ESI), m/z: 405 $[M + H]^+$.

*6-(2,6-Difluorobenzyl)-5-ethyl-2-({[(4-fluorophenyl)thio]methyl}thio)pyrimidin-4(3H)-one* ***(21i, MC3149)***. Yield, 73%, mp 137-139°C. $^1$H-NMR ($CDCl_3$) δ 1.18 (t, 3H, $CH_2CH_3$), 2.67 (q, 2H, $CH_2CH_3$), 3.99 (s, 2H, $CH_2$Ar), 4.32 (s, 2H, $SCH_2S$), 6.90-7.01 (m, 4H, $C_{3,5}$-*H* and C*H* Ar rings), 7.24-7.37 (m, 3H, $C_4$-*H* and C*H* Ar rings), 11.29 (br s, 1H, CON*H*). MS (ESI), m/z: 423 $[M + H]^+$.

*6-(2,6-Difluorobenzyl)-5-ethyl-2-({[(4-methoxyphenyl)thio]methyl}thio)pyrimidin-4(3H)-one* ***(21j, MC3194).*** Yield, 80%, mp 168-170°C. $^1$H-NMR ($CDCl_3$) δ 1.17 (t, 3H, $CH_2CH_3$), 2.61 (q, 2H, $CH_2CH_3$), 3.76 (s, 3H, $OCH_3$), 4.01 (s, 2H, $CH_2$Ar), 4.12 (s, 2H, $SCH_2S$), 6.85-6.95 (m, 4H, $C_{3,5}$-*H* and C*H* Ar rings), 7.18-7.31 (m, 3H, $C_4$-*H* and CH Ar), 11.28 (br s, 1H, CON*H*). MS (ESI), m/z: 435 $[M + H]^+$.

*6-(2,6-Difluorobenzyl)-5-isopropyl-2-{[2-(methylthio)ethyl]thio}pyrimidin-4(3H)-one* ***(21l, MC3137).*** Yield, 84%, mp 153-155°C; $^1$H-NMR ($CDCl_3$) δ 1.38 (d, 6H, $CH(CH_3)_2$), 2.09 (s, 3H, $SCH_3$), 2.52 (t, 2H, $SCH_2CH_2SCH_3$), 3.07 (t, 2H, $SCH_2CH_2SCH_3$), 3.20-3.27 (m, 1H, $CH(CH_3)_2$), 4.01 (s, 2H, $CH_2$Ar), 6.88-6.92 (m, 2H, $C_{3,5}$-*H* Ar), 7.19-7.26 (m, 1H, $C_4$-*H* Ar), 11.70 (br s, 1H, CON*H*). MS (ESI), m/z: 371 $[M + H]^+$.

*6-(2,6-Difluorobenzyl)-5-isopropyl-2-{[(phenylthio)methyl]thio}pyrimidin-4(3H)-one* ***(21m, MC3145).*** Yield, 80%, mp 179-181°C. $^1$H-NMR ($CDCl_3$) δ 1.37 (d, 6H, $CH(CH_3)_2$), 3.20-3.27 (m, 1H, $CH(CH_3)_2$), 4.03 (s, 2H, $CH_2$Ar), 4.35 (s, 2H, $SCH_2S$), 6.88-6.92 (m, 2H, $C_{3,5}$-*H* Ar), 7.20-7.34 (m, 6H, $C_4$-*H* and C*H* Ar rings), 11.60 (br s, 1H, CON*H*). MS (ESI), m/z: 419 $[M + H]^+$.

*6-(2,6-Difluorobenzyl)-2-({[(4-fluorophenyl)thio]methyl}thio)-5-isopropylpyrimidin-4(3H)-one* ***(21n, MC3147.)*** Yield, 72%, mp 147-149°C. $^1$H-NMR ($CDCl_3$) δ 1.38 (d, 6H, $CH(CH_3)_2$), 3.22-3.26 (m, 1H, $CH(CH_3)_2$), 4.02 (s, 2H, $CH_2$Ar), 4.29 (s, 2H, $SCH_2S$), 6.89-7.01 (m, 4H, $C_{3,5}$-*H* and C*H* Ar rings), 7.23-7.38 (m, 3H, $C_4$-*H* and C*H* Ar rings), 11.82 (br s, 1H, CON*H*). MS (ESI), m/z: 437 $[M + H]^+$.

*6-(2,6-Difluorobenzyl)-5-isopropyl-2-({[(4-methoxyphenyl)thio]methyl}thio)pyrimidin-4(3H)-one* ***(21o, MC3176).*** Yield, 77%, mp 121-123°C. $^1$H-NMR ($CDCl_3$) δ 1.38 (d, 6H, $CH(CH_3)_2$), 3.22-3.26 (m, 1H, $CH(CH_3)_2$), 3.81 (s, 3H, $OCH_3$), 4.00 (s, 2H, $CH_2$Ar), 4.25 (s, 2H, $SCH_2S$), 6.82-6.95 (m, 4H, $C_{3,5}$-*H* and C*H* Ar rings), 7.23-7.35 (m, 3H, $C_4$-*H* and C*H* Ar rings), 11.88 (br s, 1H, CON*H*). MS (ESI), m/z: 449 $[M + H]^+$.

*6-(2-Chloro-6-fluorobenzyl)-5-methyl-2-{[(methylthio)methyl]thio}pyrimidin-4(3H)-one* ***(22a, MC2576).*** Yield, 52%, mp 192-193°C; $^1$H-NMR ($CDCl_3$) δ: 2.04 (s, 3H, SC*H*$_3$), 2.21 (s, 3H, $C_5$-C*H*$_3$), 4.03 (s, 2H, C*H*$_2$Ar), 4.09 (s, 2H, SC*H*$_2$S), 7.02-7.04 (m, 1H, $C_4$-*H* Ar), 7.20-7.23 (m, 2H, $C_{3,5}$-*H* Ar), 12.34 (br s, 1H, CON*H*). MS (ESI), m/z: 345 $[M + H]^+$.

*6-(2-Chloro-6-fluorobenzyl)-5-methyl-2-{[2-(methylthio)ethyl]thio}pyrimidin-4(3H)-one* ***(22b, MC2954).*** Yield, 99%, mp 187-188°C; $^1$H-NMR ($CDCl_3$) δ 1.98 (s, 3H, SC*H*$_3$), 2.11 (s, 3H, C*H*$_3$), 2.36-2.40 (m, 2H, $CH_2$C*H*$_2$$SCH_3$), 2.92-2.97 (m, 2H, SC*H*$_2$$CH_2SCH_3$), 3.99 (s, 2H, ArC*H*$_2$), 6.92-6.99 (m, 1H, $C_4$-*H* Ar), 7.12-7.15 (m, 2H, $C_{3,5}$-*H* Ar), 12.25 (br s, 1H, CON*H*). MS (ESI), m/z: 359 $[M + H]^+$.

*6-(2-Chloro-6-fluorobenzyl)-2-({[(4-fluorophenyl)thio]methyl}thio)-5-methylpyrimidin-4(3H)-one* ***(22d, MC2956).*** Yield, 61%, mp 169-170°C; $^1$H-NMR ($CDCl_3$) δ 2.11 (s, 3H, C*H*$_3$), 4.01 (s, 2H, ArC*H*$_2$), 4.19 (s, 2H, SC*H*$_2$S), 6.88-6.98 (m, 3H, $C_4$-*H* and C*H* Ar rings), 7.11-7.27 (m, 4H, $C_{3,5}$-*H* and CH Ar rings), 12.22 (br s, 1H, CON*H*). MS (ESI), m/z: 425 $[M + H]^+$.

*6-(2-Chloro-6-fluorobenzyl)-2-({[(4-methoxyphenyl)thio]methyl}thio)-5-methylpyrimidin-4(3H)-one* ***(22e, MC2957)***. Yield, 61%, mp 210-212°C. $^1$H-NMR ($CDCl_3$) δ 2.11 (s, 3H, C*H*$_3$), 3.73 (s, 3H, OC*H*$_3$), 4.00 (s, 2H, ArC*H*$_2$), 4.15 (s, 2H, SC*H*$_2$S), 6.75 (d, 2H, $C_{3,5}$-*H* Ar), 6.94-7.00 (m, 1H, $C_4$-*H* Ar), 7.10-7.27 (m, 4H, C*H* Ar ring), 12.25 (br s, 1H, CON*H*).. MS (ESI), m/z: 437 $[M + H]^+$.

*6-(2-Chloro-6-fluorobenzyl)-5-ethyl-2-{[(methylthio)methyl]thio}pyrimidin-4(3H)-one* ***(22f, MC2585).*** Yield, 52%, mp 167-168°C. $^1$H-NMR ($CDCl_3$) δ: 1.21 (t, 3H, $CH_2$C*H*$_3$), 2.03 (s, 3H, SC*H*$_3$), 2.69 (q, 2H, C*H*$_2$$CH_3$), 3.99 (s, 2H, C*H*$_2$Ar), 4.12 (s, 2H, SC*H*$_2$S), 7.03 (m, 1H, $C_4$-*H* Ar), 7.20-7.23 (m, 2H, $C_{3,5}$ *H* Ar), 11.88 (br s, 1H, CON*H*). MS (ESI), m/z: 359 $[M + H]^+$.

*6-(2-Chloro-6-fluorobenzyl)-5-ethyl-2-{[2-(methylthio)ethyl]thio}pyrimidin-4(3H)-one* ***(22g, MC2958).*** Yield, 72%, mp 170-173°C. $^1$H-NMR ($CDCl_3$) δ 1.12 (t, $CH_2$C*H*$_3$), 1.97 (s, 3H, SC*H*$_3$), 2.33-2.38 (m, 2H, $CH_2$C*H*$_2$$SCH_3$), 2.60 (q, 2H, C*H*$_2$$CH_3$), 2.88-2.93 (m, 2H, SC*H*$_2$$CH_2SCH_3$), 4.02 (s, 2H, ArC*H*$_2$), 6.91-6.97 (m, 1H, $C_4$-*H* Ar), 7.11-7.15 (m, 2H, $C_{3,5}$-*H* Ar), 11.93 (br s, 1H, CON*H*). MS (ESI), m/z: 373 $[M + H]^+$.

*6-(2-Chloro-6-fluorobenzyl)-5-ethyl-2-{[(phenylthio)methyl]thio}pyrimidin-4(3H)-one* ***(22h, MC2959).*** Yield, 61%, mp 179-181°C. $^1$H-NMR ($CDCl_3$) δ 1.06-1.12 (m, 3H, $CH_2$C*H*$_3$), 2.54-2.62 (m, 2H, C*H*$_2$$CH_3$), 4.03-4.05 (s, 2H, ArC*H*$_2$), 4.20-4.21 (s, 2H, SC*H*$_2$S), 6.89-6.96 (m, 1H, $C_4$-*H* Ar), 7.06-7.19 (m, 7H, $C_{3,5}$-*H* and CH Ar rings), 12.14 (br s, 1H, CON*H*). MS (ESI), m/z: 421 $[M + H]^+$.

*6-(2-Chloro-6-fluorobenzyl)-5-ethyl-2-({[(4-fluorophenyl)thio]methyl}thio)pyrimidin-4(3H)-one* ***(22i, MC2960).*** Yield, 28%, mp 129-130°C. $^1$H-NMR ($CDCl_3$) δ 1.12 (t, 3H, $CH_2CH_3$), 2.61 (q, 2H, $CH_2CH_3$), 4.05 (s, 2H, Ar$CH_2$), 4.18 (s, 2H, S$CH_2$S), 6.85-7.00 (m, 3H, $C_4$-*H* and C*H* Ar rings), 7.14-7.27 (m, 4H, $C_{3,5}$-*H* and C*H* Ar rings), 11.48 (br s, 1H, CON*H*). MS (ESI), m/z: 439 $[M + H]^+$.

*6-(2-Chloro-6-fluorobenzyl)-5-ethyl-2-({[(4-methoxyphenyl)thio]methyl}thio)pyrimidin-4(3H)-one* ***(22j, MC2961).*** Yield, 58%, mp 181-183°C. $^1$H-NMR ($CDCl_3$) δ 1.07-1.13 (m, 3H, $CH_2CH_3$), 2.59 (q, 2H, $CH_2CH_3$), 3.71 (s, 3H, O$CH_3$), 4.03 (s, 2H, Ar$CH_2$), 4.10 (s, 2H, S$CH_2$S), 6.73 (d, 2H, $C_{3,5}$-*H* Ar), 6.92-6.98 (m, 1H, $C_4$-*H* Ar), 7.09-7.24 (m, 4H, C*H* Ar), 11.98 (br s, 1H, CON*H*). MS (ESI), m/z: 451 $[M + H]^+$.

*6-(2-Chloro-6-fluorobenzyl)-5-isopropyl-2-{[(methylthio)methyl]thio}pyrimidin-4(3H)-one* ***(22k, MC2586).*** Yield, 43%, mp 172-173°C. $^1$H-NMR ($CDCl_3$) δ: 1.41 (d, 6H, $CH(CH_3)_2$), 2.03 (s, 3H, S$CH_3$), 3.21-3.28 (m, 1H, $CH(CH_3)_2$), 3.95 (s, 2H, $CH_2$Ar), 4.16 (s, 2H, S$CH_2$S), 7.00-7.03 (m, 1H, $C_4$-*H* Ar), 7.20-7.23 (m, 2H, $C_{3,5}$-*H* Ar), 12.25 (br s, 1H, CON*H*). MS (ESI), m/z: 373 $[M + H]^+$.

*6-(2-Chloro-6-fluorobenzyl)-5-isopropyl-2-{[2-(methylthio)ethyl]thio}pyrimidin-4(3H)-one* ***(22l, MC2962).*** Yield, 76%, mp 165-166°C. $^1$H-NMR ($CDCl_3$) δ 1.32 (d, 6H, $CH(CH_3)_2$), 1.98 (s, 3H, S$CH_3$), 2.31-2.36 (m, 2H, $CH_2CH_2SCH_3$), 2.85-2.90 (m, 2H, $SCH_2CH_2SCH_3$), 3.15 (m, 1H, $CH(CH_3)_2$), 4.05 (d, 2H, Ar$CH_2$), 6.91-6.97 (m, 1H, $C_4$-*H* Ar), 7.08-7.14 (m, 2H, $C_{3,5}$*H* Ar), 12.15 (br s, 1H, CON*H*). MS (ESI), m/z: 387 $[M + H]^+$.

*6-(2-Chloro-6-fluorobenzyl)-5-isopropyl-2-{[(phenylthio)methyl]thio}pyrimidin-4(3H)-one* ***(22m, MC2963).*** Yield, 54%, mp 201-202°C. $^1$H-NMR ($CDCl_3$) δ 1.30 (d, 6H, $CH(CH_3)_2$), 3.15 (m, 1H, $CH(CH_3)_2$), 4.07 (s, 2H, Ar$CH_2$), 4.17 (s, 2H, S$CH_2$S), 6.90-6.96 (m, 1H, $C_4$-*H* Ar), 7.06-7.23 (m, 7H, $C_{3,5}$*H* and C*H* Ar rings), 12.25 (br s, 1H, CON*H*). MS (ESI), m/z: 435 $[M + H]^+$.

*6-(2-Chloro-6-fluorobenzyl)-2-({[(4-fluorophenyl)thio]methyl}thio)-5-isopropylpyrimidin-4(3H)-one* ***(22n, MC2964).*** Yield, 53%, mp 175-177°C. $^1$H-NMR ($CDCl_3$) δ 1.31 (d, 6H, $CH(CH_3)_2$), 3.15 (m, 1H, $CH(CH_3)_2$), 4.08 (s, 2H, Ar$CH_2$), 4.10 (s, 2H, S$CH_2$S), 6.87-6.97 (m, 3H, $C_4$-*H* and C*H* Ar rings), 7.08-7.16 (m, 2H, $C_{3,5}$-*H* Ar), 7.23-7.28 (m, 2H, C*H* Ar), 12.48 (br s, 1H, CON*H*). MS (ESI), m/z: 453 $[M + H]^+$.

*6-(2-Chloro-6-fluorobenzyl)-5-isopropyl-2-({[(4-methoxyphenyl)thio]methyl}thio)pyrimidin-4(3H)-one* ***(22o, MC2965).*** Yield, 54%, mp 125-127°C. $^1$H-NMR ($CDCl_3$) δ 1.31 (d, 6H, CH(C$H_3$)$_2$), 3.15 (m, 1H, C$H$($CH_3$)$_2$), 3.72 (s, 3H, OC$H_3$), 4.06 (s, 4H, ArC$H_2$ and SC$H_2$S), 6.74 (d, 2H, $C_{3,5}$-$H$ Ar), 6.92-6.98 (m, 1H, $C_4$-$H$ Ar), 7.10-7.25 (m, 4H, C$H$ Ar), 12.38 (br s, 1H, CON$H$). MS (ESI), m/z: 465 $[M + H]^+$.

*6-(2,6-Dichlorobenzyl)-5-methyl-2-{[(methylthio)methyl]thio}pyrimidin-4(3H)-one* ***(23a, MC2579)***. Yield, 53%, mp 228-229°C. $^1$H-NMR ($CDCl_3$) δ: 2.02 (s, 3H, SC$H_3$), 2.21 (s, 3H, $C_5$-C$H_3$), 3.99 (s, 2H, C$H_2$Ar), 4.25 (s, 2H, SC$H_2$S), 7.17-7.19 (m, 1H, $C_4$-$H$ Ar), 7.34-7.36 (m, 2H, $C_{3,5}$-$H$ Ar), 11.86 (br s, 1H, CON$H$). MS (ESI), m/z: 361 $[M + H]^+$.

*6-(2,6-Dichlorobenzyl)-5-methyl-2-{[2-(methylthio)ethyl]thio}pyrimidin-4(3H)-one* ***(23b, MC2966).*** Yield, 97%, mp 189-190°C. $^1$H-NMR ($CDCl_3$) δ 1.96 (s, 3H, SC$H_3$), 2.12 (s, 3H, C$H_3$), 2.29-2.34 (m, 2H, $CH_2$C$H_2$S$CH_3$), 2.87-2.92 (m, 2H, SC$H_2$$CH_2$S$CH_3$), 4.15 (s, 2H, ArC$H_2$), 7.06-7.12 (m, 1H, $C_4$-$H$ Ar), 7.27 (d, 2H, $C_{3,5}$-$H$ Ar), 11.96 (br s, 1H, CON$H$). MS (ESI), m/z: 375 $[M + H]^+$.

*6-(2,6-Dichlorobenzyl)-5-methyl-2-{[(phenylthio)methyl]thio}pyrimidin-4(3H)-one* ***(23c, MC2967).*** Yiled, 68%, mp 196-197°C. $^1$H-NMR ($CDCl_3$) δ 2.11 (s, 3H, C$H_3$), 4.18 (s, 2H, ArC$H_2$), 4.21 (s, 2H, SC$H_2$S), 7.06 (t, 1H, $C_4$-$H$ Ar), 7.13-7.27 (m, 7H, $C_{3,5}$-$H$ and C$H$ Ar rings), 11.82 (br s, 1H, CON$H$). MS (ESI), m/z: 423 $[M + H]^+$.

*6-(2,6-Dichlorobenzyl)-2-({[(4-fluorophenyl)thio]methyl}thio)-5-methylpyrimidin-4(3H)-one* ***(23d, MC2968).*** Yield, 70%, mp 187-188°C. $^1$H-NMR ($CDCl_3$) δ 2.11 (s, 3H, C$H_3$), 4.14 (s, 2H, ArC$H_2$), 4.17 (s, 2H, SC$H_2$S), 6.88-6.94 (m, 2H, $C_{3,5}$-$H$ Ar), 7.06-7.12 (m, 1H, $C_4$-$H$ Ar), 7.19-7.28 (m, 4H, C$H$ Ar), 11.95 (br s, 1H, CON$H$). MS (ESI), m/z: 441 $[M + H]^+$.

*6-(2,6-Dichlorobenzyl)-2-({[(4-methoxyphenyl)thio]methyl}thio)-5-methylpyrimidin-4(3H)-one* ***(23e, MC2969).*** Yield, 64%, mp 221-222°C. $^1$H-NMR ($CDCl_3$) δ 2.11 (s, 3H, C$H_3$), 3.73 (s, 3H, OC$H_3$), 4.10 (s, 2H, ArC$H_2$), 4.17 (s, 2H, C$H_2$), 6.75 (d, 2H, $C_{3,5}$-$H$ Ar), 7.09 (t, 3H, $C_4$-$H$ and CH Ar rings), 7.23-7.29 (m, 2H, C$H$ Ar), 11.89 (br s, 1H, CON$H$). MS (ESI), m/z: 453 $[M + H]^+$.

*6-(2,6-Dichlorobenzyl)-5-ethyl-2-{[2-(methylthio)ethyl]thio}pyrimidin-4(3H)-one* ***(23g, MX30).*** Yield, 35%, mp 203-205°C. $^1$H-NMR ($CCl_4$) δ 1.09-1.17 (m, 3H, $CH_2$C$H_3$), 1.94 (s, 3H, SC$H_3$), 2.15-2.25 (m, 2H, $CH_2$C$H_2$S$CH_3$), 2.51-2.66 (m, 2H, C$H_2$$CH_3$), 2.74-2.80 (m, 2H, SC$H_2$$CH_2$S$CH_3$), 4.18 (s, 2H, ArC$H_2$), 7.08 (t, 1H, $C_4$-$H$ Ar), 7.25 (d, 2H, $C_{3,5}$-$H$ Ar), 13.59 (br s, 1H, CON$H$). MS (ESI), m/z: 389 $[M + H]^+$.

*6-(2,6-Dichlorobenzyl)-5-ethyl-2-{[(phenylthio)methyl]thio}pyrimidin-4(3H)-one* ***(23h, MX31).*** Yield, 30%, mp 168-169°C. $^1$H-NMR ($CCl_4$) δ 1.02-1.07 (m, 3H, $CH_2CH_3$), 2.49-2.54 (m, 2H, $CH_2CH_3$), 4.18 (s, 2H, Ar$CH_2$), 4.30 (m, 2H, S$CH_2$S), 7.08-7.17 (m, 3H, $C_4$-*H* and C*H* Ar rings), 7.20-7.27 (m, 3H, $C_{3,5}$-*H* and C*H* Ar rings), 7.37-7.47 (m, 2H, C*H* Ar), 12.55 (br s, 1H, CON*H*). MS (ESI), m/z: 437 $[M + H]^+$.

*6-(2,6-Dichlorobenzyl)-5-ethyl-2-({[(4-fluorophenyl)thio]methyl}thio)pyrimidin-4(3H)-one* ***(23i, MX32).*** Yield, 31%, mp 165-166°C. $^1$H-NMR (DMF-$d_7$) δ 1.23-1.32 (m, 3H, $CH_2CH_3$), 2.49-2.63 (m, 2H, $CH_2CH_3$), 4.17-4.27 (s, 2H, Ar$CH_2$), 4.29-4.36 (s, 2H, S$CH_2$S), 7.03-7.15 (m, 2H, $C_{3,5}$-*H* Ar), 7.20-7.32 (m, 3H, $C_4$-*H* and C*H* Ar rings), 7.36-7.46 (m, 2H, C*H* Ar), 12.57 (br s, 1H, CON*H*). MS (ESI), m/z: 455 $[M + H]^+$.

*6-(2,6-Dichlorobenzyl)-5-ethyl-2-({[(4-methoxyphenyl)thio]methyl}thio)pyrimidin-4(3H)-one* ***(23j, MX33).*** Yield, 27%, mp 168-170°C. $^1$H-NMR ($CCl_4$) δ 1.01-1.06 (m, 3H, $CH_2CH_3$), 2.48-2.53 (m, 2H, $CH_2CH_3$), 3.68 (s, 3H, O$CH_3$), 4.16 (s, 4H, Ar$CH_2$ and S$CH_2$S), 6.82 (d, 2H, $C_{3,5}$-*H* Ar), 7.14 (d, 2H, C*H* Ar), 7.23-7.29 (m, 1H, $C_4$-*H* Ar), 7.42 (d, 2H, C*H* Ar), 12.47 (br s, 1H, CON*H*). MS (ESI), m/z: 467 $[M + H]^+$.

*6-(2,6-Dichlorobenzyl)-5-isopropyl-2-{[(methylthio)methyl]thio}pyrimidin-4(3H)-one* ***(23k, MC2572).*** Yield, 44%, mp 213-214°C. $^1$H-NMR (DMSO-$d_6$) δ: 1.31 (d, 6H, $CH(CH_3)_2$), 1.89 (s, 3H, S$CH_3$), 3.19-3.21 (m, 1H, $CH(CH_3)_2$), 3.89 (s, 2H, $CH_2$Ar), 4.25 (s, 2H, S$CH_2$S), 7.31-7.33 (m, 1H, $C_4$-*H* Ar), 7.44-7.47 (m, 2H, $C_{3,5}$-*H* Ar), 12.53 (br s, 1H, CON*H*). MS (ESI), m/z: 389 $[M + H]^+$.

*6-(2,6-Dichlorobenzyl)-5-isopropyl-2-{[2-(methylthio)ethyl]thio}pyrimidin-4(3H)-one* ***(23l, MC2970).*** Yield, 22%, mp 194-195°C. $^1$H-NMR ($CDCl_3$) δ 1.33-1.36 (m, 6H, $CH(CH_3)_2$), 1.96 (s, 3H, S$CH_3$), 2.25-2.30 (m, 2H, $CH_2CH_2SCH_3$), 2.79-2.83 (m, 2H, $SCH_2CH_2SCH_3$), 3.08-3.17 (m, 1H, $CH(CH_3)_2$), 4.23 (s, 2H, Ar$CH_2$), 7.06-7.12 (m, 1H, $C_4$-*H* Ar), 7.27 (d, 2H, $C_{3,5}$-*H* Ar), 12.38 (br s, 1H, CON*H*). MS (ESI), m/z: 403 $[M + H]^+$.

*6-(2,6-Dichlorobenzyl)-5-isopropyl-2-{[(phenylthio)methyl]thio}pyrimidin-4(3H)-one* ***(23m, MX54).*** Yield, 47%, mp 200-201°C. $^1$H-NMR ($CDCl_3$) δ 1.32 (d, 6H, $CH(CH_3)_2$), 3.05-3.14 (m, 1H, $CH(CH_3)_2$), 4.04 (s, 2H, S$CH_2$S), 4.23 (s, 2H, Ar$CH_2$), 7.00-7.23 (m, 8H, $C_{3,4,5}$-*H* and C*H* Ar rings), 13.56 (br s, 1H, CON*H*). MS (ESI), m/z: 451 $[M + H]^+$.

*6-(2,6-Dichlorobenzyl)-2-({[(4-fluorophenyl)thio]methyl}thio)-5-isopropylpyrimidin-4(3H)-one* ***(23n, MC2971).*** Yield, 26%, mp 198-200°C. $^1$H-NMR ($CDCl_3$) δ 1.33 (d, 6H, $CH(CH_3)_2$), 3.14 (m, 1H, $CH(CH_3)_2$), 4.04 (s, 2H, $ArCH_2$), 4.25 (s, 2H, $SCH_2S$), 6.91 (d, 2H, C*H* Ar), 7.06-7.13 (m, 1H, $C_4$-*H* Ar), 7.20-7.28 (m, 4H, $C_{3,5}$-*H* and C*H* Ar rings), 11.83 (br s, 1H, CON*H*). MS (ESI), m/z: 469 $[M + H]^+$.

*6-(2,6-Dichlorobenzyl)-5-isopropyl-2-({[(4-methoxyphenyl)thio]methyl}thio)pyrimidin-4(3H)-one* ***(23o, MC2972).*** Yield, 28%, mp 168-169°C. $^1$H-NMR ($CDCl_3$) δ 1.34 (t, 6H, $CH(CH_3)_2$), 3.09-3.18 (m, 1H, $CH(CH_3)_2$), 3.73 (s, 3H), 3.99 (s, 2H, $ArCH_2$), 4.25 (s, 2H, $SCH_2S$), 6.76 (d, 2H, C*H* Ar), 7.07-7.12 (m, 1H, $C_4$-*H* Ar), 7.20-7.29 (m, 4H, $C_{3,5}$-*H* and C*H* Ar rings), 12.40 (br s, 1H, CON*H*). MS (ESI), m/z: 481 $[M + H]^+$.

*6-(3,5-Dimethylbenzyl)-5-methyl-2-{[(methylthio)methyl]thio}pyrimidin-4(3H)-one* ***(24a, MC2941).*** Yield, 26%, mp 154-155°C. $^1$H-NMR (DMSO-$d_6$) δ: 1.95 (s, 3H, $SCH_3$), 2.10 (s, 3H, $C_5$-$CH_3$), 2.23 (s, 6H, $C_{3,5}$-$CH_3$), 3.77 (s, 2H, Ar-$CH_2$), 4.36 (s, 2H, $SCH_2S$), 6.83 (s, 1H, $C_4$-*H* Ar), 6.89 (s, 2H, $C_{2,6}$-*H* Ar), 12.63 (br s, 1H, CON*H*). MS (ESI), m/z: 321 $[M + H]^+$.

*6-(3,5-Dimethylbenzyl)-5-methyl-2-{[2-(methylthio)ethyl]thio}pyrimidin-4(3H)-one* ***(24b, MC2942).*** Yield, 74%, mp 152-153°C. $^1$H-NMR ($CDCl_3$) δ 2.03 (s, 6H, $C_{3,5}$-$CH_3$), 2.21 (s, 6H, $CH_3$ and $SCH_3$), 2.67 (m, 2H, $SCH_2CH_2SCH_3$), 3.20-3.30 (m, 2H, $SCH_2CH_2SCH_3$) 3.75 (s, 2H, $CH_2$Ar), 6.72-6.84 (m, 2H, $C_{2,6}$-*H* Ar), 7.18 (m, 1H, $C_4$-*H* Ar), 12.53 (br s, 1H, CON*H*). MS (ESI), m/z: 335 $[M + H]^+$.

*6-(3,5-Dimethylbenzyl)-5-methyl-2-{[(phenylthio)methyl]thio}pyrimidin-4(3H)-one* ***(24c, MC2943).*** Yield, 58%, mp 165-167°C. $^1$H-NMR ($CDCl_3$) δ 1.94-2.04 (m, 3H, $CH_3$), 2.21 (s, 6H, $C_{3,5}$-$CH_3$), 3.76 (m, 2H, $SCH_2SCH_3$), 4.52-4.59 (m, 2H, $CH_2$Ar), 6.75-6.82 (m, 3H, $C_{2,6}$-*H* and C*H* Ar rings), 7.11-7.21 (m, 3H, $C_4$-*H* and C*H* Ar rings), 7.26-7.34 (m, 2H, C*H* Ar), 12.43 (br s, 1H, CON*H*). MS (ESI), m/z: 383 $[M + H]^+$.

*6-(3,5-Dimethylbenzyl)-2-({[(4-fluorophenyl)thio]methyl}thio)-5-methylpyrimidin-4(3H)-one* ***(24d, MC2944).*** Yield, 47%, mp 168-169 °C. $^1$H-NMR ($CDCl_3$) δ 2.02 (s, 3H, $CH_3$), 2.21 (s, 6H, $C_{3,5}$-$CH_3$), 3.76 (s, 2H, $SCH_2SCH_3$), 4.49 (s, 2H, $CH_2$Ar), 6.77-6.94 (m, 5H, $C_{2,4,6}$-*H* and CH Ar rings), 7.34 (m, 2H, C*H* Ar), 12.58 (br s, 1H, CON*H*). MS (ESI), m/z: 401 $[M + H]^+$.

*6-(3,5-Dimethylbenzyl)-2-({[(4-methoxyphenyl)thio]methyl}thio)-5-methylpyrimidin-4(3H)-one* ***(24e, MC2945).*** Yield, 53%, mp 175-176°C. $^1$H-NMR ($CDCl_3$) δ 2.01 (s, 3H, $CH_3$), 2.22 (s, 6H, $C_{3,5}$-$CH_3$), 3.64-3.80 (s, 5H, $SCH_2SCH_3$ and $OCH_3$), 4.45 (s, 2H, $CH_2$Ar), 6.70-6.87 (m, 5H, $C_{2,4,6}$-*H* and C*H* Ar rings), 7.25-7.39 (m, 2H, C*H* Ar), 12.61 (br s, 1H, CON*H*). MS (ESI), m/z: 413 $[M + H]^+$.

*6-(3,5-Dimethylbenzyl)-5-ethyl-2-{[(methylthio)methyl]thio}pyrimidin-4(3H)-one* ***(24f, MC2949).*** Yield, 59%, mp 175-176°C. $^1$H-NMR (DMSO-$d_6$) δ: 1.01 (t, 3H, $CH_2CH_3$), 2.15 (s, 3H, $SCH_3$), 2.28 (s, 6H, $C_{3,5}$-$CH_3$), 2.48-2.51 (q, 2H, $CH_2CH_3$), 3.83 (s, 2H, Ar$CH_2$), 4.40 (s, 2H, $SCH_2S$), 6.88 (s, 1H, $C_4$-*H* Ar), 6.95 (s, 2H, $C_{2,6}$-*H* Ar) 12.64 (br s, 1H, CON*H*). MS (ESI), m/z: 335 $[M + H]^+$.

*6-(3,5-Dimethylbenzyl)-5-ethyl-2-{[2-(methylthio)ethyl]thio}pyrimidin-4(3H)-one* ***(24g, MC2950).*** Yield, 33%, mp 149-150°C. $^1$H-NMR ($CDCl_3$) δ 1.03 (t, 3H, $CH_2CH_3$), 2.00 (s, 3H, $SCH_3$), 2.21 (s, 6H, $C_{3,5}$-Ar-$CH_3$), 2.50 (q, 2H, $CH_2CH_3$), 2.64 (m, 2H, $SCH_2CH_2SCH_3$), 3.22 (m, 2H, $SCH_2CH_2SCH_3$) 3.75 (s, 2H, $CH_2$Ar), 6.71-6.83 (m, 2H, $C_{2,6}$-*H* Ar), 7.19 (m, 1H, $C_4$-*H* Ar), 12.60 (br s, 1H, CON*H*). MS (ESI), m/z: 349 $[M + H]^+$.

*6-(3,5-Dimethylbenzyl)-5-ethyl-2-{[(phenylthio)methyl]thio}pyrimidin-4(3H)-one* ***(24h, MC2951).*** Yield, 56%, mp 150-152°C. $^1$H-NMR ($CDCl_3$) δ 1.02 (m, 3H, $CH_2CH_3$), 2.15-2.30 (s, 6H, $C_{3,5}$-$CH_3$), 2.47-2.54 (m, 2H, $CH_2CH_3$), 3.73-3.80 (s, 2H, $SCH_2SCH_3$), 4.52-4.57 (s, 2H, $CH_2$Ar), 6.77-6.87 (m, 3H, $C_{2,6}$-*H* and C*H* Ar rings), 7.12-7.27 (m, 3H, $C_4$-*H* and CH Ar rings), 7.28-7.37 (m, 2H, C*H* Ar), 12.56 (br s, 1H, CON*H*). MS (ESI), m/z: 397 $[M + H]^+$.

*6-(3,5-Dimethylbenzyl)-5-ethyl-2-({[(4-fluorophenyl)thio]methyl}thio)pyrimidin-4(3H)-one* ***(24i, MC2952).*** Yield, 50%, mp 148-149°C. $^1$H-NMR ($CDCl_3$) δ 0.92-1.09 (m, 3H, $CH_2CH_3$), 2.11-2.22 (s, 6H, $C_{3,5}$-$CH_3$), 2.41-2.57 (m, 2H, $CH_2CH_3$), 3.68-3.84 (s, 2H, $SCH_2SCH_3$), 4.39-4.56 (s, 2H, $CH_2$Ar), 6.76-6.93 (m, 5H, $C_{2,4,6}$-*H* and C*H* Ar rings), 7.25-7.36 (m, 2H, C*H* Ar), 12.46 (br s, 1H, CON*H*). MS (ESI), m/z: 415 $[M + H]^+$.

*6-(3,5-Dimethylbenzyl)-5-ethyl-2-({[(4-methoxyphenyl)thio]methyl}thio)pyrimidin-4(3H)-one* ***(24j, MC2953).*** Yield, 59%, mp 157-159°C. $^1$H-NMR ($CDCl_3$) δ 0.93-1.10 (m, 3H, $CH_2CH_3$), 2.22 (s, 6H, $C_{3,5}$-$CH_3$), 2.46-2.53 (m, 2H, $CH_2CH_3$), 3.66-3.81 (m, 5H, $SCH_2SCH_3$ and $OCH_3$), 4.43 (s, 2H, $CH_2$Ar), 6.70-6.86 (m, 5H, $C_{2,4,6}$-*H* and C*H* Ar rings), 7.31 (d, 2H, C*H* Ar), 12.51 (br s, 1H, CON*H*). MS (ESI), m/z: 427 $[M + H]^+$.

*6-(3,5-Dimethylbenzyl)-5-isopropyl-2-{[(phenylthio)methyl]thio}pyrimidin-4(3H)-one* ***(24m, MC2948).*** Yield, 48%, mp 124-125°C. $^1$H-NMR ($CDCl_3$) δ 1.20-1.23 (d, 6H, $CH(CH_3)_2$), 2.14-2.30 (s, 6H, $C_{3,5}$-$CH_3$), 2.98-3.07 (m, 1H, $CH(CH_3)_2$), 3.74-3.86 (s, 2H, $SCH_2SCH_3$), 4.50-4.59 (s, 2H, $CH_2$Ar), 6.76-6.87 (m, 3H, $C_{2,6}$-*H* and C*H* Ar rings), 7.11-7.26 (m, 3H, $C_4$-*H* and C*H* Ar rings), 7.26-7.35 (m, 2H, C*H* Ar), 12.40 (br s, 1H, CON*H*). MS (ESI), m/z: 411 $[M + H]^+$.

*6-(3,5-Dimethylbenzyl)-2-({[(4-fluorophenyl)thio]methyl}thio)-5-isopropylpyrimidin-4(3H)-one* ***(24n, MX52).*** Yield, 46%, mp 114-115°C. $^{1}$H-NMR ($CCl_4$) δ 1.20 (d, 6H, CH(C*H*$_3$)$_2$), 2.21 (s, 6H, $C_{3,5}$-C*H*$_3$), 2.98 (d, 1H, C*H*(CH$_3$)$_2$), 3.76 (s, 2H, ArC*H*$_2$), 4.43 (s, 2H, SC*H*$_2$S), 6.70 (m, 3H, $C_4$-*H* and C*H* Ar rings), 6.80-6.86 (m, 2H, C*H* Ar), 7.33 (m, 2H, $C_{2,6}$-*H* and C*H* Ar rings), 13.55 (br s, 1H, CON*H*). MS (ESI), m/z: 429 [M + H]$^+$.

*6-(3,5-Dimethylbenzyl)-5-isopropyl-2-({[(4-methoxyphenyl)thio]methyl}thio)pyrimidin-4(3H)-one* ***(24o, MX53).*** Yield, 40%, mp 128-130°C. $^{1}$H-NMR ($CCl_4$) δ 1.20 (d, 6H, CH(C*H*$_3$)$_2$), 2.21 (s, 6H, $C_{3,5}$-C*H*$_3$), 2.91-3.04 (m, 1H, C*H*(CH$_3$)$_2$), 3.66 (s, 3H, OC*H*$_3$), 3.75 (s, 2H, ArC*H*$_2$), 4.37 (s, 2H, SC*H*$_2$S), 6.61-6.72 (m, 5H, $C_{2,4,6}$-*H* and C*H* Ar rings), 7.28 (m, 2H, C*H* Ar), 13.52 (br s, 1H, CON*H*). MS (ESI), m/z: 441 [M + H]$^+$.

**Table S1. Physical and Chemical Data for the Newly Synthesized Final Compounds 21–24**

| Compd | Lab Code | R | $R_1$ | X | mp (°C) | Recryst solvent[a] | % Yield | Formula[b] |
|---|---|---|---|---|---|---|---|---|
| **21b** | MC3133 | Me | 2,6-$F_2$ | $(CH_2)_2SMe$ | 176-178 | A | 74 | $C_{15}H_{16}F_2N_2OS_2$ |
| **21c** | MC3143 | Me | 2,6-$F_2$ | $CH_2SPh$ | 206-208 | B | 89 | $C_{19}H_{16}F_2N_2OS_2$ |
| **21d** | MC3157 | Me | 2,6-$F_2$ | $CH_2S$(4-FPh) | 154-156 | B | 79 | $C_{19}H_{15}F_3N_2OS_2$ |
| **21e** | MC3195 | Me | 2,6-$F_2$ | $CH_2S$(4-MeOPh) | 178-180 | B | 83 | $C_{20}H_{18}F_2N_2O_2S_2$ |
| **21g** | MC3130 | Et | 2,6-$F_2$ | $(CH_2)_2SMe$ | 171-173 | B | 85 | $C_{16}H_{18}F_2N_2OS_2$ |
| **21h** | MC3144 | Et | 2,6-$F_2$ | $CH_2SPh$ | 168-170 | B | 86 | $C_{20}H_{18}F_2N_2OS_2$ |
| **21i** | MC3149 | Et | 2,6-$F_2$ | $CH_2S$(4-FPh) | 137-139 | B | 73 | $C_{20}H_{17}F_3N_2OS_2$ |
| **21j** | MC3194 | Et | 2,6-$F_2$ | $CH_2S$(4-MeOPh) | 168-170 | B | 80 | $C_{21}H_{20}F_2N_2O_2S_2$ |
| **21l** | MC3137 | *i*-Pr | 2,6-$F_2$ | $(CH_2)_2SMe$ | 153-155 | B | 84 | $C_{17}H_{20}F_2N_2OS_2$ |
| **21m** | MC3145 | *i*-Pr | 2,6-$F_2$ | $CH_2SPh$ | 179-181 | B | 80 | $C_{21}H_{20}F_2N_2OS_2$ |
| **21n** | MC3147 | *i*-Pr | 2,6-$F_2$ | $CH_2S$(4-FPh) | 147-149 | B | 72 | $C_{21}H_{19}F_3N_2OS_2$ |
| **21o** | MC3176 | *i*-Pr | 2,6-$F_2$ | $CH_2S$(4-MeOPh) | 121-123 | B | 77 | $C_{22}H_{22}F_2N_2O_2S_2$ |
| **22a** | MC2576 | Me | 2-Cl-6-F | $CH_2SMe$ | 192-193 | C | 52 | $C_{14}H_{14}ClFN_2OS_2$ |
| **22b** | MC2954 | Me | 2-Cl-6-F | $(CH_2)_2SMe$ | 187-188 | G | 99 | $C_{15}H_{16}ClFN_2OS_2$ |
| **22c** | MC2955 | Me | 2-Cl-6-F | $CH_2SPh$ | 181-183 | A | 64 | $C_{19}H_{16}ClFN_2OS_2$ |
| **22d** | MC2956 | Me | 2-Cl-6-F | $CH_2S$(4-FPh) | 169-170 | A | 61 | $C_{19}H_{15}ClF_2N_2OS_2$ |
| **22e** | MC2957 | Me | 2-Cl-6-F | $CH_2S$(4-MeOPh) | 210-212 | A | 61 | $C_{20}H_{18}ClFN_2O_2S_2$ |
| **22f** | MC2585 | Et | 2-Cl-6-F | $CH_2SMe$ | 167-168 | C | 52 | $C_{15}H_{16}ClFN_2OS_2$ |
| **22g** | MC2958 | Et | 2-Cl-6-F | $(CH_2)_2SMe$ | 170-173 | E | 72 | $C_{16}H_{18}ClFN_2OS_2$ |
| **22h** | MC2959 | Et | 2-Cl-6-F | $CH_2SPh$ | 179-181 | B | 61 | $C_{20}H_{18}ClFN_2OS_2$ |
| **22i** | MC2960 | Et | 2-Cl-6-F | $CH_2S$(4-FPh) | 129-130 | E | 28 | $C_{20}H_{17}ClF_2N_2OS_2$ |
| **22j** | MC2961 | Et | 2-Cl-6-F | $CH_2S$(4-MeOPh) | 181-183 | E | 58 | $C_{21}H_{20}ClFN_2O_2S_2$ |
| **22k** | MC2586 | *i*-Pr | 2-Cl-6-F | $CH_2SMe$ | 172-173 | C | 43 | $C_{16}H_{18}ClFN_2OS_2$ |
| **22l** | MC2962 | *i*-Pr | 2-Cl-6-F | $(CH_2)_2SMe$ | 165-166 | E | 76 | $C_{17}H_{20}ClFN_2OS_2$ |
| **22m** | MC2963 | *i*-Pr | 2-Cl-6-F | $CH_2SPh$ | 201-202 | E | 54 | $C_{21}H_{20}ClFN_2OS_2$ |
| **22n** | MC2964 | *i*-Pr | 2-Cl-6-F | $CH_2S$(4-FPh) | 175-177 | E | 53 | $C_{21}H_{19}ClF_2N_2OS_2$ |
| **22o** | MC2965 | *i*-Pr | 2-Cl-6-F | $CH_2S$(4-MeOPh) | 125-127 | E | 54 | $C_{22}H_{22}ClFN_2O_2S_2$ |
| **23a** | MC2579 | Me | 2,6-$Cl_2$ | $CH_2SMe$ | 228-229 | C | 53 | $C_{14}H_{14}Cl_2N_2OS_2$ |
| **23b** | MC2966 | Me | 2,6-$Cl_2$ | $(CH_2)_2SMe$ | 189-190 | G | 97 | $C_{15}H_{16}Cl_2N_2OS_2$ |
| **23c** | MC2967 | Me | 2,6-$Cl_2$ | $CH_2SPh$ | 196-197 | A | 68 | $C_{19}H_{16}Cl_2N_2OS_2$ |
| **23d** | MC2968 | Me | 2,6-$Cl_2$ | $CH_2S$(4-FPh) | 187-188 | A | 70 | $C_{19}H_{15}Cl_2FN_2OS_2$ |
| **23e** | MC2969 | Me | 2,6-$Cl_2$ | $CH_2S$(4-MeOPh) | 221-222 | A | 64 | $C_{20}H_{18}Cl_2N_2O_2S_2$ |
| **23f** | MC2569 | Et | 2,6-$Cl_2$ | $CH_2SMe$ | 203-204 | C | 53 | $C_{15}H_{16}Cl_2N_2OS_2$ |
| **23g** | MX30 | Et | 2,6-$Cl_2$ | $(CH_2)_2SMe$ | 203-205 | B | 35 | $C_{16}H_{18}Cl_2N_2OS_2$ |
| **23h** | MX31 | Et | 2,6-$Cl_2$ | $CH_2SPh$ | 168-169 | B | 30 | $C_{20}H_{18}Cl_2N_2OS_2$ |
| **23i** | MX32 | Et | 2,6-$Cl_2$ | $CH_2S$(4-FPh) | 165-166 | B | 31 | $C_{20}H_{17}Cl_2FN_2OS_2$ |
| **23j** | MX33 | Et | 2,6-$Cl_2$ | $CH_2S$(4-MeOPh) | 168-170 | E | 27 | $C_{21}H_{20}Cl_2N_2O_2S_2$ |
| **23k** | MC2572 | *i*-Pr | 2,6-$Cl_2$ | $CH_2SMe$ | 213-214 | C | 44 | $C_{16}H_{18}Cl_2N_2OS_2$ |
| **23l** | MC2970 | *i*-Pr | 2,6-$Cl_2$ | $(CH_2)_2SMe$ | 194-195 | E | 22 | $C_{17}H_{20}Cl_2N_2OS_2$ |
| **23m** | MX54 | *i*-Pr | 2,6-$Cl_2$ | $CH_2SPh$ | 200-201 | E | 47 | $C_{21}H_{20}Cl_2N_2OS_2$ |
| **23n** | MC2971 | *i*-Pr | 2,6-$Cl_2$ | $CH_2S$(4-FPh) | 198-200 | F | 26 | $C_{21}H_{19}Cl_2FN_2OS_2$ |
| **23o** | MC2972 | *i*-Pr | 2,6-$Cl_2$ | $CH_2S$(4-MeOPh) | 168-169 | E | 28 | $C_{22}H_{22}Cl_2N_2O_2S_2$ |
| **24a** | MC2941 | Me | 3,5- $Me_2$ | $CH_2SMe$ | 154-155 | E | 24 | $C_{16}H_{20}N_2OS_2$ |
| **24b** | MC2942 | Me | 3,5-$Me_2$ | $(CH_2)_2SMe$ | 152-153 | E | 74 | $C_{17}H_{22}N_2OS_2$ |
| **24c** | MC2943 | Me | 3,5-$Me_2$ | $CH_2SPh$ | 165-167 | A | 58 | $C_{21}H_{22}N_2OS_2$ |
| **24d** | MC2944 | Me | 3,5-$Me_2$ | $CH_2S$(4-FPh) | 168-169 | A | 47 | $C_{21}H_{21}FN_2OS_2$ |
| **24e** | MC2945 | Me | 3,5-$Me_2$ | $CH_2S$(4-MeOPh) | 175-176 | A | 53 | $C_{22}H_{24}N_2O_2S_2$ |
| **24f** | MC2949 | Et | 3,5-$Me_2$ | $CH_2SMe$ | 175-176 | E | 59 | $C_{17}H_{22}N_2OS_2$ |
| **24g** | MC2950 | Et | 3,5-$Me_2$ | $(CH_2)_2SMe$ | 149-150 | E | 33 | $C_{18}H_{24}N_2OS_2$ |
| **24h** | MC2951 | Et | 3,5-$Me_2$ | $CH_2SPh$ | 150-152 | E | 56 | $C_{22}H_{24}N_2OS_2$ |
| **24i** | MC2952 | Et | 3,5-$Me_2$ | $CH_2S$(4-FPh) | 148-149 | B | 50 | $C_{22}H_{23}FN_2OS_2$ |
| **24j** | MC2953 | Et | 3,5-$Me_2$ | $CH_2S$(4-MeOPh) | 157-159 | B | 59 | $C_{23}H_{26}N_2O_2S_2$ |
| **24l** | MC2947 | *i*-Pr | 3,5-$Me_2$ | $(CH_2)_2SMe$ | 106-107 | D | 35 | $C_{19}H_{26}N_2OS$ |
| **24m** | MC2948 | *i*-Pr | 3,5-$Me_2$ | $CH_2SPh$ | 124-125 | E | 48 | $C_{23}H_{26}N_2OS_2$ |
| **24n** | MX52 | *i*-Pr | 3,5-$Me_2$ | $CH_2S$(4-FPh) | 114-115 | E | 46 | $C_{23}H_{25}FN_2OS_2$ |
| **24o** | MX53 | *i*-Pr | 3,5-$Me_2$ | $CH_2S$(4-MeOPh) | 128-130 | D | 40 | $C_{24}H_{28}N_2O_2S_2$ |

[a]A: 95% ethanol; B: acetonitrile; C: acetonitrile/THF; D: *n*-hexane; E: cyclohexane; F: *n*-hexane/toluene; G: aqueous ethanol. [b]Analytic results were within ±0.40% of the theoretical values.

**Table S2. Elemental Analyses for the Newly Synthesized Final Compounds 21-24.**

| Compd | Formula | Calculated. % | | | | | | Found. % | | | | | |
|---|---|---|---|---|---|---|---|---|---|---|---|---|---|
| | | C | H | N | S | F | Cl | C | H | N | S | F | Cl |
| **21b** | $C_{15}H_{16}F_2N_2OS_2$ | 52.61 | 4.71 | 8.18 | 18.73 | 11.10 | - | 52.70 | 4.73 | 8.13 | 18.72 | 11.08 | - |
| **21c** | $C_{19}H_{16}F_2N_2OS_2$ | 58.45 | 4.13 | 7.17 | 16.42 | 9.73 | - | 58.58 | 4.15 | 7.13 | 16.41 | 9.72 | - |
| **21d** | $C_{19}H_{15}F_3N_2OS_2$ | 55.87 | 3.70 | 6.86 | 15.70 | 13.95 | - | 56.00 | 3.72 | 6.82 | 15.69 | 13.94 | - |
| **21e** | $C_{20}H_{18}F_2N_2O_2S_2$ | 57.13 | 4.31 | 6.66 | 15.25 | 9.04 | - | 57.25 | 4.33 | 6.62 | 15.24 | 9.02 | - |
| **21g** | $C_{16}H_{18}F_2N_2OS_2$ | 53.91 | 5.09 | 7.86 | 17.99 | 10.66 | - | 54.02 | 5.11 | 7.80 | 17.98 | 10.65 | - |
| **21h** | $C_{20}H_{18}F_2N_2OS_2$ | 59.39 | 4.49 | 6.93 | 15.85 | 9.39 | - | 59.52 | 4.51 | 6.89 | 15.84 | 9.38 | - |
| **21i** | $C_{20}H_{17}F_3N_2OS_2$ | 56.86 | 4.06 | 6.63 | 15.18 | 13.49 | - | 56.96 | 4.08 | 6.57 | 15.16 | 13.47 | - |
| **21j** | $C_{21}H_{20}F_2N_2O_2S_2$ | 58.05 | 4.64 | 6.45 | 14.76 | 8.74 | - | 58.16 | 4.66 | 6.39 | 14.74 | 8.73 | - |
| **21l** | $C_{17}H_{20}F_2N_2OS_2$ | 55.11 | 5.44 | 7.56 | 17.31 | 10.26 | - | 55.2 | 5.46 | 7.52 | 17.3 | 10.24 | - |
| **21m** | $C_{21}H_{20}F_2N_2OS_2$ | 60.27 | 4.82 | 6.69 | 15.32 | 9.08 | - | 60.38 | 4.84 | 6.65 | 15.31 | 9.07 | - |
| **21n** | $C_{21}H_{19}F_3N_2OS_2$ | 57.78 | 4.39 | 6.42 | 14.69 | 13.06 | - | 57.87 | 4.41 | 6.36 | 14.68 | 13.04 | - |
| **21o** | $C_{22}H_{22}F_2N_2O_2S_2$ | 58.91 | 4.94 | 6.25 | 14.30 | 8.47 | - | 58.99 | 4.96 | 6.19 | 14.28 | 8.46 | - |
| **22a** | $C_{14}H_{14}ClFN_2OS_2$ | 48.76 | 4.09 | 8.12 | 18.59 | 5.51 | 10.28 | 48.87 | 4.11 | 8.07 | 18.57 | 5.49 | 10.26 |
| **22b** | $C_{15}H_{16}ClFN_2OS_2$ | 50.20 | 4.49 | 7.81 | 17.87 | 5.29 | 9.88 | 50.33 | 4.51 | 7.76 | 17.86 | 5.28 | 9.87 |
| **22c** | $C_{19}H_{16}ClFN_2OS_2$ | 56.08 | 3.96 | 6.88 | 15.76 | 4.67 | 8.71 | 56.16 | 3.98 | 6.82 | 15.75 | 4.66 | 8.69 |
| **22d** | $C_{19}H_{15}ClF_2N_2OS_2$ | 53.71 | 3.56 | 6.59 | 15.09 | 8.94 | 8.34 | 53.83 | 3.58 | 6.54 | 15.08 | 8.93 | 8.32 |
| **22e** | $C_{20}H_{18}ClFN_2O_2S_2$ | 54.98 | 4.15 | 6.41 | 14.67 | 4.35 | 8.11 | 55.08 | 4.17 | 6.36 | 14.65 | 4.34 | 8.10 |
| **22f** | $C_{15}H_{16}ClFN_2OS_2$ | 50.20 | 4.49 | 7.81 | 17.87 | 5.29 | 9.88 | 50.31 | 4.51 | 7.75 | 17.86 | 5.28 | 9.87 |
| **22g** | $C_{16}H_{18}ClFN_2OS_2$ | 51.54 | 4.87 | 7.51 | 17.19 | 5.09 | 9.51 | 51.62 | 4.89 | 7.46 | 17.17 | 5.08 | 9.49 |
| **22h** | $C_{20}H_{18}ClFN_2OS_2$ | 57.07 | 4.31 | 6.66 | 15.23 | 4.51 | 8.42 | 57.19 | 4.33 | 6.61 | 15.21 | 4.50 | 8.41 |
| **22i** | $C_{20}H_{17}ClF_2N_2OS_2$ | 54.73 | 3.90 | 6.38 | 14.61 | 8.66 | 8.08 | 54.81 | 3.92 | 6.34 | 14.59 | 8.64 | 8.06 |
| **22j** | $C_{21}H_{20}ClFN_2O_2S_2$ | 55.93 | 4.47 | 6.21 | 14.22 | 4.21 | 7.86 | 56.03 | 4.49 | 6.16 | 14.21 | 4.19 | 7.85 |
| **22k** | $C_{16}H_{18}ClFN_2OS_2$ | 51.54 | 4.87 | 7.51 | 17.19 | 5.09 | 9.51 | 51.64 | 4.89 | 7.47 | 17.18 | 5.07 | 9.49 |
| **22l** | $C_{17}H_{20}ClFN_2OS_2$ | 52.77 | 5.21 | 7.24 | 16.57 | 4.91 | 9.16 | 52.87 | 5.23 | 7.20 | 16.56 | 4.89 | 9.15 |
| **22m** | $C_{21}H_{20}ClFN_2OS_2$ | 57.99 | 4.63 | 6.44 | 14.74 | 4.37 | 8.15 | 58.09 | 4.65 | 6.39 | 14.73 | 4.36 | 8.13 |
| **22n** | $C_{21}H_{19}ClF_2N_2OS_2$ | 55.68 | 4.23 | 6.18 | 14.16 | 8.39 | 7.83 | 55.76 | 4.25 | 6.14 | 14.14 | 8.38 | 7.81 |
| **22o** | $C_{22}H_{22}ClFN_2O_2S_2$ | 56.83 | 4.77 | 6.02 | 13.79 | 4.09 | 7.62 | 56.92 | 4.79 | 5.97 | 13.78 | 4.07 | 7.61 |
| **23a** | $C_{14}H_{14}Cl_2N_2OS_2$ | 46.54 | 3.91 | 7.75 | 17.75 | - | 19.62 | 46.65 | 3.93 | 7.69 | 17.73 | - | 19.61 |
| **23b** | $C_{15}H_{16}Cl_2N_2OS_2$ | 48.00 | 4.30 | 7.46 | 17.08 | - | 18.89 | 48.13 | 4.32 | 7.41 | 17.07 | - | 18.88 |
| **23c** | $C_{19}H_{16}Cl_2N_2OS_2$ | 53.90 | 3.81 | 6.62 | 15.15 | - | 16.75 | 54.01 | 3.83 | 6.58 | 15.14 | - | 16.73 |
| **23d** | $C_{19}H_{15}Cl_2FN_2OS_2$ | 51.71 | 3.43 | 6.35 | 14.53 | 4.30 | 16.06 | 51.79 | 3.45 | 6.29 | 14.51 | 4.29 | 16.04 |
| **23e** | $C_{20}H_{18}Cl_2N_2O_2S_2$ | 52.98 | 4.00 | 6.18 | 14.14 | - | 15.64 | 53.09 | 4.02 | 6.12 | 14.12 | - | 15.63 |
| **23f** | $C_{15}H_{16}Cl_2N_2OS_2$ | 48.00 | 4.30 | 7.46 | 17.08 | - | 18.89 | 48.1 | 4.32 | 7.42 | 17.06 | - | 18.87 |
| **23g** | $C_{16}H_{18}Cl_2N_2OS_2$ | 49.36 | 4.66 | 7.20 | 16.47 | - | 18.21 | 49.46 | 4.68 | 7.15 | 16.45 | - | 18.20 |
| **23h** | $C_{20}H_{18}Cl_2N_2OS_2$ | 54.92 | 4.15 | 6.40 | 14.66 | - | 16.21 | 55.01 | 4.17 | 6.35 | 14.65 | - | 16.19 |
| **23i** | $C_{20}H_{17}Cl_2FN_2OS_2$ | 52.75 | 3.76 | 6.15 | 14.08 | 4.17 | 15.57 | 52.84 | 3.78 | 6.09 | 14.07 | 4.15 | 15.55 |
| **23j** | $C_{21}H_{20}Cl_2N_2O_2S_2$ | 53.96 | 4.31 | 5.99 | 13.72 | - | 15.17 | 54.06 | 4.33 | 5.95 | 13.70 | - | 15.16 |
| **23k** | $C_{16}H_{18}Cl_2N_2OS_2$ | 49.36 | 4.66 | 7.20 | 16.47 | - | 18.21 | 49.49 | 4.68 | 7.16 | 16.45 | - | 18.19 |
| **23l** | $C_{17}H_{20}Cl_2N_2OS_2$ | 50.62 | 5.00 | 6.94 | 15.90 | - | 17.58 | 50.73 | 5.02 | 6.89 | 15.89 | - | 17.56 |
| **23m** | $C_{21}H_{20}Cl_2N_2OS_2$ | 55.87 | 4.47 | 6.21 | 14.20 | - | 15.71 | 55.98 | 4.49 | 6.16 | 14.18 | - | 15.69 |
| **23n** | $C_{21}H_{19}Cl_2FN_2OS_2$ | 53.73 | 4.08 | 5.97 | 13.66 | - | 15.10 | 53.81 | 4.10 | 5.91 | 13.64 | - | 15.08 |
| **23o** | $C_{22}H_{22}Cl_2N_2O_2S_2$ | 54.88 | 4.61 | 5.82 | 13.32 | - | 14.73 | 55.00 | 4.63 | 5.76 | 13.31 | - | 14.71 |
| **24a** | $C_{16}H_{20}N_2OS_2$ | 59.97 | 6.29 | 8.74 | 20.01 | - | - | 60.05 | 6.31 | 8.70 | 20.00 | - | - |
| **24b** | $C_{17}H_{22}N_2OS_2$ | 61.04 | 6.63 | 8.37 | 19.17 | - | - | 61.12 | 6.65 | 8.33 | 19.15 | - | - |
| **24c** | $C_{21}H_{22}N_2OS_2$ | 65.94 | 5.80 | 7.32 | 16.76 | - | - | 66.03 | 5.82 | 7.28 | 16.75 | - | - |
| **24d** | $C_{21}H_{21}FN_2OS_2$ | 62.97 | 5.28 | 6.99 | 16.01 | 4.74 | - | 63.07 | 5.30 | 6.94 | 16.00 | 4.73 | - |
| **24e** | $C_{22}H_{24}N_2O_2S_2$ | 64.05 | 5.86 | 6.79 | 15.54 | - | - | 64.13 | 5.88 | 6.75 | 15.53 | - | - |
| **24f** | $C_{17}H_{22}N_2OS_2$ | 61.04 | 6.63 | 8.37 | 19.17 | - | - | 61.13 | 6.65 | 8.32 | 19.15 | - | - |
| **24g** | $C_{18}H_{24}N_2OS_2$ | 62.03 | 6.94 | 8.04 | 18.40 | - | - | 62.12 | 6.96 | 7.99 | 18.38 | - | - |
| **24h** | $C_{22}H_{24}N_2OS_2$ | 66.63 | 6.10 | 7.06 | 16.17 | - | - | 66.76 | 6.12 | 7.00 | 16.15 | - | - |
| **24i** | $C_{22}H_{23}FN_2OS_2$ | 63.74 | 5.59 | 6.76 | 15.47 | 4.58 | - | 63.86 | 5.61 | 6.70 | 15.45 | 4.56 | - |
| **24j** | $C_{23}H_{26}N_2O_2S_2$ | 64.76 | 6.14 | 6.57 | 15.03 | - | - | 64.88 | 6.16 | 6.51 | 15.02 | - | - |
| **24l** | $C_{19}H_{26}N_2OS_2$ | 62.95 | 7.23 | 7.73 | 17.69 | - | - | 63.07 | 7.25 | 7.67 | 17.67 | - | - |

| | | | | | | | | | | | | | |
|---|---|---|---|---|---|---|---|---|---|---|---|---|---|
| **24m** | $C_{23}H_{26}N_2OS_2$ | 67.28 | 6.38 | 6.82 | 15.62 | - | - | 67.41 | 6.40 | 6.77 | 15.61 | - | - |
| **24n** | $C_{23}H_{25}FN_2OS_2$ | 64.46 | 5.88 | 6.54 | 14.96 | 4.43 | - | 64.58 | 5.90 | 6.50 | 14.94 | 4.41 | - |
| **24o** | $C_{24}H_{28}N_2O_2S_2$ | 65.42 | 6.41 | 6.36 | 14.55 | - | - | 65.54 | 6.43 | 6.32 | 14.54 | - | - |
