# Supplementary material for: Systematic Structure–Activity Relationship Investigation on Novel 5‑Alkyl-6-benzyl-2-{[(methylthio/phenylthio)alkyl]thio}-pyrimidin-4(3H)‑ones Endowed with Potent Anti-HIV‑1 Activity
Source: ACS Omega. 2026 Sep 16;11(38):58141–58. doi: 10.1021/acsomega.6c08020 (PMC13629370; doi:10.1021/acsomega.6c08020)
Supplement: Supplementary file 2 [file ao6c08020_si_002.pdf]

Systematic Structure-Activity Relationship Investigation on Novel 5-Alkyl-6-Benzyl-2-{[(Methylthio/Phenylthio)Alkyl]Thio}-Pyrimidin-4(3*H*)-ones Endowed with Potent Anti-HIV-1 Activity

Gebremedhin Solomon Hailu[1,#], Egor Degtyarenko[2,#], Emanuele Fabbrizi[3,#], Anastasia Golovina[4], Andrey Voronkov[2], Andrea Mancini[3], Maxim B. Nawrozkij[2,4,*], Vladimir Chernyshov[5], Laura Bavagnoli[6], Elena Bianchi[6], Roberta Astolfi[3], Rino Ragno[3], José A. Esté[7], Emmanuele Crespan[6], Francesco Fiorentino[8,*], Roman Ivanov[4], Antonello Mai[3,*], Dante Rotili[1,9,*]

SUPPORTING INFORMATION

Content:

Computational investigations on Compounds **21–24**. p. S2

Computational Investigations

1. Computational Medicinal Chemistry Approaches

Ligand-Based (LB) and structure-based (SB) molecular modeling procedures were set up to develop predictive molecular interaction fields (MIF)-based three-dimensional QSARs[1] (MIF 3-D QSAR or CoMFA), and comparative molecular binding energy[2] (COMBINE) models.
The LB and SB procedures were developed through as following.

1.1. Dataset Composition.

1.1.1. LB Dataset

The list of PTM-S-DABO derivatives and potencies against wt HIV1-RT reported in table 3 were directly modeled through the Py-MolEdit module of www.3d-qsar.com web portal.[1, 3] The dataset was splitted into training (TR) and test (TS) and validation (VS) sets with a ratio of 70:20:10, respectively.

1.1.2. SB Dataset

The reported COMBINE procedure[4-7] was replicated using the Py-ComBinE procedure available in the www.3d-qsar.com portal.[3, 8]. The training set was composed of nevirapine and efavirenz modeled complexes into seven different HIV-RT enzymes (WT, L100I, K103N, V106A, V179D, Y181I, Y188L).

1.2. Generation of 3-D conformations for each dataset molecule.

For each dataset's molecule the SMILES[9] structure was obtained through Py-MolEdit section of the 3d-qsar.com website[1]. The dataset's molecules were modeled in a local minima conformation directly from SMILES strings by means of a python script using openbabel.[10-12]

1.3. LB Modeling approaches.

3-D QSAR models were built according to the procedure recently reported by Ragno et al.[8]

1.3.1. 3-D QSAR modeling.

3-D QSAR models were built using the python implemented CoMFA code (Py-CoMFA) procedure, similar as previously reported.[1] The datasets' molecules were investigated for optimal alignment rules through a combination of conformational analyses and automatic molecular alignment methods. The aligned dataset was then subjected to molecular interaction fields (MIFs) calculations and to PLS regression and CV elaborations to obtain r^2, q^2_{cv}, and q^2_{pred} coefficients.

1.3.1.1. Conformational analyses.

Using different programs, a series of conformational analyses were run to inspect the molecule's conformational space.

1.3.1.2. Molecular alignments.

The conformational analyses were merged and used directly for the molecular alignments using different automatic methods, various molecule templates, and different conformations for each template.

1.3.1.3. Calculation of MIFs.

The TRIPOS force field, as re-implemented in the Py-CoMFA, was used to calculate the MIF around the aligned molecules using the same settings as described in the original CoMFA method.[13]

1.3.1.4. Py-CoMFA best models.

Final 3-D QSAR models were selected by means of highest r^2, q^2_{cv}, and q^2_{pred} values and inspected through the average activity contribution (AAC) and activity contribution (AC) plots to delineate a 3-D SAR model. Final predictive ability was then evaluated through the VS.

1.4. SB Modeling approaches.

A structure-based protocol was applied to investigate the likely binding mode of titled derivative into wild type HIV1-RT.

1.4.1. Molecular docking assessment.

A docking assessment procedure was performed using the latest version of the AutoDockVina docking program[4-7, 14, 15] (Version 1.2.7) with the vina scoring function and as previously.

1.4.2. COMBINE modeling.

Through the Py-ComBinE code implemented in the 3d-qsar.com, COMBINE models were built and evaluated through the r^2 and q^2_{cv} coefficient. The model was the applied to the titled derivatives **21-24**.

2. Computational Medicinal Chemistry Results

2.1. LB Dataset Composition.

The list of 60 new synthesized molecules **21-24**, active against WT HIV-1 RT was modeled. The resulting dataset was split into TR, TS and VS with 42 (70%), 12 (20%) and 6 (10%) compounds, respectively. Training set and test set splitting was done keeping the pID_{50} ranges with the same range as the whole dataset (Table S1).

Table S1. List of molecules, SMILES structure, pID_{50}s and set association.

Molecule Name	SMILES	pID_{50}[a]	Set[b]
**21a**	Cc1c(Cc2c(cccc2F)F)nc([nH]c1=O)SCSC	6.98	Training Set
**21b**	Cc1c(Cc2c(cccc2F)F)nc([nH]c1=O)SCCSC	8.10	Training Set
**21c**	Cc1c(Cc2c(cccc2F)F)nc([nH]c1=O)SCSc1ccccc1	7.68	Training Set
**21d**	Cc1c(Cc2c(cccc2F)F)nc([nH]c1=O)SCSc1ccc(cc1)F	8.22	Test Set
**21e**	Cc1c(Cc2c(cccc2F)F)nc([nH]c1=O)SCSc1ccc(cc1)OC	8.70	Test Set
**21f**	CCc1c(Cc2c(cccc2F)F)nc([nH]c1=O)SCSC	7.13	Training Set
**21g**	CCc1c(Cc2c(cccc2F)F)nc([nH]c1=O)SCCSC	7.57	Training Set
**21h**	CCc1c(Cc2c(cccc2F)F)nc([nH]c1=O)SCSc1ccccc1	7.10	Training Set
**21i**	CCc1c(Cc2c(cccc2F)F)nc([nH]c1=O)SCSc1ccc(cc1)F	7.09	Test Set
**21j**	CCc1c(Cc2c(cccc2F)F)nc([nH]c1=O)SCSc1ccc(cc1)OC	7.15	Validation Set
**21k**	CC(C)c1c(Cc2c(cccc2F)F)nc([nH]c1=O)SCSC	7.37	Test Set
**21l**	CC(C)c1c(Cc2c(cccc2F)F)nc([nH]c1=O)SCCSC	7.02	Training Set
**21m**	CC(C)c1c(Cc2c(cccc2F)F)nc([nH]c1=O)SCSc1ccccc1	6.98	Test Set
**21n**	CC(C)c1c(Cc2c(cccc2F)F)nc([nH]c1=O)SCSc1ccc(cc1)F	6.84	Training Set
**21o**	CC(C)c1c(Cc2c(cccc2F)F)nc([nH]c1=O)SCSc1ccc(cc1)OC	6.73	Test Set
**22a**	Cc1c(Cc2c(cccc2Cl)F)nc([nH]c1=O)SCSC	6.57	Training Set
**22b**	Cc1c(Cc2c(cccc2Cl)F)nc([nH]c1=O)SCCSC	7.55	Training Set
**22c**	Cc1c(Cc2c(cccc2Cl)F)nc([nH]c1=O)SCSc1ccccc1	7.51	Validation Set
**22d**	Cc1c(Cc2c(cccc2Cl)F)nc([nH]c1=O)SCSc1ccc(cc1)F	7.96	Training Set
**22e**	Cc1c(Cc2c(cccc2Cl)F)nc([nH]c1=O)SCSc1ccc(cc1)OC	8.22	Training Set
**22f**	CCc1c(Cc2c(cccc2Cl)F)nc([nH]c1=O)SCSC	6.84	Training Set
**22g**	CCc1c(Cc2c(cccc2Cl)F)nc([nH]c1=O)SCCSC	7.10	Training Set
**22h**	CCc1c(Cc2c(cccc2Cl)F)nc([nH]c1=O)SCSc1ccccc1	7.02	Training Set
**22i**	CCc1c(Cc2c(cccc2Cl)F)nc([nH]c1=O)SCSc1ccc(cc1)F	7.09	Test Set
**22j**	CCc1c(Cc2c(cccc2Cl)F)nc([nH]c1=O)SCSc1ccc(cc1)OC	7.15	Training Set
**22k**	CC(C)c1c(Cc2c(cccc2Cl)F)nc([nH]c1=O)SCSC	7.12	Training Set
**22l**	CC(C)c1c(Cc2c(cccc2Cl)F)nc([nH]c1=O)SCCSC	6.90	Training Set
**22m**	CC(C)c1c(Cc2c(cccc2Cl)F)nc([nH]c1=O)SCSc1ccccc1	6.92	Training Set
**22n**	CC(C)c1c(Cc2c(cccc2Cl)F)nc([nH]c1=O)SCSc1ccc(cc1)F	6.77	Training Set
**22o**	CC(C)c1c(Cc2c(cccc2Cl)F)nc([nH]c1=O)SCSc1ccc(cc1)OC	6.40	Validation Set
**23a**	Cc1c(Cc2c(cccc2Cl)Cl)nc([nH]c1=O)SCSC	6.41	Training Set
**23b**	Cc1c(Cc2c(cccc2Cl)Cl)nc([nH]c1=O)SCCSC	7.03	Training Set
**23c**	Cc1c(Cc2c(cccc2Cl)Cl)nc([nH]c1=O)SCSc1ccccc1	7.38	Training Set
**23d**	Cc1c(Cc2c(cccc2Cl)Cl)nc([nH]c1=O)SCSc1ccc(cc1)F	8.00	Training Set
**23e**	Cc1c(Cc2c(cccc2Cl)Cl)nc([nH]c1=O)SCSc1ccc(cc1)OC	8.30	Training Set
**23f**	CCc1c(Cc2c(cccc2Cl)Cl)nc([nH]c1=O)SCSC	6.28	Training Set
**23g**	CCc1c(Cc2c(cccc2Cl)Cl)nc([nH]c1=O)SCCSC	6.19	Training Set
**23h**	CCc1c(Cc2c(cccc2Cl)Cl)nc([nH]c1=O)SCSc1ccccc1	6.44	Test Set
**23i**	CCc1c(Cc2c(cccc2Cl)Cl)nc([nH]c1=O)SCSc1ccc(cc1)F	6.59	Training Set

Molecule Name	SMILES	pID_{50}[a]	Set[b]
**23j**	CCc1c(Cc2c(cccc2Cl)Cl)nc([nH]c1=O)SCSc1ccc(cc1)OC	6.74	Training Set
**23k**	CC(C)c1c(Cc2c(cccc2Cl)Cl)nc([nH]c1=O)SCSC	6.22	Training Set
**23l**	CC(C)c1c(Cc2c(cccc2Cl)Cl)nc([nH]c1=O)SCCSC	6.40	Training Set
**23m**	CC(C)c1c(Cc2c(cccc2Cl)Cl)nc([nH]c1=O)SCSc1ccccc1	6.38	Training Set
**23n**	CC(C)c1c(Cc2c(cccc2Cl)Cl)nc([nH]c1=O)SCSc1ccc(cc1)F	6.03	Training Set
**23o**	CC(C)c1c(Cc2c(cccc2Cl)Cl)nc([nH]c1=O)SCSc1ccc(cc1)OC	6.36	Validation Set
**24a**	Cc1c(Cc2cc(C)cc(C)c2)nc([nH]c1=O)SCSC	6.46	Training Set
**24b**	Cc1c(Cc2cc(C)cc(C)c2)nc([nH]c1=O)SCCSC	7.30	Test Set
**24c**	Cc1c(Cc2cc(C)cc(C)c2)nc([nH]c1=O)SCSc1ccccc1	7.01	Training Set
**24d**	Cc1c(Cc2cc(C)cc(C)c2)nc([nH]c1=O)SCSc1ccc(cc1)F	6.97	Validation Set
**24e**	Cc1c(Cc2cc(C)cc(C)c2)nc([nH]c1=O)SCSc1ccc(cc1)OC	6.89	Training Set
**24f**	CCc1c(Cc2cc(C)cc(C)c2)nc([nH]c1=O)SCSC	7.32	Test Set
**24g**	CCc1c(Cc2cc(C)cc(C)c2)nc([nH]c1=O)SCCSC	7.08	Training Set
**24h**	CCc1c(Cc2cc(C)cc(C)c2)nc([nH]c1=O)SCSc1ccccc1	7.72	Training Set
**24i**	CCc1c(Cc2cc(C)cc(C)c2)nc([nH]c1=O)SCSc1ccc(cc1)F	7.70	Training Set
**24j**	CCc1c(Cc2cc(C)cc(C)c2)nc([nH]c1=O)SCSc1ccc(cc1)OC	7.59	Training Set
**24k**	CC(C)c1c(Cc2cc(C)cc(C)c2)nc([nH]c1=O)SCSC	7.59	Validation Set
**24l**	CC(C)c1c(Cc2cc(C)cc(C)c2)nc([nH]c1=O)SCCSC	7.92	Training Set
**24m**	CC(C)c1c(Cc2cc(C)cc(C)c2)nc([nH]c1=O)SCSc1ccccc1	7.85	Training Set
**24n**	CC(C)c1c(Cc2cc(C)cc(C)c2)nc([nH]c1=O)SCSc1ccc(cc1)F	7.43	Test Set
**24o**	CC(C)c1c(Cc2cc(C)cc(C)c2)nc([nH]c1=O)SCSc1ccc(cc1)OC	7.24	Test Set

a: ID_{50}s from Table 3 were converted into pID_{50} by means of -log10(ID_{50}) with ID_{50} expressed as molar concentration.
b: association of each molecule to training, test and validation sets.

2.1.1. Generation of 3D conformations for each dataset molecule.

Through the Py-MolEdit module, each of the 60 molecule SMILES structures (Supporting Information Table S1) was drawn by hand, converted in 3-D conformations and subjected to a maximum 500 minimization iteration to obtain 60 local minima conformations, which were stored in the www.3d-qsar.com database and used for subsequent elaborations.

2.1.2. LB approaches.

2.1.2.1. <u>3-D QSAR modeling.</u>

3-D QSAR models were built using the Py-CoMFA procedure as previously reported.[1] To this the datasets were subjected for optimal alignment rules definition (conformational analyses and molecular alignments), then molecular interaction fields (MIFs) were calculated and elaborated with PLS and CV to obtain a series of Py-CoMFA models characterized and ranked by q^2_{cv} and q^2_{pred} coefficients.

2.1.2.1.1. <u>Conformational analyses.</u>

On the initial dataset of 60 molecules, 15 different conformational analyses (CAs, Supporting Information Table S2) were run to inspect the molecule's conformational space with different and robust search algorithms and/or force fields.[12] The CAs were performed with three different approaches: the free software Balloon[16] and with a python script through the openbabel and RDKit python libraries. By combining the available force fields and the main features of each method, the CAs were run to collect for each a maximum of 30 conformations for each molecule listed in the dataset.

2.1.2.1.2. <u>Molecular alignments.</u>

Each of the CAs was used in automatic molecular alignments using 6 different methods (Shaep[17], RDKit[18], Align-it[19], Shape-it[20], InterLig[21], and fkcombu[22], Supporting Information Table S3) to generate an aligned dataset to feed the Py-CoMFA module for the MIFs calculations and hence to build the 3-D QSAR models. To this, for each of the 15 CAs, 17 different molecules (Supporting Information Tables S4 and S5) and 3 unique conformations were selected as templates to generate automatically aligned datasets. Considering the different available scoring functions for each alignment method (Supporting Information Tables S3 and S4), thousands of combinations resulted,

and thus too demanding for a systematic approach; therefore, it was decided to randomly select 5 % of them to generate the aligned datasets.[23] A total of 268 automatically aligned datasets were obtained (Supporting Information Table S6).

2.1.2.1.3. Py-CoMFA modeling.

From the above aligned datasets, a total of 268 3-D QSAR models were built (Supporting Information Table S6), and the best were selected by considering the q^2_{cv} and q^2_{TS} values and by visual inspection of molecular alignments. The selected model (Model ID 13 of Supporting Information Table S6) was characterized by a q^2_{cv} of 0.36, a q^2_{pred} of 0.40 and a r^2 of 0.93 at 5 PCs (Supporting Information Tables S7, S8 and Figure S1). The model was obtained with the conformational analysis performed through the RDKit/MMFF94 method and the alignment with ShapeIt/Tanimoto combination software and using as template the conformation with coordinates embracing the highest volume of the molecule with the lowest number of hydrogen bonding acceptor points (**23i**). Lack of chance correlation was confirmed with the Y-scrambling procedure highlighting no better model was obtained after 100 iterations.

Table S2. List of conformational analyses performed on the molecules.

Conf Search ID	Method	Force Field	OB Search Method
1	balloon	EEM	NA
2	balloon	MMFF94	NA
3	rdkit	UFF	NA
4	openbabel	GAFF	Weighted
5	openbabel	GAFF	Random
6	rdkit	MMFF94	NA
7	openbabel	UFF	Weighted
8	openbabel	UFF	Random
9	rdkit	MMFF94s	NA
10	openbabel	MMFF94	Weighted
11	openbabel	MMFF94	Random
12	openbabel	MMFF94s	Weighted
13	openbabel	Ghemical	Weighted
14	openbabel	Ghemical	Random
15	balloon	SFKEEM	NA

Table S3. List of alignment methods and available scoring function/settings varies during automatic alignment combination.

Alignment Method	Available Scoring Function/Settings			
AlignIt	Tanimoto	Tversky		
fkcombu	Atom Flex	Atom Rigid	Volume Flex	Volume Rigid
InterLig				
RDKit	Best Score	Lowest RMSD	Protrude Distance	Tanimoto Distance
Shaep	OnlyShape	Similarity		
ShapeIt	Tanimoto	Tversky		

Table S4. Properties of molecules as obtained from Conf Search ID 1

N	Property	GM		Longest		Biggest	
		Mol Name	Conf	Mol Name	Conf	Mol Name	Conf
1	Least Active	**24e**	0	**24e**	16	**24e**	26
2	Most Active	**21f**	25	**23e**	12	**23e**	7
3	Heaviest	**23n**	15	**23n**	7	**23n**	8
4	Longest	**24i**	22	**24e**	16	**24e**	26

5	Most Flexible	**24j**	17	**24j**	24	**24j**	1
6	Most Rigid	**21c**	28	**23d**	28	**22d**	23
7	Least Polar	**24i**	22	**24i**	22	**24i**	26
8	Most Polar	**23j**	19	**24e**	16	**24e**	26
9	Biggest	**24j**	17	**24j**	24	**24j**	1
10	Highest MR	**23j**	19	**23j**	10	**23j**	25
11	Lowest MR	**21a**	6	**21a**	10	**21a**	17
12	Highest HA	**23j**	19	**24e**	16	**24e**	26
13	Lowest HA	**24i**	22	**24i**	22	**24i**	26
14	Highest HD	**24i**	22	**24e**	16	**24e**	26
15	Lowest HD	**24i**	22	**24e**	16	**24e**	26
16	Highest LogP	**23n**	15	**23n**	7	**23n**	8
17	Lowest LogP	**21a**	6	**21a**	10	**21a**	17

Table S5. Molecular parameters for compounds **21-24** from Conf Search ID 1.

Mol Name	pIC_{50}	MW	HBA	HBD	LogP	TPSA	RBs	MR	Length	Volume
**21a**	8.22	328.05	4.00	1.00	3.36	45.75	7.00	83.00	9.72	341.63
**21b**	8.54	342.07	4.00	1.00	3.40	45.75	8.00	87.87	12.51	365.25
**21c**	8.40	390.07	4.00	1.00	4.79	45.75	7.00	101.78	15.80	412.38
**21d**	8.70	408.06	4.00	1.00	4.93	45.75	7.00	101.74	12.02	412.63
**21e**	9.10	420.08	5.00	1.00	4.80	54.98	9.00	108.33	10.00	445.25
**21f**	8.70	342.07	4.00	1.00	3.61	45.75	8.00	87.64	12.00	366.00
**21g**	8.22	356.08	4.00	1.00	3.66	45.75	9.00	92.51	13.34	391.75
**21h**	8.30	404.08	4.00	1.00	5.04	45.75	8.00	106.42	13.33	436.38
**21i**	8.05	422.07	4.00	1.00	5.18	45.75	8.00	106.38	14.01	437.75
**21j**	8.52	434.09	5.00	1.00	5.05	54.98	10.00	112.98	17.44	468.63
**21k**	9.05	356.08	4.00	1.00	4.18	45.75	9.00	92.35	11.98	389.50
**21l**	8.16	370.10	4.00	1.00	4.22	45.75	10.00	97.22	13.33	412.63
**21m**	7.92	418.10	4.00	1.00	5.60	45.75	9.00	111.13	13.58	460.00
**21n**	7.60	436.09	4.00	1.00	5.74	45.75	9.00	111.09	13.19	460.38
**21o**	7.48	448.11	5.00	1.00	5.61	54.98	11.00	117.69	13.31	488.50
**22a**	7.54	344.02	4.00	1.00	3.87	45.75	7.00	88.05	10.58	359.38
**22b**	7.75	358.04	4.00	1.00	3.92	45.75	8.00	92.92	10.60	382.75
**22c**	7.70	406.04	4.00	1.00	5.30	45.75	7.00	106.83	11.89	426.00
**22d**	8.05	424.03	4.00	1.00	5.44	45.75	7.00	106.79	13.36	428.00
**22e**	8.40	436.05	5.00	1.00	5.31	54.98	9.00	113.39	9.93	460.75
**22f**	7.70	358.04	4.00	1.00	4.13	45.75	8.00	92.69	11.99	383.38
**22g**	7.72	372.05	4.00	1.00	4.17	45.75	9.00	97.56	10.66	408.88
**22h**	6.39	420.05	4.00	1.00	5.56	45.75	8.00	111.48	10.43	450.63
**22i**	6.14	438.04	4.00	1.00	5.70	45.75	8.00	111.43	14.31	453.75
**22j**	6.29	450.06	5.00	1.00	5.57	54.98	10.00	118.03	11.40	483.00
**22k**	8.00	372.05	4.00	1.00	4.69	45.75	9.00	97.40	10.98	407.63
**22l**	6.23	386.07	4.00	1.00	4.73	45.75	10.00	102.27	13.48	430.25
**22m**	6.06	434.07	4.00	1.00	6.12	45.75	9.00	116.19	14.45	474.13
**22n**	6.00	452.06	4.00	1.00	6.26	45.75	9.00	116.14	14.14	474.38
**22o**	6.13	464.08	5.00	1.00	6.13	54.98	11.00	122.74	15.76	507.00
**23a**	6.96	359.99	4.00	1.00	4.39	45.75	7.00	93.10	10.67	374.50
**23b**	7.10	374.01	4.00	1.00	4.43	45.75	8.00	97.97	13.25	398.50
**23c**	7.82	422.01	4.00	1.00	5.82	45.75	7.00	111.89	14.26	441.75
**23d**	8.30	440.00	4.00	1.00	5.96	45.75	7.00	111.84	14.45	443.38
**23e**	8.70	452.02	5.00	1.00	5.83	54.98	9.00	118.44	9.67	475.63

Mol Name	pIC_{50}	MW	HBA	HBD	LogP	TPSA	RBs	MR	Length	Volume
**23f**	6.72	374.01	4.00	1.00	4.64	45.75	8.00	97.74	9.62	400.13
**23g**	6.25	388.02	4.00	1.00	4.69	45.75	9.00	102.61	10.45	422.38
**23h**	6.23	436.02	4.00	1.00	6.07	45.75	8.00	116.53	14.86	469.50
**23i**	6.48	454.01	4.00	1.00	6.21	45.75	8.00	116.49	12.82	469.13
**23j**	6.68	466.03	5.00	1.00	6.08	54.98	10.00	123.08	16.68	501.13
**23k**	6.55	388.02	4.00	1.00	5.20	45.75	9.00	102.45	12.15	421.88
**23l**	6.35	402.04	4.00	1.00	5.25	45.75	10.00	107.32	11.79	443.13
**23m**	6.35	450.04	4.00	1.00	6.63	45.75	9.00	121.24	13.39	487.25
**23n**	6.08	468.03	4.00	1.00	6.77	45.75	9.00	121.20	13.36	490.63
**23o**	6.25	480.05	5.00	1.00	6.64	54.98	11.00	127.79	10.57	521.25
**24a**	5.74	320.10	4.00	1.00	3.70	45.75	9.00	92.56	11.45	389.13
**24b**	6.68	334.12	4.00	1.00	3.74	45.75	10.00	97.42	14.23	410.88
**24c**	6.13	382.12	4.00	1.00	5.13	45.75	9.00	111.34	12.79	456.38
**24d**	5.50	400.11	4.00	1.00	5.27	45.75	9.00	111.30	13.20	456.25
**24e**	5.39	412.13	5.00	1.00	5.14	54.98	11.00	117.89	10.96	489.88
**24f**	6.11	334.12	4.00	1.00	3.95	45.75	10.00	97.20	12.01	411.00
**24g**	6.75	348.13	4.00	1.00	4.00	45.75	11.00	102.06	13.97	436.63
**24h**	6.42	396.13	4.00	1.00	5.38	45.75	10.00	115.98	16.47	478.63
**24i**	6.15	414.12	4.00	1.00	5.52	45.75	10.00	115.94	17.29	481.25
**24j**	6.14	426.14	5.00	1.00	5.39	54.98	12.00	122.53	16.56	513.75
**24k**	6.94	348.13	4.00	1.00	4.51	45.75	11.00	101.91	11.80	436.38
**24l**	7.57	362.15	4.00	1.00	4.56	45.75	12.00	106.78	11.73	456.88
**24m**	7.31	410.15	4.00	1.00	5.94	45.75	11.00	120.69	15.28	503.00
**24n**	7.25	428.14	4.00	1.00	6.08	45.75	11.00	120.65	14.83	505.38
**24o**	7.09	440.16	5.00	1.00	5.95	54.98	13.00	127.24	11.08	534.63

Table S6. list of Py-CoMFA models r^2, q^2_{cv} and q^2_{TS} coefficients and associated alignment rules.

Model ID	$r^{2,a}$	q^2_{cv} [b]	q^2_{TS} [c]	Alignment[d]		Conf Search ID[e]	Reference Mol[f]		Conformation[g]		
				Method	Scoring Function		Parameter	Molecule	Reference	Longest	Biggest
1	0.49	0.18	-0.09	AlignIt	Tanimoto	6	Highest MR	23j	28	false	true
2	0.48	0.09	-0.39	AlignIt	Tanimoto	9	Lowest HD	24j	26	false	true
3	0.90	0.37	0.34	RDKit	Protrude Distance	1	Most Active	23e	12	true	false
4	0.73	-0.01	-0.57	FKCOMBU	Volume Rigid	8	Least Active	24e	29	false	true
5	0.71	-0.04	0.36	RDKit	Tanimoto Distance	5	Highest MR	23j	21	true	false
6	0.54	0.05	-0.81	ShapeIt	Tversky	5	Most Rigid	22d	0	false	false
7	0.68	0.19	0.06	RDKit	Best Score	1	Lowest HA	24i	26	false	true
8	0.86	0.35	-0.01	RDKit	Lowest RMSD	1	Biggest	24j	24	true	false
9	0.41	0.12	-0.90	RDKit	Lowest RMSD	4	Least Active	24e	15	true	false
10	0.59	0.02	-0.50	ShapeIt	Tanimoto	4	Lowest HD	24j	9	false	true
11	0.64	-0.42	-0.29	InterLig		4	Most Active	23e	17	false	true
12	0.91	0.19	0.15	RDKit	Best Score	3	Lowest LogP	21a	12	false	true
13	0.93	0.36	0.41	ShapeIt	Tanimoto	9	Lowest HA	23i	13	false	true
14	0.46	-0.21	-0.07	FKCOMBU	Volume Flex	6	Least Active	24e	15	false	false
15	0.56	-0.13	-0.52	FKCOMBU	Atom Flex	2	Most Active	23e	27	false	true
16	0.78	-0.04	-0.48	Shaep	OnlyShape	7	Most Active	23e	10	true	false
17	0.25	-0.05	-0.65	AlignIt	Tanimoto	3	Lowest LogP	21a	0	true	false
18	0.46	0.02	0.09	FKCOMBU	Volume Rigid	3	Lowest HD	24j	11	false	false
19	0.27	-0.02	-0.30	RDKit	Protrude Distance	3	Highest MR	23j	23	true	false
20	0.38	0.13	-0.16	RDKit	Protrude Distance	9	Highest MR	23j	15	false	false
21	0.41	0.14	0.17	RDKit	Protrude Distance	9	Lowest HD	24j	7	true	false
22	0.32	0.08	0.12	RDKit	Lowest RMSD	9	Most Rigid	23d	26	false	false
23	0.51	-0.06	0.13	AlignIt	Tanimoto	7	Least Active	24e	28	true	false
24	0.46	-0.04	-0.21	FKCOMBU	Atom Rigid	8	Highest LogP	23n	27	false	true
25	0.43	0.12	0.12	RDKit	Tanimoto Distance	6	Lowest HD	24e	26	false	true
26	0.90	0.37	0.34	RDKit	Lowest RMSD	1	Most Active	23e	12	true	false

Model ID	$r^{2,a}$	q^2_{cv}[b]	q^2_{TS}[c]	Alignment[d]		Conf Search ID[e]	Reference Mol[f]		Conformation[g]		
				Method	Scoring Function		Parameter	Molecule	Reference	Longest	Biggest
27	0.88	0.42	0.31	RDKit	Tanimoto Distance	1	Most Rigid	22d	23	false	true
28	0.56	-0.91	-0.74	FKCOMBU	Volume Rigid	5	Lowest HA	24h	9	false	true
29	0.88	-0.09	0.01	RDKit	Protrude Distance	5	Highest LogP	23n	7	true	false
30	0.30	0.04	-0.01	RDKit	Best Score	7	Highest MR	23j	1	false	false
31	0.53	-0.12	-0.27	FKCOMBU	Atom Flex	8	Most Active	23e	22	false	true
32	0.46	-0.04	-0.21	FKCOMBU	Volume Rigid	8	Highest LogP	23n	27	false	true
33	0.79	0.12	0.30	RDKit	Tanimoto Distance	8	Least Active	24e	4	false	false
34	0.50	0.06	0.20	RDKit	Tanimoto Distance	6	Highest MR	23j	28	false	true
35	0.72	-0.06	0.09	RDKit	Lowest RMSD	3	Least Active	24e	16	false	false
36	0.52	0.07	0.01	Shaep	Similarity	6	Lowest HA	24i	9	true	false
37	0.43	-0.06	0.05	Shaep	Similarity	1	Biggest	24j	17	false	false
38	0.86	-0.23	-0.31	FKCOMBU	Atom Flex	4	Least Active	24e	18	false	true
39	0.41	0.12	-0.90	RDKit	Best Score	4	Least Active	24e	25	false	false
40	0.98	0.27	0.11	InterLig		6	Highest LogP	23n	2	true	false
41	0.29	-0.04	-0.04	InterLig		5	Most Rigid	22d	29	true	false
42	0.91	0.19	0.15	RDKit	Tanimoto Distance	3	Lowest LogP	21a	12	false	true
43	0.42	0.09	0.08	RDKit	Best Score	6	Lowest HD	24j	9	true	false
44	0.58	0.00	-0.04	AlignIt	Tanimoto	9	Highest MR	23j	27	false	true
45	0.60	-0.36	0.12	AlignIt	Tversky	5	Lowest HA	24c	20	true	false
46	0.74	-0.41	-0.29	FKCOMBU	Atom Flex	5	Lowest LogP	21a	3	false	false
47	0.46	0.02	0.09	FKCOMBU	Atom Flex	3	Lowest HD	24j	23	true	false
48	0.96	0.30	-0.41	AlignIt	Tanimoto	4	Highest LogP	23n	15	false	false
49	0.32	-0.16	-0.10	FKCOMBU	Volume Flex	4	Highest LogP	23n	15	false	false
50	0.77	0.02	-0.77	FKCOMBU	Atom Flex	4	Highest MR	23j	9	true	false
51	0.53	-0.12	-0.88	FKCOMBU	Volume Rigid	4	Most Rigid	22d	8	false	true
52	0.31	-0.10	0.06	Shaep	OnlyShape	9	Lowest HA	24h	17	true	false
53	0.31	-0.15	0.21	FKCOMBU	Volume Flex	6	Lowest HD	24j	9	true	false
54	0.54	-0.09	-0.26	AlignIt	Tversky	7	Lowest HD	24j	14	false	true
55	0.68	-0.17	-0.43	AlignIt	Tanimoto	9	Lowest LogP	21a	19	false	true
56	0.68	-0.17	-0.43	AlignIt	Tanimoto	9	Lowest LogP	21a	25	true	false
57	0.84	0.10	0.50	RDKit	Best Score	8	Lowest HD	24i	7	false	false
58	0.49	-0.58	-0.40	FKCOMBU	Atom Flex	8	Most Rigid	22d	29	true	false
59	0.76	0.10	0.36	RDKit	Protrude Distance	8	Highest MR	23j	27	false	true
60	0.48	-0.21	-0.39	ShapeIt	Tversky	5	Lowest HA	24c	20	true	false
61	0.73	-0.05	0.08	Shaep	Similarity	3	Highest MR	23j	9	false	false
62	0.31	-0.15	0.21	FKCOMBU	Atom Flex	6	Lowest HD	24j	9	true	false
63	0.37	-0.19	0.22	AlignIt	Tanimoto	6	Biggest	24j	23	false	false
64	0.53	-0.13	0.16	ShapeIt	Tanimoto	6	Most Active	23e	24	true	false
65	0.68	0.19	0.06	RDKit	Lowest RMSD	1	Lowest HA	24i	22	true	false
66	0.47	0.18	-0.36	ShapeIt	Tanimoto	1	Lowest HA	24i	22	true	false
67	0.90	0.37	0.34	RDKit	Lowest RMSD	1	Most Active	23e	7	false	true
68	0.46	0.02	0.09	FKCOMBU	Volume Rigid	3	Lowest HD	24j	23	true	false
69	0.46	-0.19	-0.15	ShapeIt	Tversky	5	Lowest HA	24h	9	false	true
70	0.60	-0.12	-0.39	InterLig		5	Highest MR	23j	21	true	false
71	0.53	-0.05	-1.14	FKCOMBU	Atom Flex	3	Most Rigid	22d	2	false	false
72	0.27	-0.02	-0.30	RDKit	Best Score	3	Highest MR	23j	23	true	false
73	0.41	0.04	-0.30	AlignIt	Tversky	3	Most Active	23e	12	false	false
74	0.77	-0.61	-0.21	FKCOMBU	Atom Rigid	4	Lowest HA	24i	21	true	false
75	0.61	-0.36	0.02	AlignIt	Tanimoto	5	Lowest HD	24c	28	false	false
76	0.40	-0.27	-0.27	RDKit	Protrude Distance	4	Highest LogP	23n	10	true	false
77	0.86	-0.23	-0.31	FKCOMBU	Atom Rigid	4	Least Active	24e	25	false	false
78	0.46	-0.37	0.07	Shaep	OnlyShape	5	Highest HA	24e	11	false	false
79	0.65	-0.18	0.03	FKCOMBU	Volume Flex	5	Lowest HD	24j	6	false	true
80	0.46	-0.06	0.16	FKCOMBU	Volume Flex	1	Lowest HA	24i	26	false	true
81	0.39	-0.13	-0.06	FKCOMBU	Atom Flex	1	Lowest HD	24e	16	true	false
82	0.31	0.06	0.01	RDKit	Tanimoto Distance	9	Most Active	23e	19	false	false
83	0.46	0.13	-0.36	RDKit	Best Score	7	Lowest HA	24g	2	false	false
84	0.49	-0.17	-0.47	RDKit	Best Score	12	Highest LogP	23n	16	true	false
85	0.49	-0.06	-0.56	Shaep	OnlyShape	15	Lowest HA	24m	27	true	false
86	0.43	-0.18	0.21	ShapeIt	Tanimoto	12	Lowest HD	24e	7	true	false

Model ID	$r^{2,a}$	q^2_{cv}[b]	q^2_{TS}[c]	Alignment[d]		Conf Search ID[e]	Reference Mol[f]		Conformation[g]		
				Method	Scoring Function		Parameter	Molecule	Reference	Longest	Biggest
87	0.37	-0.08	-0.54	FKCOMBU	Atom Rigid	15	Least Active	24e	18	true	false
88	0.46	0.14	-0.34	InterLig		11	Highest LogP	23n	21	true	false
89	0.40	0.11	-0.06	FKCOMBU	Volume Rigid	11	Highest LogP	23n	21	false	true
90	0.55	-0.16	-0.31	Shaep	Similarity	7	Lowest HA	22h	8	false	true
91	0.59	-0.21	-0.87	FKCOMBU	Volume Rigid	4	Lowest LogP	21a	19	false	true
92	0.45	-0.31	-0.42	InterLig		15	Lowest LogP	21a	23	false	false
93	0.53	-0.13	0.14	FKCOMBU	Volume Rigid	12	Most Active	23e	23	true	false
94	0.68	-0.05	0.24	RDKit	Lowest RMSD	11	Least Active	24e	25	false	true
95	0.44	-0.08	-0.22	AlignIt	Tversky	13	Lowest HD	24j	8	true	false
96	0.66	-0.17	-0.16	RDKit	Protrude Distance	13	Highest LogP	23n	16	true	false
97	0.49	0.16	0.24	AlignIt	Tanimoto	14	Lowest HA	24i	23	true	false
98	0.71	-0.06	0.44	RDKit	Lowest RMSD	14	Most Rigid	22d	28	false	true
99	0.68	-0.05	0.24	RDKit	Lowest RMSD	11	Lowest HD	24e	24	true	false
100	0.97	-0.37	0.50	InterLig		11	Lowest LogP	21a	25	false	true
101	0.57	0.08	-0.23	FKCOMBU	Atom Flex	11	Lowest HD	24j	1	false	false
102	0.51	0.08	-0.46	AlignIt	Tanimoto	10	Lowest LogP	21a	18	false	false
103	0.65	-0.02	0.06	Shaep	OnlyShape	10	Biggest	24j	22	false	true
104	0.52	-0.26	-0.32	Shaep	Similarity	15	Most Active	21f	8	false	false
105	0.30	0.03	-0.03	Shaep	OnlyShape	15	Highest MR	23j	10	true	false
106	0.61	-0.13	0.00	ShapeIt	Tanimoto	15	Lowest LogP	21a	1	false	true
107	0.78	-0.30	-0.66	FKCOMBU	Atom Flex	13	Most Active	23e	25	true	false
108	0.46	0.01	-0.21	ShapeIt	Tversky	13	Highest MR	23j	12	false	true
109	0.95	0.05	0.36	InterLig		11	Most Active	23e	28	true	false
110	0.40	0.11	-0.06	FKCOMBU	Atom Flex	11	Highest LogP	23n	2	false	false
111	0.48	-0.38	0.10	FKCOMBU	Atom Rigid	10	Lowest HD	24e	4	false	true
112	0.53	-0.12	-0.26	Shaep	OnlyShape	10	Highest LogP	23n	29	false	false
113	0.32	-0.17	-0.50	RDKit	Tanimoto Distance	12	Lowest HD	23e	5	false	false
114	0.41	-0.07	0.42	RDKit	Protrude Distance	12	Least Active	24e	20	false	true
115	0.83	-0.16	-0.67	FKCOMBU	Atom Flex	12	Biggest	24j	2	false	true
116	0.46	-0.74	-0.34	FKCOMBU	Atom Rigid	15	Lowest HD	23e	15	false	true
117	0.97	0.30	0.67	RDKit	Lowest RMSD	14	Highest LogP	23n	7	false	false
118	0.43	-0.98	-0.05	FKCOMBU	Atom Rigid	14	Lowest HA	24i	23	true	false
119	0.71	-0.06	0.44	RDKit	Best Score	14	Most Rigid	22d	28	true	false
120	0.89	0.20	-0.52	Shaep	Similarity	10	Most Active	23e	23	true	false
121	0.43	0.00	-0.25	Shaep	Similarity	2	Lowest HA	24c	20	false	true
122	0.89	0.07	-0.02	AlignIt	Tanimoto	3	Lowest HA	24i	27	false	false
123	0.45	-0.16	0.08	ShapeIt	Tversky	5	Most Active	23e	19	false	true
124	0.48	0.18	-0.02	InterLig		1	Highest LogP	23n	8	false	true
125	0.37	-0.27	-0.49	Shaep	OnlyShape	11	Most Rigid	23d	0	false	false
126	0.55	-0.32	-0.85	Shaep	OnlyShape	14	Lowest HA	24i	11	false	false
127	0.40	-0.16	-0.02	AlignIt	Tversky	14	Highest MR	23j	0	false	false
128	0.50	-0.16	-0.08	Shaep	Similarity	12	Least Active	24e	0	false	false
129	0.54	-0.05	-0.86	Shaep	OnlyShape	4	Lowest HA	24c	12	false	true
130	0.33	-0.15	-0.11	RDKit	Best Score	2	Lowest HA	24i	13	true	false
131	0.39	-0.07	-0.16	ShapeIt	Tversky	2	Lowest HA	24c	20	false	true
132	0.88	-0.09	0.01	RDKit	Lowest RMSD	5	Highest LogP	23n	7	true	false
133	0.71	-0.04	0.36	RDKit	Lowest RMSD	5	Highest MR	23j	0	false	true
134	0.67	0.11	0.23	AlignIt	Tversky	8	Lowest HD	24e	22	true	false
135	0.45	-0.04	-0.40	ShapeIt	Tanimoto	3	Least Active	24e	16	false	false
136	0.46	0.17	-0.11	ShapeIt	Tversky	1	Lowest HA	24i	22	true	false
137	0.45	0.13	-0.25	ShapeIt	Tversky	6	Lowest HD	24e	26	false	true
138	0.79	0.12	0.30	RDKit	Lowest RMSD	8	Least Active	24e	4	false	false
139	0.87	-0.60	-0.29	RDKit	Tanimoto Distance	4	Highest MR	23j	24	false	true
140	0.52	-0.52	-0.03	ShapeIt	Tanimoto	4	Lowest HA	24i	21	true	false
141	0.25	-0.06	0.17	RDKit	Lowest RMSD	8	Lowest LogP	21a	3	false	false
142	0.61	0.10	-0.35	FKCOMBU	Atom Flex	9	Highest MR	23j	15	false	false
143	0.44	-0.27	-0.28	FKCOMBU	Atom Rigid	2	Least Active	24e	5	false	false
144	0.40	-0.27	-0.27	RDKit	Protrude Distance	4	Highest LogP	23n	15	false	false
145	0.42	-0.02	0.32	RDKit	Lowest RMSD	9	Lowest HA	24h	24	false	false
146	0.50	-0.14	0.19	AlignIt	Tversky	2	Most Active	23e	27	false	true

Model ID	$r^{2,a}$	q^2_{cv}[b]	q^2_{TS}[c]	Alignment[d] Method	Alignment[d] Scoring Function	Conf Search ID[e]	Reference Mol[f] Parameter	Reference Mol[f] Molecule	Conformation[g] Reference	Conformation[g] Longest	Conformation[g] Biggest
147	0.40	0.01	-0.04	RDKit	Best Score	2	Highest MR	23j	6	true	false
148	0.42	-0.26	0.14	RDKit	Lowest RMSD	2	Lowest LogP	21a	13	false	true
149	0.35	0.17	-0.09	ShapeIt	Tversky	3	Highest MR	23j	23	true	false
150	0.46	-0.20	0.03	AlignIt	Tversky	6	Least Active	24e	15	false	false
151	0.46	-0.06	0.16	FKCOMBU	Atom Rigid	1	Lowest HA	24i	22	true	false
152	0.41	-0.11	0.08	AlignIt	Tanimoto	1	Biggest	24j	1	false	true
153	0.31	-0.03	0.02	Shaep	OnlyShape	9	Most Active	23e	18	false	true
154	0.74	-0.13	-0.08	Shaep	OnlyShape	8	Lowest HA	24h	23	true	false
155	0.46	0.02	0.09	FKCOMBU	Volume Rigid	3	Biggest	24j	24	false	true
156	0.45	-0.39	-0.28	FKCOMBU	Atom Flex	1	Highest LogP	23n	8	false	true
157	0.65	-0.10	-0.18	AlignIt	Tversky	7	Lowest LogP	21a	1	false	false
158	0.92	-0.08	-0.25	InterLig		2	Most Flexible	24l	17	false	true
159	0.84	0.10	0.50	RDKit	Tanimoto Distance	8	Lowest HD	24i	7	false	false
160	0.75	-0.18	-0.26	RDKit	Protrude Distance	2	Most Flexible	24l	17	false	true
161	0.66	-0.09	0.12	AlignIt	Tanimoto	7	Highest MR	23j	15	false	true
162	0.92	-0.23	-0.41	FKCOMBU	Volume Rigid	9	Lowest HA	24h	17	true	false
163	0.59	-0.09	-0.46	FKCOMBU	Atom Rigid	7	Most Active	23e	10	true	false
164	0.59	-0.15	0.03	InterLig		2	Lowest HA	24c	20	false	true
165	0.49	-0.19	-0.52	AlignIt	Tversky	1	Highest LogP	23n	8	false	true
166	0.44	-0.59	-0.49	FKCOMBU	Atom Rigid	7	Highest LogP	23n	17	true	false
167	0.61	0.10	-0.35	FKCOMBU	Atom Rigid	9	Highest MR	23j	0	true	false
168	0.56	-0.18	-0.79	FKCOMBU	Volume Flex	5	Most Active	23e	19	false	true
169	0.49	-0.30	-0.34	FKCOMBU	Atom Flex	6	Highest MR	23j	25	false	false
170	0.58	-0.44	-0.26	Shaep	Similarity	2	Highest LogP	23n	24	true	false
171	0.34	-0.08	0.40	AlignIt	Tversky	3	Lowest HA	24h	22	false	true
172	0.58	-0.44	-0.26	Shaep	OnlyShape	2	Highest LogP	23n	24	true	false
173	0.56	-0.13	0.15	FKCOMBU	Atom Flex	10	Most Rigid	23c	3	false	false
174	0.97	-0.30	-0.18	FKCOMBU	Volume Rigid	10	Biggest	24j	9	true	false
175	0.55	0.00	0.26	FKCOMBU	Volume Flex	13	Lowest LogP	21a	23	true	false
176	0.50	-0.08	-0.60	FKCOMBU	Atom Rigid	13	Lowest HD	24j	17	false	true
177	0.46	-0.15	0.22	ShapeIt	Tanimoto	14	Highest MR	23j	27	false	true
178	0.38	0.13	-0.16	RDKit	Protrude Distance	9	Highest MR	23j	0	true	false
179	0.56	-0.25	-0.64	FKCOMBU	Volume Rigid	12	Least Active	24e	20	false	true
180	0.46	-0.14	0.19	ShapeIt	Tversky	8	Lowest HD	24i	22	false	true
181	0.48	0.06	-0.10	RDKit	Best Score	15	Highest MR	23j	18	false	false
182	0.56	-0.13	0.01	ShapeIt	Tversky	10	Lowest HD	24e	5	true	false
183	0.45	-0.19	-0.39	ShapeIt	Tversky	12	Highest LogP	23n	6	false	false
184	0.76	-0.25	-0.08	InterLig		15	Lowest HA	24m	20	false	true
185	0.59	0.19	-0.23	FKCOMBU	Volume Rigid	12	Lowest LogP	21a	26	false	true
186	0.48	-0.02	-0.12	RDKit	Lowest RMSD	13	Least Active	24e	6	true	false
187	0.50	-0.05	-0.22	Shaep	OnlyShape	10	Lowest HA	22m	1	false	false
188	0.63	0.07	0.32	ShapeIt	Tversky	13	Highest LogP	23n	3	false	true
189	0.43	-0.98	-0.05	FKCOMBU	Volume Flex	14	Lowest HA	24i	23	true	false
190	0.42	0.03	0.09	ShapeIt	Tanimoto	2	Highest MR	23j	2	false	true
191	0.49	0.17	-1.11	FKCOMBU	Volume Rigid	14	Lowest HA	24m	24	false	true
192	0.42	-0.02	-0.02	ShapeIt	Tanimoto	15	Highest MR	23j	10	true	false
193	0.58	-0.01	-0.07	ShapeIt	Tversky	12	Highest LogP	23n	16	true	false
194	0.43	-0.44	0.31	AlignIt	Tanimoto	7	Most Rigid	22d	22	true	false
195	0.96	0.20	0.20	RDKit	Tanimoto Distance	14	Lowest HA	24i	23	true	false
196	0.40	-0.43	-0.31	Shaep	OnlyShape	4	Most Active	23e	9	false	false
197	0.86	0.18	0.33	RDKit	Tanimoto Distance	15	Lowest LogP	21a	9	true	false
198	0.62	0.27	-0.17	RDKit	Best Score	12	Lowest HD	23e	5	false	false
199	0.55	0.02	-0.48	ShapeIt	Tanimoto	11	Highest LogP	23n	2	false	false
200	0.34	-0.18	-0.02	FKCOMBU	Volume Rigid	15	Most Active	21f	8	false	false
201	0.47	0.09	-0.05	RDKit	Protrude Distance	10	Most Active	23e	2	false	false
202	0.56	-0.25	-0.64	FKCOMBU	Atom Rigid	12	Lowest HD	24e	7	true	false
203	0.54	0.11	-0.32	FKCOMBU	Volume Rigid	14	Highest MR	23j	27	false	true
204	0.68	-0.17	-0.43	AlignIt	Tanimoto	9	Lowest LogP	21a	0	false	false
205	0.73	-0.01	-0.57	FKCOMBU	Atom Rigid	8	Least Active	24e	29	false	true
206	0.66	-0.17	-0.16	RDKit	Best Score	13	Highest LogP	23n	3	false	true

Model ID	r^{2}[a]	q^{2}_{cv}[b]	q^{2}_{TS}[c]	Alignment[d]		Conf Search ID[e]	Reference Mol[f]		Conformation[g]		
				Method	Scoring Function		Parameter	Molecule	Reference	Longest	Biggest
207	0.48	0.06	-0.10	RDKit	Lowest RMSD	15	Highest MR	23j	10	true	false
208	0.53	-0.09	0.29	RDKit	Best Score	12	Lowest LogP	21a	25	false	false
209	0.54	-0.33	-0.58	FKCOMBU	Volume Flex	11	Lowest HD	24e	24	true	false
210	0.38	-0.27	-0.25	Shaep	Similarity	13	Most Rigid	23c	12	true	false
211	0.49	-0.04	0.29	Shaep	Similarity	14	Lowest LogP	21a	29	false	true
212	0.77	-0.31	-0.55	FKCOMBU	Volume Flex	11	Lowest HA	24i	22	true	false
213	0.43	-0.08	0.37	ShapeIt	Tanimoto	14	Lowest HA	24i	23	true	false
214	0.95	-0.10	0.08	RDKit	Best Score	10	Lowest HA	22m	1	false	false
215	0.51	0.22	-0.23	Shaep	Similarity	12	Highest LogP	23n	6	false	false
216	0.65	0.21	0.26	Shaep	Similarity	13	Least Active	24e	6	true	false
217	1.00	0.11	0.39	ShapeIt	Tversky	13	Least Active	24e	25	false	true
218	0.62	0.16	-0.51	ShapeIt	Tanimoto	11	Highest LogP	23n	21	true	false
219	0.30	0.04	-0.01	RDKit	Lowest RMSD	7	Highest MR	23j	15	false	true
220	0.62	0.07	-0.15	InterLig		15	Highest MR	23j	28	false	true
221	0.99	-0.12	-0.26	AlignIt	Tanimoto	12	Highest MR	23j	8	false	true
222	0.66	-0.25	-0.23	AlignIt	Tanimoto	7	Lowest LogP	21a	1	false	false
223	0.55	-0.13	0.17	ShapeIt	Tversky	10	Lowest HA	22m	1	false	false
224	0.83	-0.16	-0.67	FKCOMBU	Volume Flex	12	Biggest	24j	2	false	true
225	0.62	-0.06	-0.05	RDKit	Tanimoto Distance	10	Lowest LogP	21a	6	false	true
226	0.79	-0.49	-0.22	FKCOMBU	Volume Flex	2	Highest LogP	23n	24	true	false
227	0.38	-0.13	0.16	RDKit	Tanimoto Distance	6	Most Active	23e	14	false	true
228	0.49	-0.01	-0.03	InterLig		11	Most Rigid	23d	29	false	true
229	0.37	-0.06	-0.20	InterLig		14	Highest LogP	23n	19	true	false
230	0.33	-0.10	0.19	ShapeIt	Tversky	2	Highest MR	23j	10	false	false
231	0.47	-0.05	-0.49	Shaep	OnlyShape	14	Most Active	23e	19	false	true
232	0.73	-0.09	-0.73	Shaep	Similarity	8	Most Active	23e	9	false	false
233	0.96	-0.09	0.30	RDKit	Tanimoto Distance	13	Most Active	23e	22	false	true
234	0.38	0.03	0.32	Shaep	OnlyShape	6	Biggest	24j	23	false	false
235	0.31	-0.07	-0.38	RDKit	Protrude Distance	7	Lowest HD	24j	14	false	true
236	0.57	0.08	-0.23	FKCOMBU	Volume Flex	11	Lowest HD	24j	27	false	true
237	0.45	-0.39	-0.28	FKCOMBU	Volume Flex	1	Highest LogP	23n	7	true	false
238	0.77	-0.31	-0.55	FKCOMBU	Atom Rigid	11	Lowest HA	24i	22	true	false
239	0.52	-0.17	0.17	Shaep	OnlyShape	3	Most Active	23e	7	true	false
240	0.43	-0.52	-0.03	Shaep	OnlyShape	8	Least Active	24e	29	false	true
241	0.38	-0.17	-0.20	FKCOMBU	Volume Rigid	14	Lowest LogP	21a	29	false	true
242	0.99	-0.09	-0.08	RDKit	Protrude Distance	13	Lowest LogP	21a	6	false	false
243	0.42	0.08	-0.44	InterLig		7	Lowest HD	24j	6	false	false
244	0.60	0.17	-0.11	AlignIt	Tanimoto	13	Least Active	24e	25	false	true
245	0.41	0.06	-0.21	Shaep	OnlyShape	6	Lowest HD	24e	26	false	true
246	0.77	-0.31	-0.31	InterLig		10	Lowest HD	24j	21	false	false
247	0.75	-0.08	-0.07	RDKit	Tanimoto Distance	14	Lowest LogP	21a	4	false	false
248	0.29	-0.01	-0.12	RDKit	Best Score	7	Highest LogP	23n	3	false	false
249	0.53	-0.07	0.23	AlignIt	Tanimoto	2	Lowest LogP	21a	13	false	true
250	0.43	-0.23	0.14	FKCOMBU	Volume Flex	5	Lowest HA	24c	20	true	false
251	0.40	-0.04	0.20	RDKit	Best Score	6	Lowest HA	24h	18	false	false
252	0.79	-0.03	-0.56	FKCOMBU	Volume Flex	7	Highest MR	23j	10	true	false
253	0.78	0.14	0.08	InterLig		10	Most Rigid	21c	8	false	true
254	0.32	-0.16	0.02	Shaep	OnlyShape	13	Most Rigid	22d	14	false	false
255	0.78	0.02	-0.10	RDKit	Lowest RMSD	12	Biggest	24j	24	false	false
256	0.46	0.07	-0.15	ShapeIt	Tanimoto	9	Biggest	24j	28	false	false
257	0.30	-0.12	0.13	ShapeIt	Tanimoto	2	Lowest HD	24e	9	false	true
258	0.88	-0.03	0.53	RDKit	Lowest RMSD	14	Least Active	24e	6	false	false
259	0.37	-0.21	-0.62	FKCOMBU	Atom Rigid	7	Lowest LogP	21a	30	true	false
260	0.49	-0.13	-0.14	InterLig		13	Lowest LogP	21a	23	true	false
261	0.46	0.13	0.08	Shaep	OnlyShape	13	Lowest HA	24h	11	true	false
262	0.58	-0.36	0.47	AlignIt	Tanimoto	11	Lowest LogP	21a	25	false	true
263	0.78	0.02	-0.10	RDKit	Tanimoto Distance	12	Biggest	24j	15	true	false
264	0.44	-0.45	-0.51	FKCOMBU	Atom Rigid	10	Lowest HA	24m	8	false	true
265	0.76	0.02	-0.15	RDKit	Best Score	15	Least Active	24e	0	false	false
266	0.38	-0.28	-0.12	RDKit	Best Score	10	Lowest HD	24e	4	false	true

Model ID	$r^{2,a}$	q^2_{cv}[b]	q^2_{TS}[c]	Alignment[d]		Conf Search ID[e]	Reference Mol[f]		Conformation[g]		
				Method	Scoring Function		Parameter	Molecule	Reference	Longest	Biggest
267	0.49	-0.34	-0.05	Shaep	Similarity	10	Highest MR	23j	29	false	true
268	0.69	-0.08	0.22	RDKit	Lowest RMSD	15	Lowest HD	24j	9	false	false

a: coefficient of determination
b: cross-validated coefficient
c: predictive determinant coefficient from TS predictions
d: alignment software and scoring function as listed in Supporting Information Table S3
e: Conformation IDs as listed in Supporting Information Table S2
f: Reference molecule selected by the properties and parameters listed in Supporting Information Tables S4 and S5, respectively.
g: selection of conformation used as template during the alignment. When Longest and Biggest were both false the global minima conformation was used. When Longest was true the longest conformation of the molecule was used and when Biggest was true the conformation with the highest volume was used.

Table S7. Overview of the selected model 13

PC	r^2	SDEC	q^2_{CV}	$SDEP_{CV}$	q^2_{TS}	$SDEP_{TS}$	r^2_{YS}	$SDEC_{YS}$	q^2_{YS}	$SDEP_{YS}$
1	0.52	0.41	0.07	0.57	-0.11	0.61	0.37	0.47	0.04	0.58
2	0.73	0.31	0.29	0.50	0.03	0.58	0.55	0.40	0.07	0.57
3	0.81	0.26	0.33	0.49	0.18	0.53	0.83	0.25	0.28	0.50
4	0.88	0.21	0.36	0.47	0.34	0.47	0.87	0.21	0.09	0.56
5	0.93	0.16	0.36	0.47	0.41	0.45	0.88	0.20	0.21	0.53
6	0.96	0.12	0.39	0.46	0.32	0.48	0.90	0.19	0.16	0.54
7	0.97	0.10	0.38	0.47	0.32	0.48	0.93	0.16	0.11	0.56
8	0.98	0.08	0.36	0.47	0.35	0.47	0.97	0.10	0.03	0.58

Table S8. Model 13 Training Set Experimental, Recalculated and Cross-validated pID_{50}s. Also are reported TS and VS Experimental and Predicted pID_{50}s.

Mol Name	Exp	TR	CV	TS	VS	Mol Set
**21a**	6.98	7.07	7.22			Training Set
**22b**	7.55	7.56	7.44			Training Set
**22d**	7.96	8.08	7.77			Training Set
**22f**	6.84	6.79	6.64			Training Set
**22h**	7.02	6.66	6.51			Training Set
**22n**	6.77	7.02	7.44			Training Set
**23a**	6.41	6.21	6.36			Training Set
**23b**	7.03	7.08	7.26			Training Set
**23c**	7.38	7.40	7.37			Training Set
**23d**	8.00	7.70	7.30			Training Set
**23g**	6.19	6.10	6.31			Training Set
**23j**	6.75	6.75	6.74			Training Set
**23m**	6.38	6.39	6.69			Training Set
**24i**	7.70	7.82	7.50			Training Set
**24l**	7.92	7.89	6.94			Training Set
**24m**	7.85	7.81	7.37			Training Set
**21b**	8.10	7.84	7.41			Training Set
**21f**	7.13	6.81	6.67			Training Set
**21g**	7.57	7.57	7.30			Training Set
**21h**	7.10	7.07	7.43			Training Set
**21l**	7.02	6.98	6.90			Training Set
**22a**	6.57	7.00	7.42			Training Set
**22g**	7.10	6.96	6.86			Training Set
**22m**	6.92	6.92	6.98			Training Set

Mol Name	Exp	TR	CV	TS	VS	Mol Set
**23e**	8.30	8.31	7.21			Training Set
**23k**	6.22	6.45	6.65			Training Set
**23l**	6.40	6.44	7.02			Training Set
**24a**	6.46	6.72	7.54			Training Set
**24e**	6.89	7.02	7.52			Training Set
**24g**	7.08	6.99	7.09			Training Set
**24j**	7.59	7.55	7.10			Training Set
**22k**	7.13	6.93	6.49			Training Set
**23i**	6.59	6.76	6.85			Training Set
**23n**	6.03	6.16	6.44			Training Set
**21n**	6.84	6.70	6.58			Training Set
**24c**	7.01	6.86	7.32			Training Set
**23f**	6.28	6.51	6.69			Training Set
**22e**	8.22	8.35	7.57			Training Set
**22j**	7.15	7.21	7.22			Training Set
**21c**	7.68	7.69	7.77			Training Set
**24h**	7.72	7.73	7.61			Training Set
**22l**	6.90	6.87	6.83			Training Set
**21k**	7.37			7.22		Test Set
**23h**	6.44			7.23		Test Set
**24f**	7.32			7.01		Test Set
**24n**	7.43			7.24		Test Set
**24o**	7.24			6.64		Test Set
**21m**	6.98			6.84		Test Set
**22i**	7.09			7.30		Test Set
**24b**	7.30			7.14		Test Set
**21e**	8.70			7.83		Test Set
**21o**	6.73			7.23		Test Set
**21i**	7.09			7.20		Test Set
**21d**	8.22			7.81		Test Set
**23o**	6.36				6.56	3D Validation Set
**24d**	6.98				7.14	3D Validation Set
**22c**	7.51				7.75	3D Validation Set
**24k**	7.59				7.60	3D Validation Set
**21j**	7.15				7.16	3D Validation Set
**22o**	6.40				6.41	3D Validation Set

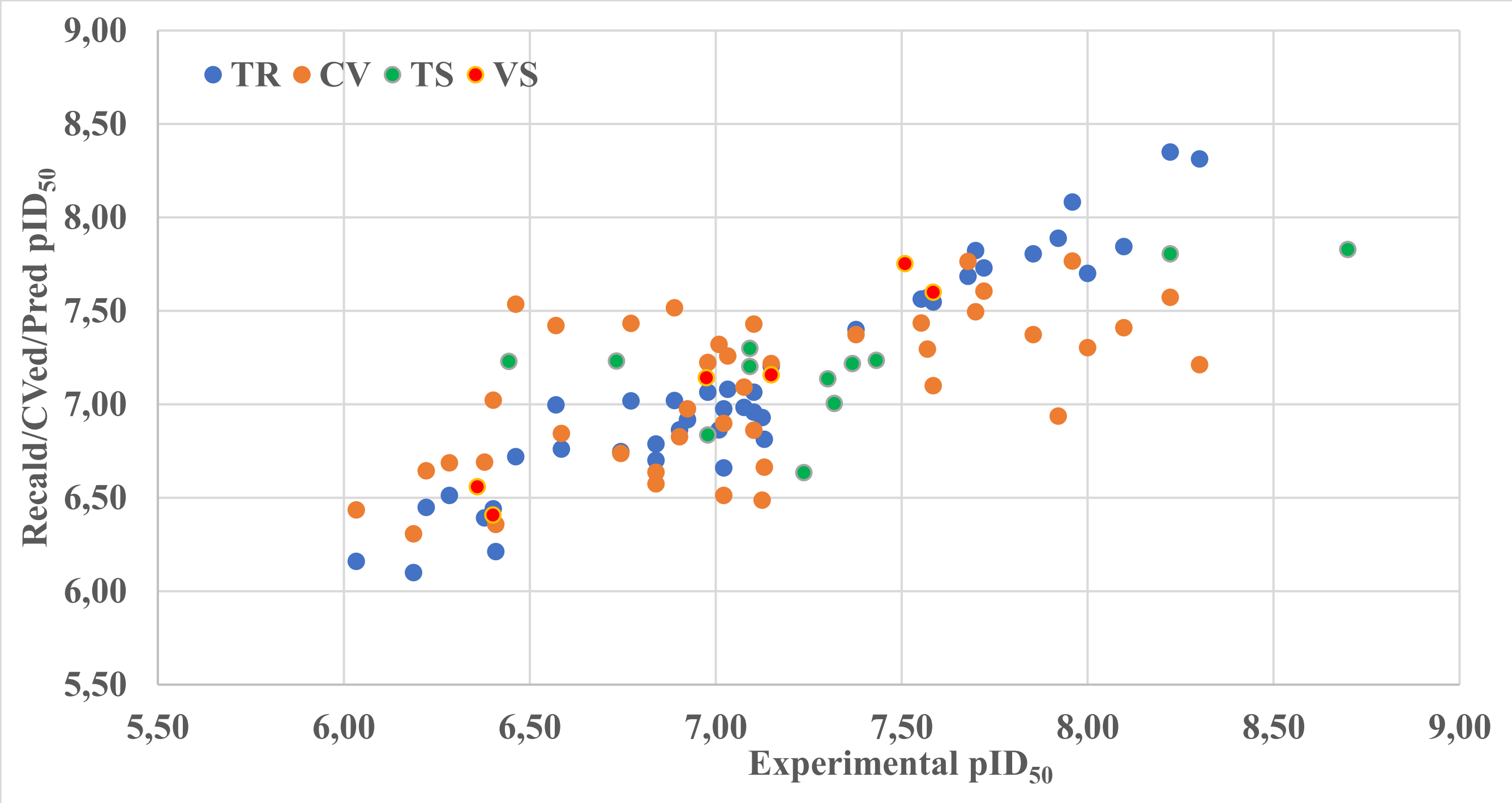

Figure S1. Scatter Plot of Model 13 Training Set Experimental, Recalculated (blue points) and Cross-validated (orange points) pID_{50}s. Also are reported TS (orange points) and VS (red points) Experimental and Predicted pID_{50}s.

2.1.2.1.4. Py-CoMFA contour maps analysis.

The above obtained models were then graphically inspected through the average activity contribution (AAC) plots. Regarding the selected model (ID 13), the aligned molecules overlapped with the steric and electrostatic CoMFA-like maps reveal distinct spatial zones where molecular modifications can either enhance or diminish activity. AAC maps reflect average contributions derived from the product of the average molecular interaction fields over the TS and the PLS regression coefficients. The resulting polyhedral, green and yellow for steric, blue and red for electrostatic, indicate where structural changes are likely to have a positive or negative impact on potency (MIC). Steric (STE) maps (green regions, Supporting Information Figure S2) represent areas where increasing steric bulk enhances activity, while yellow regions highlight zones where bulky substituents are detrimental for the activity. Whereas electrostatic (ELE) maps (blue polyhedron, Supporting Information Figure S2) indicate favorable regions where appropriately positive or negative charged groups enhance activity, while red polyhedron represent unfavorable regions where positive or negative appropriately charged group reduce activity. In particular the most and least potent compounds (**21e** and **23n**) against WT HIV1-RT overlapped with STE and ELE AACs polyhedron revealed that positive steric features (green polyhedron, panel A of Supporting Information Figure S3) are mostly associated with the 2,6-difluoro substituted benzyl moiety at uracil C6 position, the orientation of the uracyl carbonyl and the terminal phenyl group connected through the thiomethylthio chain at C2. Negative steric maps (yellow polyhedron**,** panel A of Supporting Information Figure S3) can be associate mainly to the presence of steric hindrance substituents at position C5 of the uracil ring (isopropyl of **23n**), shifted orientation of the benzyl moiety at uracil C6 position that might occur in the presence of substituents bulkier than fluorine atoms and a third important negative effect is related to the steric hindrance variability alkyl/phenylthiomethyl sidechain as less bulky group during the alignment induced the shift of the uracil ring far from the green polyhedral close to the uracil carbonyl group (Supporting Information Figure S2). Regarding the ELE contribution positive areas (blue polyhedron, panel B of Supporting Information Figure S3) were observed around the carbonyl group of both **21e** and **23n** and one of the orto-substituent on the benzyl group at uracil C6 position. On the other hand, negative ELE contribution are mainly related to the nature of the bridging sidechain at position C2 indicating

that likely less charged atom would be preferably. A small red polyhedral confirm that a shifted uracil carbonyl group might have negative impact on the anti-RT potency.

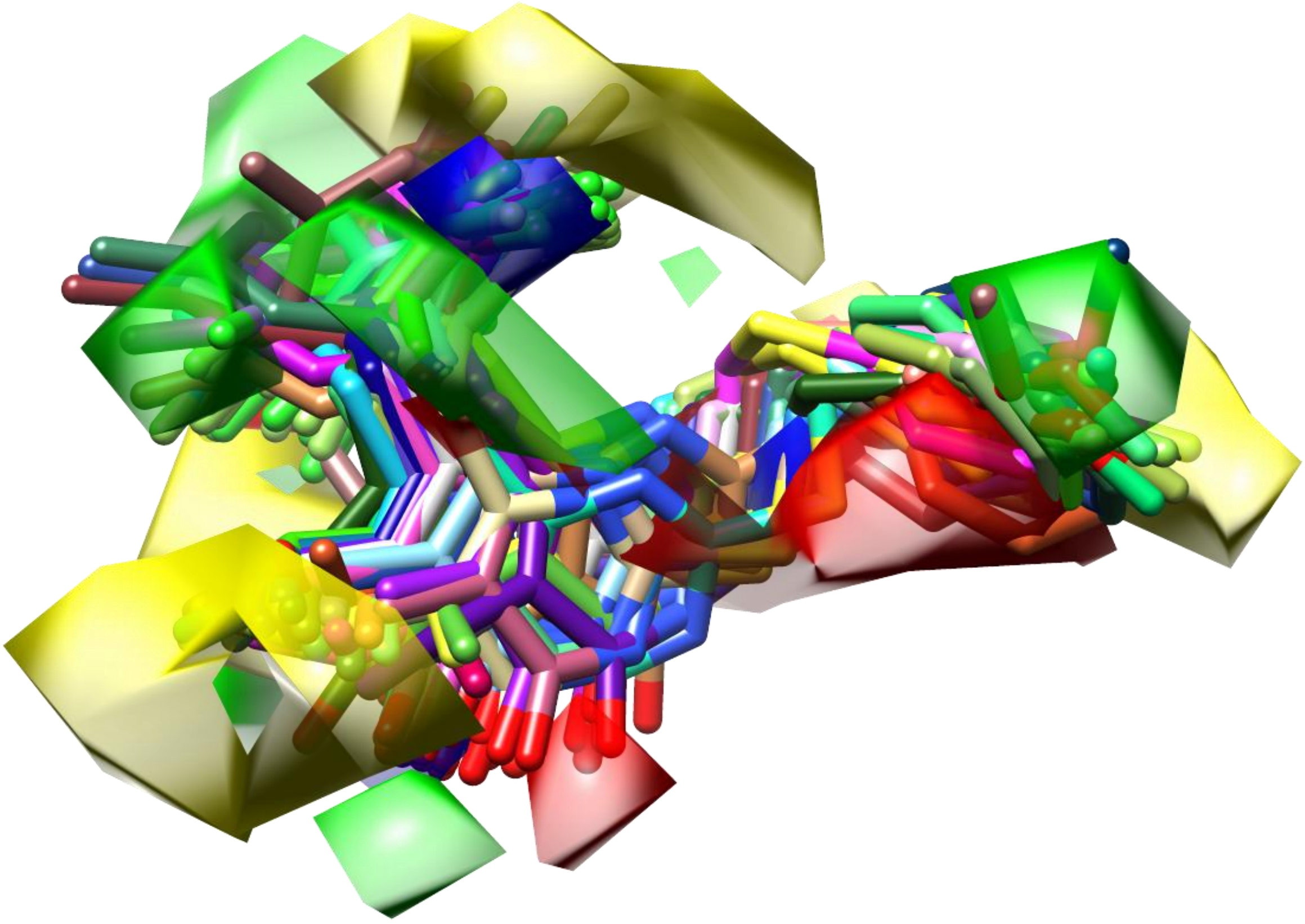

Figure S2. TR and TS mols overlapped with STE and ELE AACs.

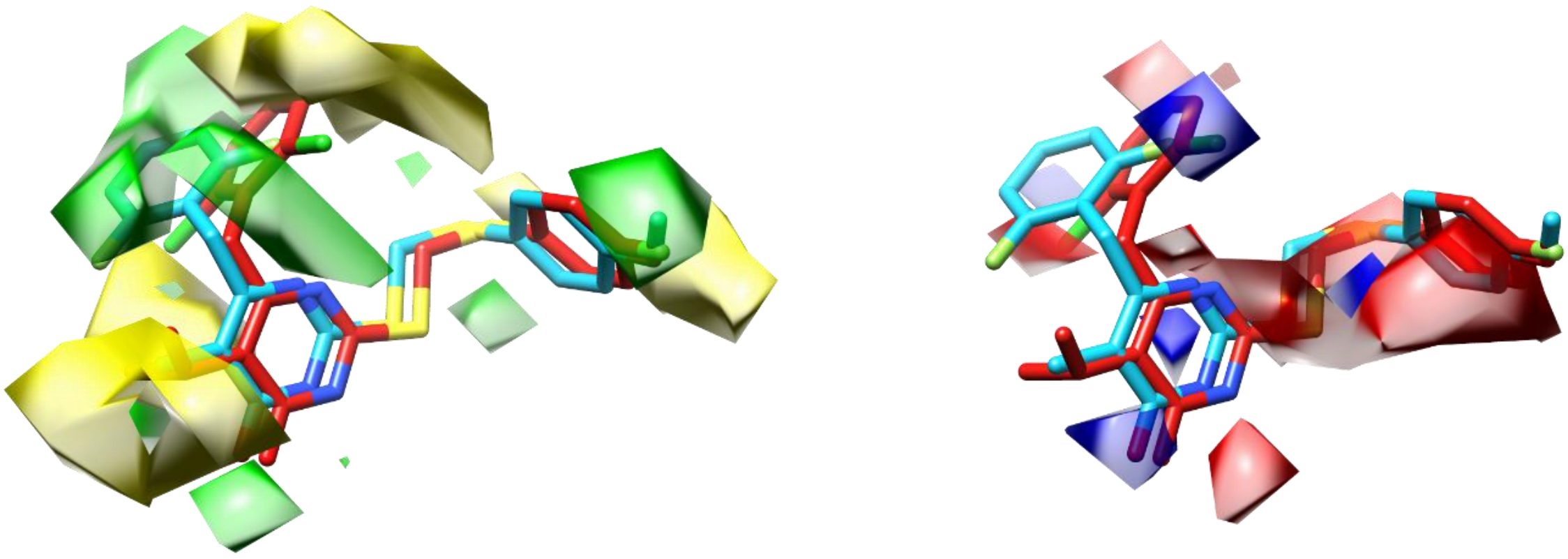

A B

Figure S3. Compounds **21e** and **23n** overlapped with STE (panel A) and ELE (panel B) AACs.

2.1.2.1.5. 3-D QSAR predictive ability.

The selected model 13 (Supporting Information Tables S6-S8) was also validated against the VS and showed to have an average absolute error of prediction as low as 0.11 pID_{50} units.

2.1.3. SB approaches.

Structure-based (SB) molecular modeling procedures were applied to investigate the binding mode of the newly synthesized compounds **21-24** through molecular docking and comparative binding energy (COMBINE) analysis.

2.1.3.1. Molecular docking assessment.

A docking assessment procedure was performed using the last version of the AutoDockVina docking program[4-7, 14, 15] (Version 1.2.7) with the vina scoring function and as previously reported confirmed to be suitable to redock random generated conformation of experimental complexed ligands displaying root mean square deviations (RMSD) in the range of 0.25-0.84 (Supporting Information Table S9).

Table S9. Assessment of AutoDockVina by random re-docking of Nevirapine and Efavirenz into WT and six mutated RTs .

Key*	Lock*	pID_{50}	Vina Score	RMSD
1fk9.V106A.key	1fk9.V106A	7.40	-10.90	0.34
1vrt.Y188L.key	1vrt.Y188L	4.74	-9.02	0.84
1fk9.Y188L.key	1fk9.Y188L	6.42	-10.77	0.25
1s1u.L100I.key	1s1u.L100I	5.05	-9.96	0.30
1fk9.L100I.key	1fk9.L100I	6.92	-10.88	0.43
1fk9.V179D.key	1fk9.V179D	7.00	-11.10	0.41
1vrt.Y181I.key	1vrt.Y181I	4.44	-9.41	0.60
1fk9.WT.key	1fk9.WT	7.52	-11.19	0.40
1fk9.Y181I.key	1fk9.Y181I	6.82	-11.57	0.33
1fkp.K103N.key	1fkp.K103N	5.15	-11.70	0.54
1fko.K103N.key	1fko.K103N	6.80	-12.05	0.50
1vrt.V106A.key	1vrt.V106A	5.00	-9.01	0.70
1vrt.V179D.key	1vrt.V179D	5.70	-9.72	0.65
1vrt.WT.key	1vrt.WT	6.40	-9.80	0.73

* ligand (key) and protein (lock) names are retained from the previous reports. [4-7, 14, 15]

2.1.3.2. COMBINE modeling.

A previously described COMBINE procedure showed good performances.[4-7, 14, 15] Using original training set of modeled complexes of nevirapine and efavirenz complexed into seven different HIV-RT enzymes (WT, L100I, K103N, V106A, V179D, Y181I, Y188L) was used.

The model was replicated using the Py-ComBinE procedure available in the 3d-qsar.com portal[3, 8] To develop the Py-ComBinE model in the actual implementation four type of ligand/residues interactions (STE, ELE, DRY and HB) are calculated by means of a modified "write_component_energies.py" code available from the AutoDockTools4[24]. Application of the partial least square (PLS) and leave-one out (LOO) cross-validation (CV) available from Scikit-learn[25]. The new model was obtained through a simulated annealing (SA) feature selection, that due to the stochastic nature of SA was run three times and characterized by average values of r^2 and $q^2_{LOO\text{-}CV}$ coefficients of 0.798±0.002 and 0.659±0.003 (Supporting Information Table S10), respectively, in a good agreement with the previous reported COMBINEr model[7].

Table S10. SA feature selection optimized Py-ComBinE models.

#[a]	PC[b]	$r^{2,c}$	SDEC[d]	q^2_{CV}[e]	$SDEP_{CV}$[f]
SA 1	2	0.797	0.456	0.656	0.594
SA 2	2	0.800	0.453	0.660	0.591
SA 3	2	0.798	0.456	0.661	0.590
Average	2	0.798±0.002	0.455±0.002	0.659±0.003	0.592±0.002

[a]: SA optimized models and its average

[b]: Number of optimal PLS principal components

[c]: Coefficient of determination calculated on the pIC_{50} values recalculated on the training set

[d]: Standard Deviation Error of Calculation in fitting.

[e]: Cross-validated coefficient of determination calculated on the pID_{50} values calculated on the during CV.

^{f}: Standard Deviation Error of Prediction during CV.

Analysis of the model average activity contributions plot (Supporting Information Figure S4) obtained by the PLS pseudo coefficients and per residue ligand/protein interactions product revealed that the inhibitory potency is mostly modulated by the hydrogen bonding establishment with Lys101 residue of chain A (P66 subunit) and at less extent with residue 181 of chain A. These data are in very good agreement with the reported SB-based structure-activity for NNRTIs.

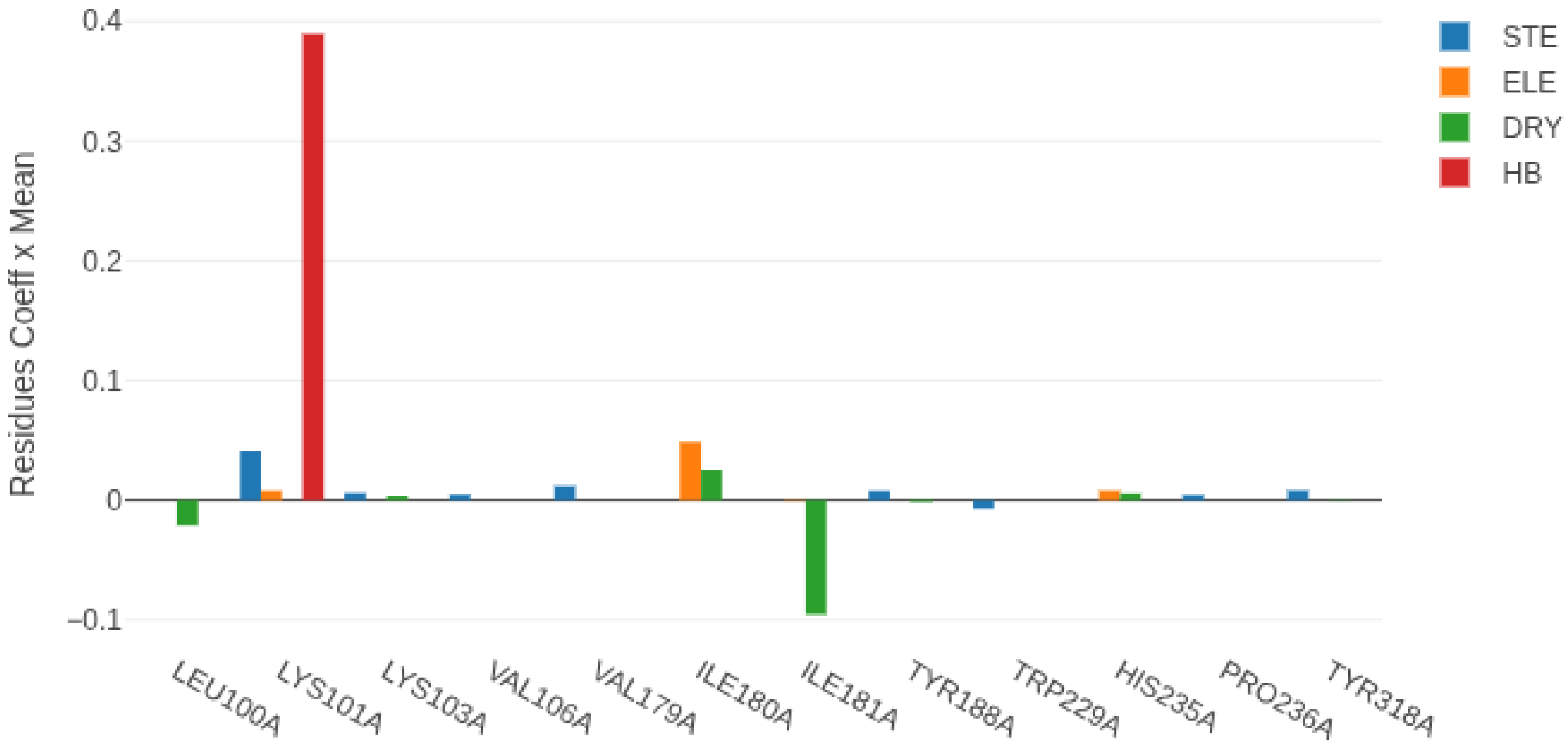

Figure S4. Plot of the most important Coeff*ResidueAverageInteraction (Average activity contribution). In figure is reported ILE181A, but it should be read TYR181A, as well as for LEU100A (I100A) and LY103A (ARG103A)

The new Py-ComBinE model enables the profiling at molecular level of the intercorrelation and the interactions between the herein experimentally investigated HIV-1 RT potencies of compounds **21-24** against investigated WT HIV-1 RT and mutations (Table 3)

2.1.3.2.1. Putative binding mode of the new compounds.

As not all compounds exhibited activity against all HIV-1 RT forms only 17 of them were docked (Supporting Information Table S10) revealing the compounds to be highly susceptible to the HIV-1 RT single-point mutations.

Binding modes of **21-24** derivatives into WT RT overlapped with those of previously reported 2-chloro-6-fluoro *S*-DABOs[5], and *NH*-DABOs[6] and their α-Methoxy[4] analogues (Supporting Information Figures S5-S8). To avoid any redundancy, only the binding mode of the most potent derivative **21e** is reported and compared to Efavirenz. In particular, **21e** placed into WT RT almost the same interaction already described for 2-chloro-6-fluoro *S*–DABO, with the 2,6-difluorobenzyl moiety that was located in the aromatic-rich pocket mainly delimited by Pro95A, Tyr181A, Tyr188A, Phe227A and Trp229A. The uracil ring was accommodated in a partially hydrophobic pocket mainly formed by the mainchain part of Lys101, Lys102 and Lys103 and a hydrogen bond is formed between NH at position 3 and the Lys101 carbonyl (distance of 3.02 Å). A few differences were observed for the sidechain at uracil position C2. The extended sidechain was predicted to bind into an open channel of the non-nucleoside binding site (NNBS) setting a number of van der Waals interactions with sidechains of Glu138A and Val179A and mainchain of Lys101A and Lys102A (Panel B of Supporting Information Figures S5).

A different scenario was instead observed for the predicted binding of **21e** into the mutated RTs (Panel B of Supporting Information Figures S6-S8). In K103N mutated isoform, although showing a good level of inhibiting potency (0.021 μM, Table 3) the docked conformation of **21e** did not maintain any of the interactions observed for into the WT enzyme (Panel B of Supporting Information

Figure S6). The *p*-methoxyphenyl group was found in the aromatic-rich pocket establishing a good π-π interaction with Trp229A; the uracil is moved far from the three lysine towards Val106A, Pro236A, Phe227A and His235A; the 2,6-difluorobenzyl was observed in a hydrophobic part of the NNBS mostly delimited by Val106A, Val179A, Ile180A and Val189A. Into Y181I mutated RT **21e** displayed the worse situation and this was in good agreement with the 2150-fold resistance ratio (Table 3). The rich aromatic pocket was left empty and actually the proposed docked conformation was not reproducible, indicating a very weak anchoring of the molecule inside the NNBS (Panel B of Supporting Information Figures S7). Again, also for the L100I mutant the docked conformation of **21e** was not reproducible and thus any detailed interaction would be purely speculative (Panel B of Supporting Information Figure S8).

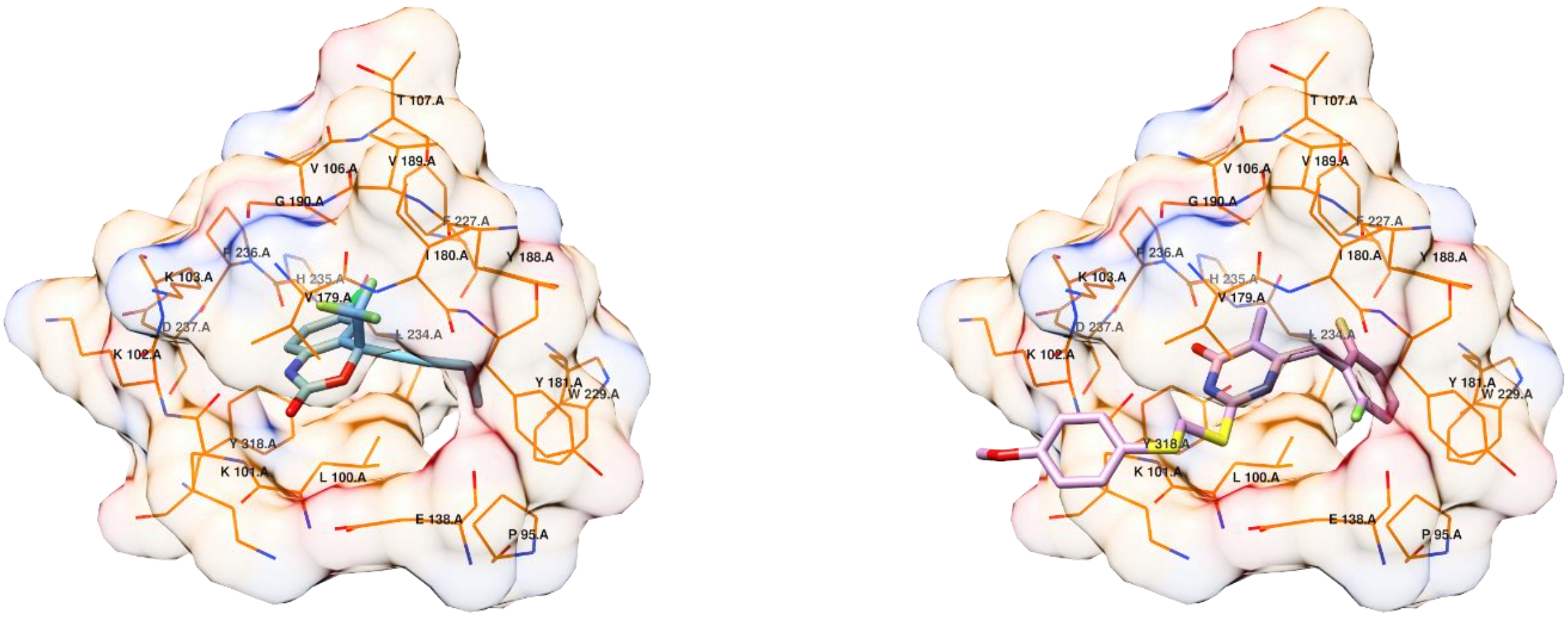

A B

Figure S5. Experimental bound conformation of Efavirenz (Panel A) compared to the docked conformations of **21e** (Panel B) into WT HIV-1 RT.

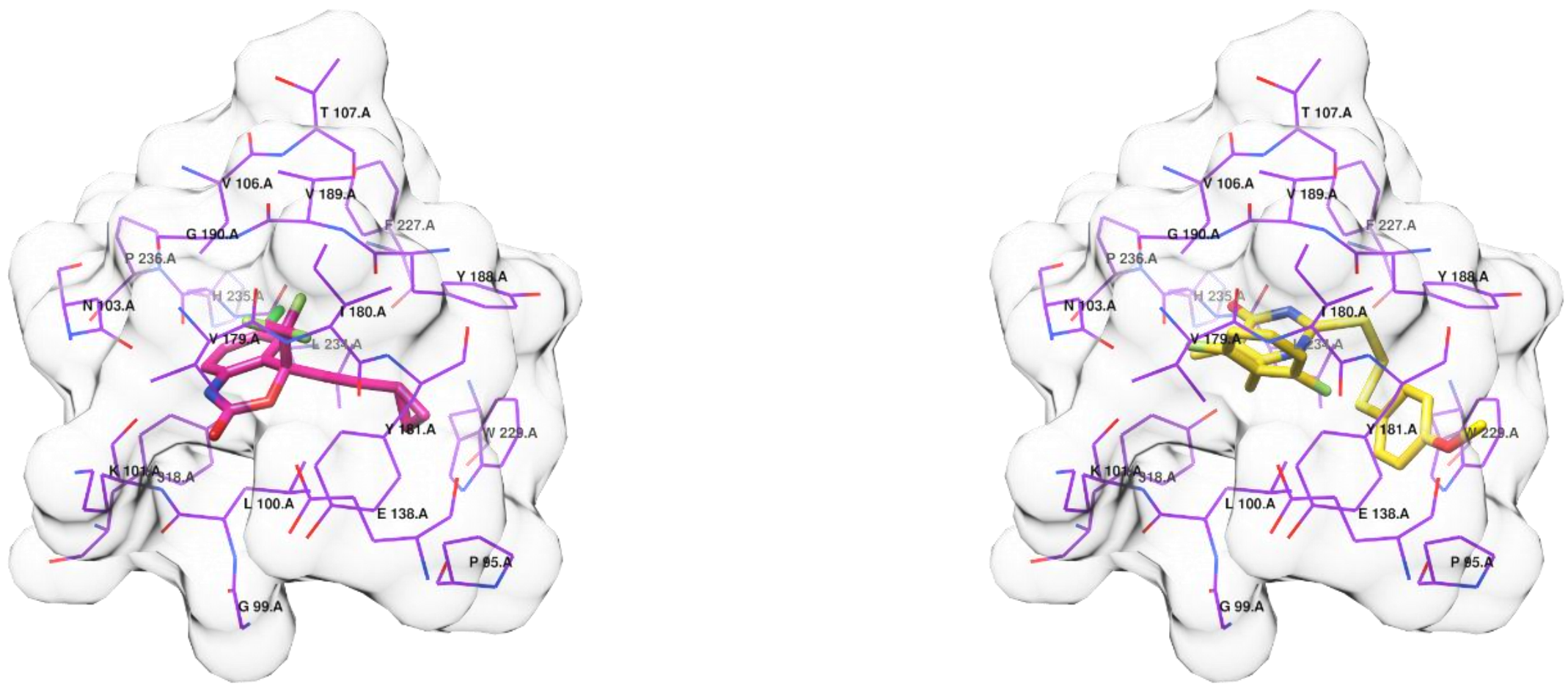

A B

Figure S6. Experimental bound conformation of Efavirenz (Panel A) compared to the docked conformations of **21e** (Panel B) into K103N HIV1-RT.

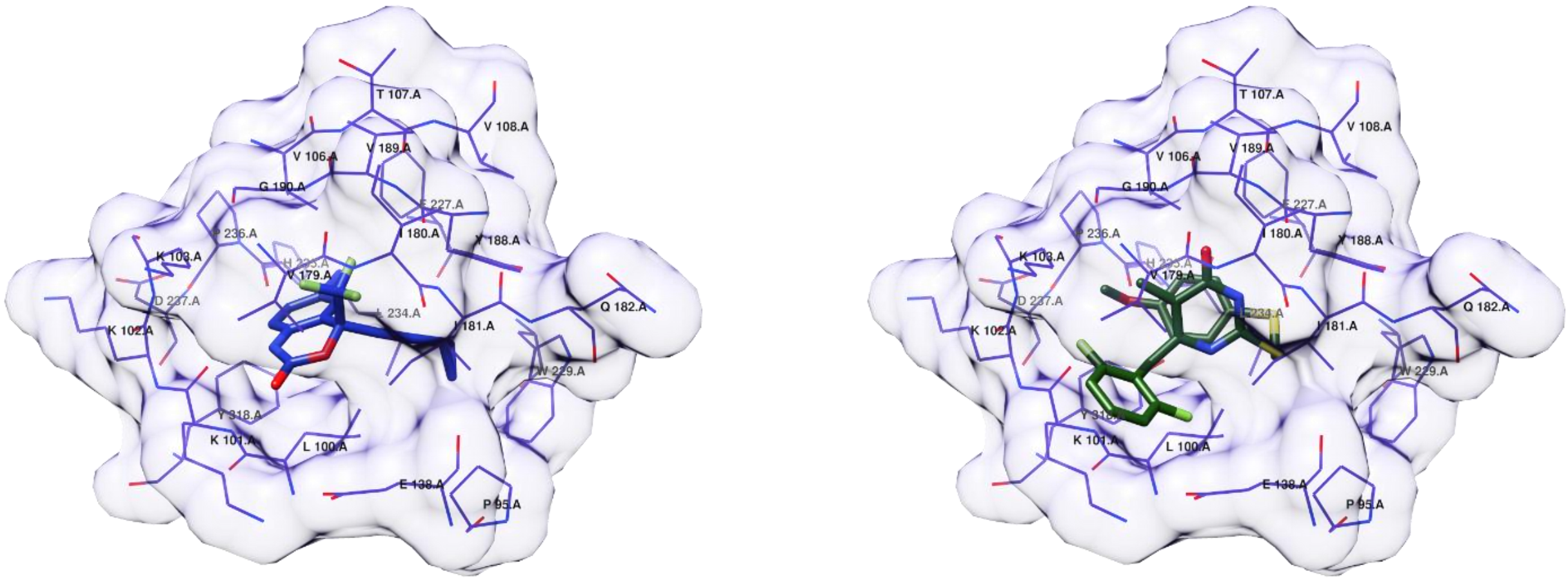

Figure S7. Experimental bound conformation of Efavirenz (Panel A) compared to the docked conformations of **21e** (Panel B) into Y181I HIV1-RT.

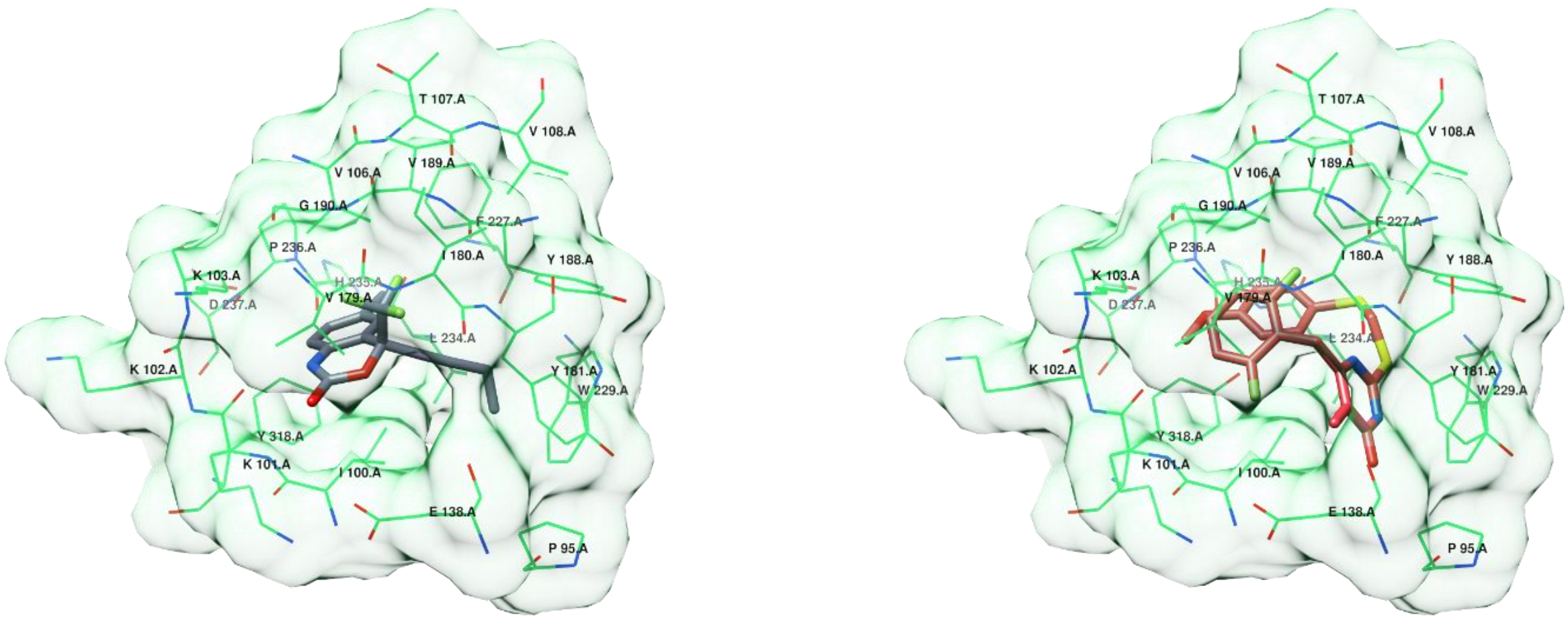

Figure S8. Experimental bound conformation of Efavirenz (Panel A) compared to the docked conformations of **21e** (Panel B) into L100I HIV1-RT.

2.1.3.2.2. <u>Prediction of protein affinity of new compounds.</u>

The COMBINE model was applied to the docked conformation of compounds **21-24** obtained in the WT and mutated HIV-1 RTs listed in Table 3. As not all compounds exhibited activity against all HIV-1 RT isoforms, only 17 of them were docked and predicted. In agreement with previous reports, the COMBINE model proved to be quite good in predicting the potency profile of the 17 listed compounds (Supporting Information Table S10). The COMBINE model predicted the 17 compounds with acceptable absolute errors, with an average absolute error of 0.87, 1.22, 0.92 and 0.43 pID50 units for WT, K103N, Y181I and L100I forms of RT, respectively. These results support the above-described binding mode and strengthen the validity of the COMBINE procedure.

Table S11. Predicted pID_{50} for the 17 compounds that showed activities against all WT and mutated isoforms of HIV-1 RT. The average absolute error (AAE) and standard deviation error of prediction (SDEP) are also reported.

**Compd**	**Experimental pID_{50}**				**Predicted pID_{50}**			
	**WT RT**	**K103N**	**Y181I**	**L100I**	**WT RT**	**K103N**	**Y181I**	**L100I**
**21c**	7.68	6.35	5.04	5.82	7.07	8.78	5.57	5.71
**21d**	8.22	7.05	5.29	6.17	7.13	7.63	5.36	4.99
**21e**	8.70	7.36	5.37	6.26	8.23	7.81	6.20	5.95

**21h**	7.10	4.77	4.89	5.68	6.87	6.68	6.28	5.80
**21i**	7.09	5.12	4.87	5.77	6.95	6.55	5.91	5.44
**22c**	7.51	5.08	4.99	5.59	7.03	6.46	6.60	5.78
**22d**	7.96	6.20	5.19	5.62	7.27	6.11	6.59	5.09
**22e**	8.22	7.11	5.54	5.92	7.03	7.20	6.73	6.21
**22h**	7.02	4.78	4.89	5.68	7.09	7.71	6.72	5.93
**22i**	7.09	5.13	4.87	5.80	7.26	6.51	6.42	5.39
**23c**	7.38	6.02	5.11	5.64	6.94	5.57	5.06	5.90
**23d**	8.00	6.35	5.37	5.74	7.25	5.32	6.83	5.14
**23e**	8.30	7.35	5.85	5.96	4.88	4.69	6.60	6.43
**24g**	7.08	5.01	5.18	5.54	6.57	5.99	5.49	6.01
**24k**	7.59	5.14	4.71	5.74	6.18	5.99	5.57	6.59
**24l**	7.92	5.25	5.67	6.36	6.25	5.68	5.78	6.91
**24m**	7.85	5.20	5.66	6.01	6.32	3.60	6.32	6.43
**AAE**					0.87	1.22	0.92	0.43
**SDEP**					0.85	1.43	0.58	0.52

3. Experimental Section

3.1. Computational Medicinal Chemistry

The LB and SB procedures were developed through the following points:

3.1.1. *Dataset Composition.*

The ligand-based training, test and validation sets were drawn in the Py-MolEdit module of the 3d-qsar.com portal using the embedded the free for academics Molsoft HTML5 Molecule Editor (https://www.molsoft.com/moledit.html).

3.1.2. *Generation of 3D conformations for each dataset molecule*.

Each of the training and test set molecules were stored as SMILES and transformed into 3-D local minima conformations by means of the obmin command available in the openbabel[34, 35] software.

3.1.3. *3-D QSAR modeling*.

3.1.3.1. *Conformational analyses*:

Each of the training and test set molecules was subjected to 16 different conformational analyses by means of three different procedures. In particular, with the software Balloon and two python scripts, all included in the Py-ConfSearch module of the 3d-qsar.com portal. For each procedure all the available force fields were used.

3.1.3.2. *Molecular alignments*:

Through a list of 17 pre-selected templates from each conformational analysis and the six available molecular alignment software as embedded in the 3d-qsar.com portal, thousands of combinations were possible. To reduce CPU time 5% of the possible alignments were randomly selected to obtain an equal number of aligned datasets.

3.1.3.3. *Calculation of MIFs and Py-CoMFA modeling*:

Calculation of MIFs was achieved using the default settings using the Py-CoMFA module of 3d-qsar.com. PLS-based 3D QSAR models were built for all the aligned datasets and evaluated by r^2, q^2_{cv} and q^2_{pred} values. Since good coefficients level were obtained optimization of the top model was not necessary. The best model was used to predict the MIC of the designed compounds.

3.1.4. *SB approaches.*

A structure-based protocol was applied to investigate the likely binding mode of Sudoterb and the herein designed derivatives into MmpL3 as potential biological target.

3.1.4.1. *Molecular docking assessment:*

Through exhaustive re-docking procedures the program AutoDockVina was assessed showing low RMSD values in the reproduction of the experimental complexes.

3.1.4.2. *COMBINE modeling*

The COMBINE procedure, as originally described requires that all proteins should be composed of the same number and positions of the residues. To achieve this condition compounds **21**-**24** were

docked in the RTs without any alteration of the proteins. Through a python script the per residue energy interactions were computed for each ligand, associated with the experimental pID_{50}s and subjected to PLS analysis and cross-validation to build the COMBINE models. The best model was finally used to predict the pID_{50}s of the synthesized compounds. All the procedure was run through the Py-ComBinE module of the 3d-qsar.com portal.

Data and Software Availability

Upon request to rino.ragno@uniroma1.it all data used for the developing the 3-D QSAR, COMBINE models and molecular docking results can be requested. With the data, any non profit user will be enabled to run Py-CoMFA and Py- ComBinE predictions as well as perform molecular docking on the www.3d-qsar.com portal.

References

1. Ragno, R.; Esposito, V.; Di Mario, M.; Masiello, S.; Viscovo, M.; Cramer, R. D., Teaching and Learning Computational Drug Design: Student Investigations of 3D Quantitative Structure-Activity Relationships through Web Applications. *J Chem Educ* **2020,** *97* (7), 1922-1930.
2. Sabatino, M.; Rotili, D.; Patsilinakos, A.; Forgione, M.; Tomaselli, D.; Alby, F.; Arimondo, P. B.; Mai, A.; Ragno, R., Disruptor of telomeric silencing 1-like (DOT1L): disclosing a new class of non-nucleoside inhibitors by means of ligand-based and structure-based approaches. *Journal of computer-aided molecular design* **2018,** *32* (3), 435-458.
3. Ragno, R., www.3d-qsar.com: a web portal that brings 3-D QSAR to all electronic devices-the Py-CoMFA web application as tool to build models from pre-aligned datasets. *Journal of computer-aided molecular design* **2019,** *33* (9), 855-864.
4. Nawrozkij, M. B.; Forgione, M.; Yablokov, A. S.; Lucidi, A.; Tomaselli, D.; Patsilinakos, A.; Panella, C.; Hailu, G. S.; Kirillov, I. A.; Badia, R.; Riveira-Munoz, E.; Crespan, E.; Armijos Rivera, J. I.; Cirilli, R.; Ragno, R.; Este, J. A.; Maga, G.; Mai, A.; Rotili, D., Effect of alpha-Methoxy Substitution on the Anti-HIV Activity of Dihydropyrimidin-4(3 H)-ones. *J Med Chem* **2019,** *62* (2), 604-621.
5. Rotili, D.; Tarantino, D.; Nawrozkij, M. B.; Babushkin, A. S.; Botta, G.; Marrocco, B.; Cirilli, R.; Menta, S.; Badia, R.; Crespan, E.; Ballante, F.; Ragno, R.; Este, J. A.; Maga, G.; Mai, A., Exploring the role of 2-chloro-6-fluoro substitution in 2-alkylthio-6-benzyl-5-alkylpyrimidin-4(3H)-ones: effects in HIV-1-infected cells and in HIV-1 reverse transcriptase enzymes. *J Med Chem* **2014,** *57* (12), 5212-25.
6. Rotili, D.; Samuele, A.; Tarantino, D.; Ragno, R.; Musmuca, I.; Ballante, F.; Botta, G.; Morera, L.; Pierini, M.; Cirilli, R.; Nawrozkij, M. B.; Gonzalez, E.; Clotet, B.; Artico, M.; Este, J. A.; Maga, G.; Mai, A., 2-(Alkyl/aryl)amino-6-benzylpyrimidin-4(3H)-ones as inhibitors of wild-type and mutant HIV-1: enantioselectivity studies. *J Med Chem* **2012,** *55* (7), 3558-62.
7. Ballante, F.; Musmuca, I.; Marshall, G. R.; Ragno, R., Comprehensive model of wild-type and mutant HIV-1 reverse transciptases. *Journal of computer-aided molecular design* **2012,** *26* (8), 907-19.
8. Ragno, R.; Zwergel, C.; Valente, S.; Astolfi, R.; Lambona, C.; Proia, E.; Giuliani, L.; Franzblau, S. G.; Fioravanti, R.; Mai, A., Combined computational and classical medicinal chemistry procedure to disclose novel pyrrole-based compounds as potential antituberculosis agents. *Journal of computer-aided molecular design* **2026,** *40* (1).
9. Weininger, D., SMILES, a chemical language and information system. 1. Introduction to methodology and encoding rules. *Journal of Chemical Information and Computer Sciences* **1988,** *28* (1), 31-36.
10. O'Boyle, N. M.; Banck, M.; James, C. A.; Morley, C.; Vandermeersch, T.; Hutchison, G. R., Open Babel: An open chemical toolbox. *J Cheminform* **2011,** *3*, 33.
11. O'Boyle, N. M.; Morley, C.; Hutchison, G. R., Pybel: a Python wrapper for the OpenBabel cheminformatics toolkit. *Chem Cent J* **2008,** *2*, 5.
12. Ebejer, J. P.; Morris, G. M.; Deane, C. M., Freely available conformer generation methods: how good are they? *J Chem Inf Model* **2012,** *52* (5), 1146-58.
13. Cramer, R. D.; Patterson, D. E.; Bunce, J. D., Comparative molecular field analysis (CoMFA). 1. Effect of shape on binding of steroids to carrier proteins. *J Am Chem Soc* **1988,** *110* (18), 5959-67.
14. Eberhardt, J.; Santos-Martins, D.; Tillack, A. F.; Forli, S., AutoDock Vina 1.2.0: New Docking Methods, Expanded Force Field, and Python Bindings. *J Chem Inf Model* **2021,** *61* (8), 3891-3898.
15. Ragno, R.; Frasca, S.; Manetti, F.; Brizzi, A.; Massa, S., HIV-reverse transcriptase inhibition: inclusion of ligand-induced fit by cross-docking studies. *J Med Chem* **2005,** *48* (1), 200-12.
16. Vainio, M. J.; Johnson, M. S., Generating conformer ensembles using a multiobjective genetic algorithm. *J Chem Inf Model* **2007,** *47* (6), 2462-74.
17. Vainio, M. J.; Puranen, J. S.; Johnson, M. S., ShaEP: molecular overlay based on shape and electrostatic potential. *J Chem Inf Model* **2009,** *49* (2), 492-502.
18. Landrum, G., RDKit: Open-Source Cheminformatics Software. **2016**.
19. Taminau, J.; Thijs, G.; De Winter, H., Pharao: pharmacophore alignment and optimization. *J Mol Graph Model* **2008,** *27* (2), 161-9.
20. Grant, J. A.; Pickup, B. T., A Gaussian Description of Molecular Shape. *The Journal of Physical Chemistry* **1995,** *99* (11), 3503-3510.
21. Mirabello, C.; Wallner, B., InterLig: improved ligand-based virtual screening using topologically independent structural alignments. *Bioinformatics* **2020,** *36* (10), 3266-3267.
22. Kawabata, T.; Nakamura, H., 3D flexible alignment using 2D maximum common substructure: dependence of prediction accuracy on target-reference chemical similarity. *J Chem Inf Model* **2014,** *54* (7), 1850-63.
23. Bergstra, J.; Bengio, Y., Random search for hyper-parameter optimization. *Journal of Machine Learning Research* **2012,** *13*, 281-305.
24. Morris, G. M.; Huey, R.; Lindstrom, W.; Sanner, M. F.; Belew, R. K.; Goodsell, D. S.; Olson, A. J., AutoDock4 and AutoDockTools4: Automated docking with selective receptor flexibility. *J Comput Chem* **2009,** *30* (16), 2785-91.
25. Pedregosa, F.; Varoquaux, G.; Gramfort, A.; Michel, V.; Thirion, B.; Grisel, O.; Blondel, M.; Prettenhofer, P.; Weiss, R.; Dubourg, V.; Vanderplas, J.; Passos, A.; Cournapeau, D.; Brucher, M.; Perrot, M.; Duchesnay, É., Scikit-learn: Machine Learning in Python. *J. Mach. Learn. Res.* **2011,** *12* (null), 2825–2830.
